# Interactions between paralogous bacterial enhancer‐binding proteins enable metal‐dependent regulation of alternative nitrogenases in *Azotobacter vinelandii*

**DOI:** 10.1111/mmi.14955

**Published:** 2022-06-29

**Authors:** Corinne Appia‐Ayme, Richard Little, Govind Chandra, Carlo de Oliveira Martins, Marcelo Bueno Batista, Ray Dixon

**Affiliations:** ^1^ Department of Molecular Microbiology John Innes Centre Norwich UK; ^2^ Department of Biochemistry and Metabolism John Innes Centre Norwich UK

**Keywords:** alternative nitrogenases, bacterial enhancer‐binding proteins, biological nitrogen fixation, transcriptional regulation, vanadium

## Abstract

All diazotrophic bacteria and archaea isolated so far utilise a nitrogenase enzyme‐containing molybdenum in the active site co‐factor to fix atmospheric dinitrogen to ammonia. However, in addition to the Mo‐dependent nitrogenase, some nitrogen‐fixing prokaryotes also express genetically distinct alternative nitrogenase isoenzymes, namely the V‐dependent and Fe‐only nitrogenases, respectively. Nitrogenase isoenzymes are expressed hierarchically according to metal availability and catalytic efficiency. In proteobacteria, this hierarchy is maintained via stringent transcriptional regulation of gene clusters by dedicated bacterial enhancer‐binding proteins (bEBPs). The model diazotroph *Azotobacter vinelandii* contains two paralogs of the vanadium nitrogenase activator VnfA (henceforth, VnfA1), designated VnfA2 and VnfA3, with unknown functions. Here we demonstrate that the VnfA1 and VnfA3 bEBPs bind to the same target promoters in the *Azotobacter vinelandii* genome and co‐activate a subset of genes in the absence of V, including the structural genes for the Fe‐only nitrogenase. Co‐activation is inhibited by the presence of V and is dependent on an accessory protein VnfZ that is co‐expressed with VnfA3. Our studies uncover a plethora of interactions between bEBPs required for nitrogen fixation, revealing the unprecedented potential for fine‐tuning the expression of alternative nitrogenases in response to metal availability.

## Graphical abstract

In addition to using the conventional molybdenum nitrogenase to fix atmospheric nitrogen, some diazotrophic bacteria encode alternative nitrogenase isoenzymes, named the vanadium‐dependent and iron‐only nitrogenases respectively. Here we uncover a complex metal‐dependent regulatory network, which controls hierarchical expression of the alternative nitrogenases in *Azotobacter vinelandii*, through interactions between bacterial enhancer proteins that fine‐tune regulation of gene expression.

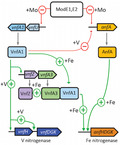

## 1 INTRODUCTION

The ability to carry out biological nitrogen fixation, a key process in the nitrogen cycle that underpins sustainable agriculture, is conferred in diverse bacteria and archaea by nitrogenase enzymes that catalyse the reduction of gaseous dinitrogen to ammonia. All diazotrophs that have been sequenced so far encode molybdenum nitrogenase with an active site co‐factor designated as FeMo‐co that contains a complex inorganic cluster [7Fe‐9S‐C‐Mo] in which the molybdenum atom is bound to a molecule of R‐homocitrate (Burén et al., 2020; Einsle & Rees, 2020). However, in addition to expressing the molybdenum nitrogenase enzyme, some diazotrophs also encode alternative nitrogenase isoenzymes, which can be utilised under molybdenum‐deficient conditions (Addo & Dos Santos, 2020; Bishop & Joerger, 1990; Harwood, 2020; Mus et al., 2018). The vanadium (Vnf) and iron‐only (Anf) nitrogenases have a component protein architecture similar to the molybdenum enzyme, but also have distinctive features. These enzymes contain catalytic components homologous to those of the molybdenum (NifHDK) enzyme, but have an additional subunit designated, δ, which is unique to alternative nitrogenases, resulting in four distinct structural subunits in the V and Fe‐only nitrogenases, named VnfHDGK and AnfHDGK respectively. In the vanadium nitrogenase, V replaces Mo in the active site co‐factor (FeV‐co) whereas, in the iron‐only nitrogenase, the Mo atom is replaced by Fe in the cofactor (FeFe‐co). The requirements for enzyme biosynthesis and maturation have been extensively studied in the model diazotroph *Azotobacter vinelandii*, which expresses all three nitrogenase enzymes. All three systems are reliant on genes located in *nif* gene clusters (associated with molybdenum nitrogenase) for the synthesis of homocitrate and NifB‐co, a core component of their active site cofactors (Burén et al., 2020; Kennedy & Dean, 1992; Zhao et al., 2007). However, the requirements for further maturation of the cognate co‐factors in each nitrogenase differ. Whereas the molybdenum (FeMo‐co) and vanadium (FeV‐co) co‐factors are matured on dedicated protein scaffolds provided by NifEN and VnfEN respectively, the assembly of the cofactor for the iron‐only enzyme (FeFe‐co) does not require these scaffolds and is likely to be completed on the AnfDK structural subunits (Pérez‐González et al., 2021; Yang et al., 2014).

The contribution of alternative nitrogenases to global nitrogen fixation is not fully understood. The molybdenum enzyme is the most catalytic efficient in vitro, whereas the V and Fe nitrogenases release more hydrogen as a by‐product of the reaction, with the Fe enzyme exhibiting the lowest ratio of dinitrogen reduction to hydrogen evolution (Harris et al., 2018, 2019). Since only a subset of diazotrophs encodes alternative nitrogenases, it has been suggested these enzymes merely provide a backup function in molybdenum‐limiting environments. However, recent studies have demonstrated that the vanadium enzyme can provide faster growth than the Mo nitrogenase in vivo, dependent on the nature of the carbon source (Luxem et al., 2020) and the Fe‐only enzyme has the unique ability to catalyse the combined reduction of carbon dioxide and dinitrogen to produce significant levels of methane in addition to ammonia, suggesting a potential role for this enzyme in shaping microbial community interactions in marine environments (Zheng et al., 2018). These discoveries in combination with nitrogenase activity measurements in various ecosystems (Bellenger et al., 2014; Darnajoux et al., 2019; McRose et al., 2017) suggest that alternative nitrogenases may provide critical functionality in some environmental niches (Harwood, 2020).

Under nitrogen‐limiting conditions, the expression of the three nitrogenase enzymes in *A. vinelandii* is stringently regulated in response to transition metal availability (Bishop & Joerger, 1990; Hamilton et al., 2011). Transcriptional regulation is facilitated by dedicated bacterial enhancer‐binding proteins (bEBPs) that activate transcription at RpoN (σ^54^)‐dependent promoters for Mo, (*nif*), V, (*vnf*) and Fe (*anf*) systems. Each of these proteins has a domain architecture typical of bEBPs including an N‐terminal regulatory GAF domain, a central catalytic AAA+ domain that employs ATP hydrolysis to remodel σ^54^‐RNA polymerase, and a C‐terminal DNA binding domain that interacts with enhancer sequences (Bush & Dixon, 2012; Gao et al., 2020). The master transcriptional activator for Mo nitrogenase and associated *nif* genes, NifA, is regulated by its partner protein NifL in response to the nitrogen, oxygen and carbon status (Bueno Batista et al., 2021; Dixon & Kahn, 2004), but NifA is apparently not regulated by molybdenum either at the level of expression or activity. In contrast, expression of the vanadium (VnfA) and Fe‐only (AnfA) bEBPs is repressed in the presence of molybdenum (Premakumar et al., 1998), providing a mechanism to prevent biosynthesis of alternative nitrogenases, when Mo is available. The presence of conserved cysteine residues in the N‐terminal regulatory GAF domains of VnfA and AnfA suggests a potential role in regulating the activity of these bEBPs in response to metals or redox status (Joerger et al., 1989). Notably, transcriptional activation by these proteins in vivo is abrogated when the cysteine residues are substituted (Nakajima et al., 2010; Premakumar et al., 1994). Spectroscopic analysis and in vitro Fe‐S cluster reconstitution experiments with purified VnfA and truncated variants suggest that it may contain a [Fe_3_‐S_4_] cluster in the GAF domain. However, when expressed in *Escherichia coli*, VnfA activity does not appear to be sensitive to oxygen in vivo, but is inhibited by reagents that chelate metals or that generate superoxide, potentially indicating a reactive oxygen sensing role for the Fe‐S cluster (Nakajima et al., 2010; Yoshimitsu et al., 2011). In contrast, transcriptional activation by AnfA in vivo requires *nifH*, which encodes the Fe protein component of molybdenum nitrogenase (Joerger et al., 1991). This dependency involves the GAF domain of AnfA since truncated proteins lacking the N‐terminal domain are fully active in the absence of Fe protein, suggesting that the GAF domain is involved in intramolecular repression of AnfA activity, which is relieved in the presence of Fe protein (Frise et al., 1994).

The genome sequence of *A. vinelandii* surprisingly revealed the presence of paralogs of nitrogen fixation regulatory bEBPs (Setubal et al., 2009). These include a gene homologous to *nifA*, designated *nifA2*, and two homologues of *vnfA* (which is henceforth referred to as *vnfA1*), designated *vnfA2* and *vnfA3* respectively. Although this does not necessarily imply that these paralogous bEBPs are involved in the regulation of the three nitrogenases, the sequence similarity of their DNA binding determinants to the cognate NifA and VnfA1 activators respectively, suggests that they may recognise the same enhancer sequences (Supplementary Figure S1). Furthermore, VnfA2 and VnfA3 contain conserved cysteines in their N‐terminal domains that are important for VnfA1 activity (Nakajima et al., 2010) and the pattern of regulation of *vnfA2* and *vnfA3* is similar to that of *vnfA1* since transcription of all three paralogs is repressed by the presence of molybdenum (Hamilton et al., 2011). In order to further understand the regulation of the alternative nitrogenases, we have combined ChIP‐seq analysis with genome‐wide transcript start‐site determination to define σ^54^‐dependent promoters activated by VnfA paralogs. We demonstrate that the expression of *vnfA2* and *vnfA3* is partly dependent on *vnfA1* and that VnfA1 and VnfA3 bind to the same target promoters. We also identify a subset of promoters that are co‐activated by VnfA1 and VnfA3 under iron‐only conditions but are repressed in the presence of V. This co‐activation requires another gene *vnfZ*, which is co‐transcribed with *vnfA3*. Regulation by VnfA paralogs also extends to the expression of the iron‐only nitrogenase to ensure that diazotrophic growth dependent on this enzyme only occurs in the absence of V. Using bacterial 2 hybrid analysis coupled with Co‐IP pull‐downs, we identify a multitude of interactions between paralogous bEBPs and with VnfZ that are likely to result in the formation of hetero‐oligomers to enable sophisticated fine‐tuning of alternative nitrogenase expression.

## 2 RESULTS

### 2.1 Molybdenum‐dependent regulation of the *vnfU, vnfA1* operon

**FIGURE 1:**
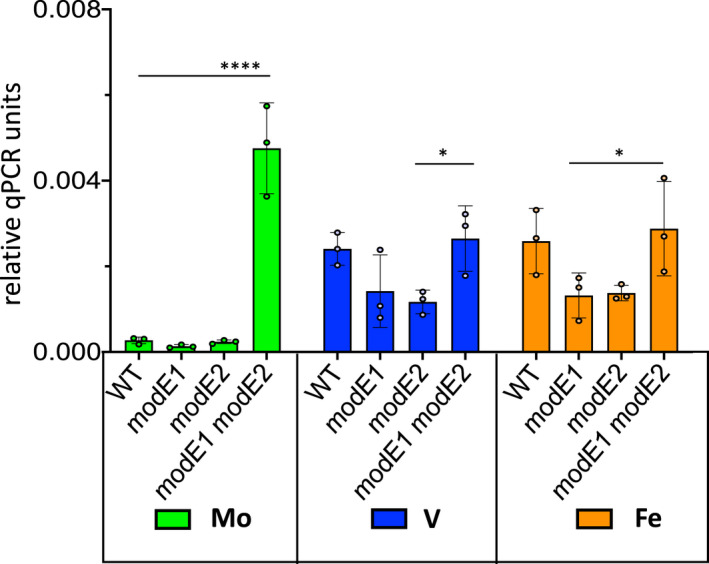
Influence of *modE* deletions on *vnfA1* transcripts as determined by qRT‐PCR. Strains were grown in ammonium excess conditions and then subjected to nitrogen step‐down in NIL medium for 6 h, either with no addition (iron‐only conditions, orange bars) or in the presence of molybdenum (green bars), or vanadium (blue bars) as indicated. Transcript levels were normalised against *gyrB* . Relevant strains are: WT, Wild‐type (DJ), *modE1* (CAA023), *modE2* (CAA022), *modE1 modE2* (CAA026). Two‐way ANOVA with Tukey's multiple comparisons was used to compare means, ****pvalue < .0001, *pvalue < .02. All other pairwise comparisons were non‐significant.

To understand the hierarchy of metal‐dependent regulation that enables the expression of vanadium nitrogenase, we examined the influence of molybdenum on the regulation of the *vnfUA1* operon that encodes the vanadium nitrogenase‐specific transcriptional regulator VnfA1. Most diazotrophs scavenge molybdenum from their environment using high‐affinity ABC transporters. *A. vinelandii* unusually contains three copies of *modABC* operons, that encode high‐affinity molybdate transporters. The most well characterised of these, the *modEA1B1C1* operon, encodes ABC components of a high‐affinity molybdate transport system that functions at external concentrations of >10 nM Mo and is regulated at the transcriptional level by the first gene in the operon, *modE* (Mouncey et al., 1996). When Mo is abundant, ModE tightly represses the expression of this transport system by binding to a motif within the promoter of this operon. Since expression of the alternative vanadium and iron‐only nitrogenases are repressed when Mo is available, ModE is an obvious candidate for Mo‐mediated repression of genes encoding the alternative nitrogenases, particularly as ModE binding sites are present in the promoters for *vnfA1* and *anfA*, the specific activators of the vanadium and iron‐only nitrogenases, respectively. However, it was shown previously that an insertion mutation in *modE1* does not entirely relieve Mo‐mediated repression of the *vnfUA1* and *anfA* promoters (Premakumar et al., 1998). Analysis of the genome sequence of *A. vinelandii* identified a gene adjacent to *vnfA2* designated *modE2*, encoding a protein with 76.7% identity to ModE1, which could function as an additional Mo‐responsive repressor. qRT‐PCR analysis of *vnfA1* transcription in wild‐type *A. vinelandii* showed that expression is repressed in the presence of molybdenum but not in the presence of vanadium or iron‐only conditions, as demonstrated previously (Figure 1). Analysis of single‐deletion mutations in either *modE1* or *modE2* demonstrated that repression of the *vnfUA* promoter is maintained in the presence of molybdenum when either of these genes is inactivated. However, repression was relieved in the double *modE1*, *modE2* deletion mutant (Figure 1). This implies that the two ModE repressors are functionally redundant for repression at the *vnfUA1* promoter, similar to the redundancy observed for multiple ModE homologues in photosynthetic bacteria (Demtröder et al., 2019b; Wiethaus et al., 2006).

### 2.2 The VnfA1 regulon of *A. vinelandii* includes transport operons and its paralogs, VnfA2 and VnfA3

Although VnfA1 is known to activate promoters required for the expression and maturation of vanadium nitrogenase, the number of genes that it regulates and hence the extent of the VnfA1 regulon is currently unknown. To determine the direct promoter targets of VnfA1, we carried out ChIP‐Seq analysis using a strain engineered to express *vnfA*1 with a C‐terminal 3X FLAG allele in the native location in the *A.vinelandii* genome. ChIP‐Seq data were obtained from cultures grown to mid‐exponential phase (to an OD_600_ of 0.4) either in the presence of vanadium or its absence (Fe‐only conditions). Under both of these conditions, VnfA1 is expressed (Figure 1) and expected to be in the active form. The FLAG tag did not hinder the ability of VnfA1 to activate the *vnfH* and *vnfDGK* promoters (known targets of VnfA1), as measured by qRT‐PCR analysis (Supplementary Figure S2a,b). To complement the ChIP‐Seq analysis, we carried out genome‐wide 5′end mapping of primary transcripts at single‐nucleotide resolution, using the Cappable‐seq methodology, which uses an enrichment strategy to capture the 5′ends of primary transcripts (Ettwiller et al., 2016). For comparative purposes, we isolated RNA from cultures grown in the presence of either Mo, V or iron‐only conditions to provide quantifiable tags of initiated transcripts under conditions appropriate for expression of the three nitrogenases encoded by *A. vinelandii*. Since the consensus sequence for sigma‐54‐dependent promoters is highly conserved, this enables genome‐wide mapping of promoters regulated by bacterial enhancer‐binding proteins, and in the case of VnfA1, correlates the location of binding sites determined by ChIP‐Seq with transcription start‐sites. Finally, to assess the function of VnfA1 binding sites and verify transcriptional regulation, we carried out the qRT‐PCR analysis of target genes, comparing the wild‐type strain with a strain carrying a *vnfA1* deletion.

**FIGURE 2:**
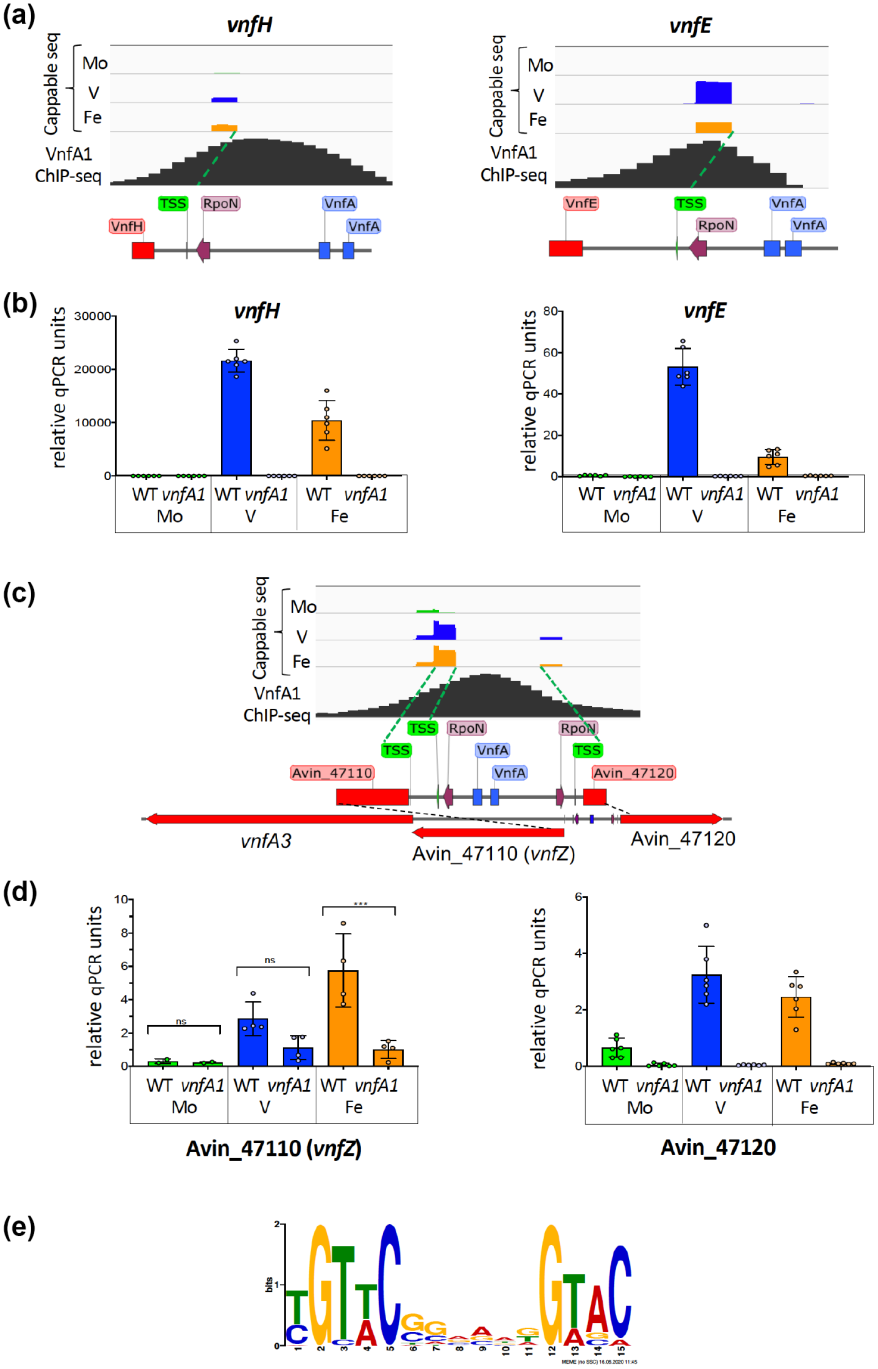
Examples of VnfA1 regulon characterisation, combining transcription start‐site (TSS) mapping with ChIP‐seq and qRT‐PCR analysis of target genes. (a) browser tracks of the *vnfH* and *vnfE* promoters. The top 3 tracks are Cappable‐seq reads of the wild‐type strain subjected to nitrogen stepdown in either Mo, V or Fe‐only conditions as indicated. The bottom track in each case shows the corresponding VnfA1 ChIP‐seq peak identified in strain CAA005 (VnfA1‐3FLAG) grown in the presence of V. a map of each promoter, shows TSSs, RpoN consensus sequences and VnfA1 sites identified by MEME analysis are shown beneath the tracks. (b) qRT‐PCR analysis of *vnfH* and *vnfE* in wild‐type (DJ) and *vnfA1* deletion (CAA013) strains, after nitrogen stepdown in either Mo, V or Fe‐only conditions. Relative qPCR units are defined as the ratio between the absolute levels of each gene ( *vnfH* or *vnfE* ) and the housekeeping gene *gyrB* . (c) Browser tracks and schematic map of the Avin_47110 ( *vnfZ* )‐Avin_47120 intergenic region presented as in a, but expanded to show the gene organisation of the *vnfZ‐vnfA3* operon. In this case, Cappable‐seq identified 2 TSSs for *vnfZ* , a relatively weak transcript present in all three growth conditions and a stronger RpoN ‐associated TSS that is activated in the absence of Mo. (d) qRT‐PCR analysis of *vnfZ* and Avin_47120 in wild‐type (DJ) and *vnfA1* deletion (CAA013) strains, after nitrogen stepdown in either Mo, V or Fe‐only conditions. Relative ANOVA analysis with Sidak's multiple comparisons was used to compare means. Ns indicates non‐significant, ***p value < .0002. E, DNA binding motif derived from MEME analysis of VnfA1 ChIP ‐seq targets (supplementary Table S5).

Although VnfA1 ChIP‐seq peaks were observed within genes, peaks with high *p‐*values were observed in intergenic regions upstream of sigma 54‐dependent promoters (Supplementary Table S8). We confirmed the currently known targets within the *vnf* gene cluster as *vnfH*, *vnfD* and *vnfE* (Supplementary Table S1). As expected from previous analysis, these operons are highly expressed in the presence of V (Hamilton et al., 2011), are dependent on VnfA1 for transcriptional activation (Walmsley et al., 1994; Woodley et al., 1996) and their transcription start sites were confirmed using Cappable seq (see Figure 2 for *vnfH* and *vnfE*). A VnfA1 ChIP‐seq peak was also observed in the intergenic region between Avin_02430 and Avin_02450 (Supplementary Figure S3). This contains divergent σ^54^‐dependent promoters, both of which are VnfA1‐dependent (Supplementary Table S1). Our qRT‐PCR analysis reveals that Avin_02430, which encodes a substrate‐binding protein typical of ABC‐type transport systems, forms part of a VnfA1‐dependent operon that includes Avin_02420 (nickel binding GTPase), Avin_02410 (ABC transporter, ATP‐binding domain), Avin_02400 (nitroreductase) and Avin_02390 (glutamate‐cysteine ligase) (Supplementary Figure S3). This operon is highly conserved amongst *Azotobacter* species and with the exception of the last gene, Avin_02390, is also conserved in representatives of the Proteobacteria that contain vanadium nitrogenases (Addo & Dos Santos, 2020), suggesting it performs an important function in supporting vanadium‐dependent nitrogen fixation. The divergently transcribed gene Avin_02450, which encodes an uncharacterised protein, is not as ubiquitous as this operon but is present in *Azotobacter* species and other representatives of the gamma and beta proteobacteria that contain vanadium nitrogenase genes.

The intergenic region between Avin_47110 and Avin_47120 also contains divergent sigma 54 ‐dependent promoters and a ChIP‐seq peak for VnfA1 (Figure 2). Transcriptional activation of Avin_47120, encoding an uncharacterised protein conserved in the gene neighbourhoods of other *Azotobacter* species, is *vnfA1* dependent. Transcriptional regulation of Avin_47110, which is annotated as a phosphonate binding protein, is more complex since Cappable‐seq reveals this gene is expressed from two promoters: a downstream promoter that is active in Mo, V and Fe‐only conditions and an upstream σ^54^ promoter that is activated in the absence of Mo and is partially dependent on the presence of *vnfA1*. qRT‐PCR analysis indicates that Avin_47110 is co‐regulated with the downstream gene Avin_47100, which encodes the VnfA paralog, VnfA3 (Figure 2). No internal promoters were detected in the Cappable‐seq analysis suggesting that *vnfA3* is located in an operon with Avin_47110. Moreover, the stop codon of Avin_47110 overlaps with the start codon of *vnfA3*, indicating that the expression of these genes is translationally coupled. VnfA1, therefore, influences the expression of VnfA3, by upregulating this operon from a σ^54^ promoter when Mo is absent. The Avin_47110‐VnfA3 operon arrangement is conserved in *Azotobacter* species raising the possibility that Avin_47110 might control the activity of VnfA3. From herewith, we will designate Avin_47110 as *vnfZ*. A similar complex promoter arrangement that regulates the expression of the second VnfA paralog, VnfA2 was also observed, with a constitutive downstream promoter and an upstream σ^54^ ‐dependent promoter that is *vnfA1*‐dependent and activated in the absence of Mo. (Supplementary Figure S4). Thus, the expression of the VnfA2 and VnfA3 paralogs is partially dependent on VnfA1.

### 2.3 Several genes are activated by VnfA1 only in the absence of Mo and V

**FIGURE 3:**
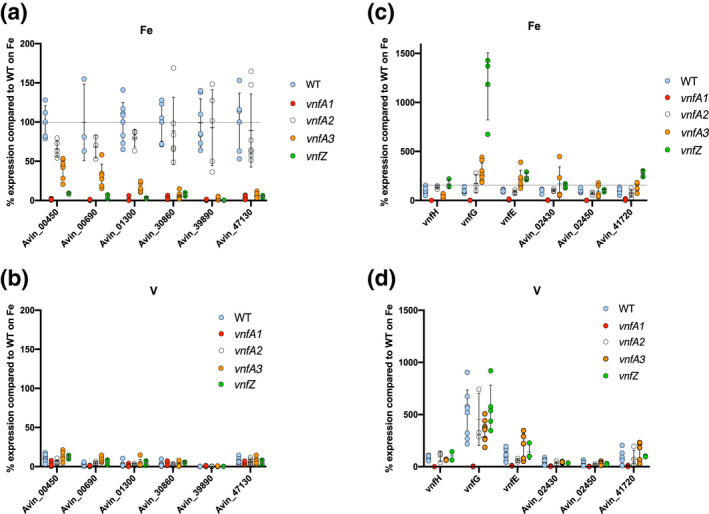
Differential requirements for activation of class B and class A promoters by VnfA paralogs in response to V and Fe‐only conditions. All panels show qRT‐PCR analysis of the genes indicated on the x axis, in wild type (WT) and deletion mutant strain backgrounds as indicated in the graph legends. Relevant strains are WT (DJ, blue dots), *vnfA1* (CAA013, red dots), *vnfA2* (CAA171, grey dots), *vnfA3* (CAA129, orange dots) and *vnfZ* (CAA206, green dots). Data for each gene (mean of at least 3 independent determinations) are normalised to 100% of the wild‐type value for that gene when strains are grown under Fe‐only conditions. (a) Analysis of class B promoters under Fe‐only conditions. (b) analysis of class B promoters in the presence of vanadium. (c and d) Analysis of class A promoters in Fe‐only and V conditions, respectively.

Amongst the ChIP‐seq targets bound by VnfA1, we identified several sigma 54 ‐dependent promoters, that are strongly activated under Fe‐only conditions but are activated only weakly when V is present (Supplementary Table S1, Figure 3). From herewith, we will refer to these promoters as Class B (activated only in the absence of Mo and V), in contrast to Class A targets that are activated in both V and Fe‐only conditions. All the Class B promoters require VnfA1 for transcriptional activation and they mostly regulate genes whose functions are potentially associated with metal ion acquisition. Three of these VnfA1‐dependent genes (Avin_30860, Avin_39890 and Avin_47130) encode TonB‐dependent siderophore receptors and are located in operons with putative ABC transport components. Curiously, the most downstream gene in one of these operons (Avin_39870) encodes a NifD‐like protein that does not influence growth in cultures grown diazotrophically in the presence of Mo (Setubal et al., 2009). Another target, Avin_00450, forms part of a putative iron ABC transporter operon. Unexpectedly, the *modA2, modB2, modC2* operon, which is not apparently regulated by ModE, is highly dependent on VnfA1 for upregulation from a σ^54^‐dependent promoter under iron‐only conditions (Supplementary Table S1), suggesting a specific role for this operon in molybdate acquisition under V‐limiting conditions. MEME analysis of VnfA1 ChIPseq targets identified a motif GT(T/A)C‐N6‐GTAC (Figure 2, Table S5), which is similar to the consensus DNA‐binding site identified by footprinting and deletion analysis of the *vnf* structural gene promoters (Woodley et al., 1996). A very similar motif was obtained when MEME analysis was limited to the Class B promoters activated by VnfA1 under Fe‐only conditions. Most promoters contained two motifs with similar spacing between them, in agreement with the suggestion that VnfA1 binds cooperatively to adjacent binding sites (Woodley et al., 1996) and that the protein purifies as a tetramer on size exclusion chromatography (Nakajima et al., 2010; Yoshimitsu et al., 2011).

### 2.4 Co‐involvement of VnfA paralogs in promoter regulation

Activation of gene expression by VnfA1 specifically under Fe‐only conditions at the Class B promoters implies that VnfA1 is not competent to activate transcription at these promoters when V is present. These observations suggest various scenarios whereby this pattern of regulation is achieved. We considered the possibility that these genes are required to support the activity of the iron‐only nitrogenase and that perhaps AnfA, the activator for the iron‐only system is required as a co‐activator in the absence of V. However, qRT‐PCR experiments revealed that the pattern of expression observed for Avin_30860 and Avin_39890 in the wild‐type strain did not alter significantly in an a*nfA* deletion strain (Supplementary Figure S5), indicating that AnfA is not required as a co‐activator. Since VnfA1 activates the expression of VnfA2 and VnfA3, we also considered the possibility that these paralogs could be required as co‐activators for promoters that are activated under Fe‐only conditions in the absence of V. In further qRT‐PCR analysis, we observed that three of the Class B promoters (Avin_00450, Avin_0069 and Avin_01300) were partially co‐dependent on *vnfA3* for activation under Fe‐only conditions, whereas the other three promoters (Avin_30860, Avin_39890 and Avin_47130) were more strongly co‐dependent on *vnfA3* (Figure 3a). In contrast, *vnfA2* appears to have no significant role in coactivating these promoters. These data, therefore, suggest that both VnfA1 and VnfA3, are required to co‐activate class B promoters under Fe‐only conditions. Clearly, these promoters are poorly expressed in the presence of V since transcript levels for each gene in the wild‐type strain were less than 20% of those observed in Fe‐only conditions and *vnfA2* and *vnfA3* mutations did not significantly affect this basal level of activity (Figure 3b).

### 2.5 VnfA3 binds to the same targets as VnfA1

In order to further explore the co‐activation requirements for Class B promoters under Fe‐only conditions, we focused on VnfA3, as some genes are strictly co‐dependent on this activator and homologues of VnfA3 are widespread amongst *Azotobacter* and related diazotrophic genera, in contrast to VnfA2 which is apparently confined to *A. vinelandii* (Supplementary Figure S6). ChIP‐Seq analysis was carried out using a strain engineered to express VnfA3 with a C‐terminal 3X FLAG allele from the native location in the *A. vinelandii* genome, using cultures grown in the presence of vanadium or Fe‐only conditions. The FLAG‐tagged *vnfA3* allele was competent to activate Class B promoters (Supplementary Figure S2, panels D and E). Surprisingly, we observed that VnfA3 is bound to exactly the same targets as VnfA1, including the *vnf* structural gene promoters, irrespective of the requirement for VnfA3 for co‐activation. Considerable overlap was observed between the VnfA1 and VnfA3 ChIP‐seq peaks (Supplementary Figure S7), which is perhaps not unexpected given that these activators possess highly similar DNA recognition helices (Supplementary Figure S1). In accordance with this, MEME analysis of targets filtered to discriminate high *p* values for VnfA3 (<3 e‐13, Supplementary Table S6) did not reveal significant differences in the motif previously identified as being the recognition sequence for VnfA1.

The co‐occupancy of VnfA1 and VnfA3 at all targets listed in Supplementary Table S1 prompted us to examine the influence of VnfA3 on the Class A promoters (Figure 3c,d). In contrast to the Class B promoters, transcriptional activation of Class A targets was not significantly influenced by deletion of *vnfA3*, although slight increases in transcript levels were observed for the *vnfG*, v*nfE* and Avin_02430 genes in Fe‐only conditions (Figure 3c). It is difficult to rationalise how the co‐binding of VnfA1 and VnfA3 to targets leads to the requirement for co‐activation by VnfA3 at only a subset of promoters. For some of the Class B promoters, ChIP‐seq log fold changes for both VnfA1 and VnfA3 increased in the presence of Fe compared with V (Supplementary Figure S8). This may imply that co‐activation at these promoters is facilitated by the formation of more stable nucleoprotein complexes containing VnfA1 and VnfA3 when V is limiting.

### 2.6 Involvement of VnfZ in vanadium regulation

As shown in Figure 2, *vnfZ* is co‐transcribed in an operon with *vnfA3*, and the two genes are translationally coupled, raising the possibility that VnfZ might interact with VnfA3 and regulate its activity. Notably, *vnfZ* is co‐located with *vnfA3* in the genomes of various *Azotobacter* species and related diazotrophic gammaproteobacteria that encode V nitrogenase (Supplementary Figure S9). VnfZ is a homologue of Avin_02550, a putative periplasmic substrate‐binding protein located in an operon with Avin_02540 and Avin_02560, which are annotated as components of a phosphonate transporter but are predicted to encode an ABC transporter for vanadate (Hamilton et al., 2011). These genes are now designated as *vodA*, *vodB* and *vodC*, respectively, to indicate they are paralogs of *mod* transport genes. However, unlike VodA, VnfZ lacks the corresponding N‐terminal periplasmic signal peptide, suggesting that this protein may be confined to the cytoplasm. The potential for VnfZ to modulate the activity of VnfA3 might, therefore, be dependent on its ability to bind vanadium

We constructed an in‐frame deletion of *vnfZ* to check the effect of this gene on promoter regulation. This deletion does not impair VnfA3 expression as judged by the activity of a *vnfA3‐lacZ* translational fusion introduced into the native location (Supplementary Figure S10). The *vnfZ* deletion prevented activation of all the Class B promoters under Fe‐only conditions suggesting that this gene plays a major role in enabling co‐activation by VnfA1 and VnfA3 under Fe‐only conditions (Figure 3 panel A). In contrast, *vnfZ* was not required for activation of Class A genes (Figure 3 panels C and D) most of which are expressed to similar levels in both V and Fe‐only conditions. A notable exception in this group is the *vnfDGK* operon, which is expressed approximately fivefold higher in V compared with Fe‐only conditions. Previous transcriptome analysis of this operon also detected significant repression under Fe‐only conditions (Hamilton et al., 2011; Pence et al., 2021). This makes physiological sense since the structural subunits of the V nitrogenase are not essential for growth in V‐limiting conditions when expression of the iron‐only nitrogenase is activated. Notably, the *vnfZ* deletion increased the level of *vnfG* transcripts in Fe‐only conditions to a level even higher than that observed in the presence of V (Figure 3 compares panels C and D). The role of VnfA3 and VnfZ in regulating *vnfDGK* expression in Fe‐only conditions is therefore completely opposite to that observed for the Class B promoters. In contrast, *vnfG* is apparently downregulated by the presence of VnfA3 and VnfZ in the absence of vanadium, suggesting that these components can negatively modulate the activation of the *vnfD* promoter under Fe‐only conditions. Notably, ChIP‐seq peak heights for VnfA1 and VnfA3 (Supplementary Figure S7) and their corresponding log fold changes (Supplementary Figure S8) increased under Fe‐ only conditions at the *vnfD* promoter, suggesting that in this case increased promoter occupancy may result in downregulation of this operon.

### 2.7 Interactions between VnfA paralogs and VnfZ

**FIGURE 4:**
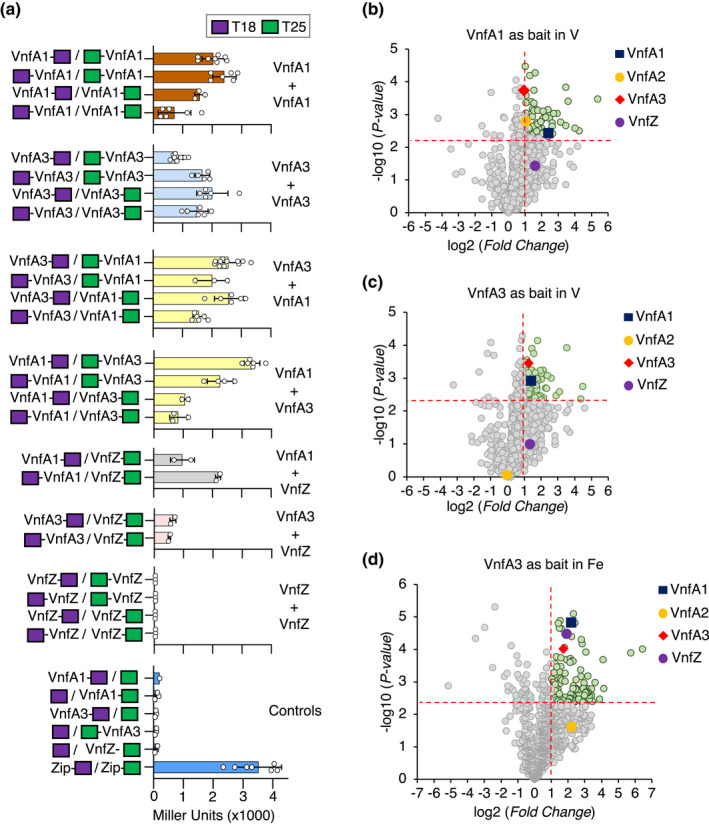
Protein–protein interactions between VnfA paralogs and VnfZ. (a) interactions between Vnf regulators reported by the bacterial two‐hybrid (BACTH) system in *Escherichia coli* BTH101 determined by β‐galactosidase activity. Interactions between VnfA paralogs were tested with all four combinations of fusion proteins. The locations of the T18 and T25 fragments of adenylate cyclase in the fusion proteins are indicated in violet and green rectangles respectively. Interactions between VnfA1 protomers are indicated in rust, VnfA3 protomers in light blue and VnfA1‐VnfA3 protomers in yellow. Interactions between VnfA1 and VnfA3 with VnfZ are indicated in grey and pink respectively. Blue bars indicate negative controls in which fusion proteins are combined with empty two‐hybrid vectors and a positive control using leucine zipper domain fusions proteins (Zip‐T25 + Zip‐T18). (b–d) In vivo co‐immunoprecipitation followed by mass spectrometry analysis of VnfA1‐FLAG in the presence of V (b) and VnfA3‐FLAG in the presence (c) or absence (Fe‐only conditions) of V (d). The immunoprecipitation assays were performed after in vivo cross‐linking using anti‐FLAG magnetic beads as described in the methods section. In each case, the non‐tagged wild‐type strain was used as a control for differential analysis of non‐specific interactions. Proteins eluted from the magnetic beads were analysed by mass spectrometry. Proteins identified from three independent biological replicates were used for differential enrichment analysis (FC > 2, *p* value < .005) and are presented in the volcano plots. The top 25 proteins detected by mass spectrometry are shown in supplementary Figure S12. The full data set is also available in supplementary Table S9.

To examine the potential for interaction between VnfA3 and VnfA1, we carried out the bacterial two‐hybrid analysis in *E. coli*, using fusions of these activators to the T18 and T25 adenylyl cyclase subunits, located either at their N or C‐termini. We observed self‐association of both VnfA1 and VnfA3 as expected and also evidence for the formation of hetero‐oligomers between these activators, with interaction being observed with all four fusion combinations (Figure 4a). We also examined interactions with VnfA2. Intriguingly, no self‐association was observed between VnfA2 protomers. However, whereas VnfA2 was apparently competent to interact with VnfA1, no interaction was detected with VnfA3 (Supplementary Figure S11). We further investigated interactions between the VnfA paralogs and VnfZ. As anticipated from the translational coupling of VnfZ with VnfA3 and the influence of *vnfZ* on the activity of the Class B promoters, the two‐hybrid analysis revealed that VnfZ interacts with VnfA3, but it also was observed to interact with VnfA1 (Figure 4a)

To support the two‐hybrid analysis, we performed co‐immunoprecipitation coupled to mass spectrometry (ColP‐MS/MS) in *A. vinelandii* using the 3xFLAG‐tagged variants of VnfA1 and VnfA3 proteins as baits. Cultures were grown exactly as described for the ChIP‐seq sample preparation in the presence or absence of V (Fe‐only conditions). Cultures were cross‐linked and protein extracts prepared for co‐immunoprecipitation as described in the methods. The VnfA1‐FLAG protein from cell lysates grown in the presence of V (Figure 4b) and the VnfA3‐FLAG protein from cell lysates grown in the presence or absence of V (Figure 4c,d) were recovered from lysates using magnetic beads conjugated to anti‐FLAG antibodies as described in the methods. Proteins retained on the beads were eluted and identified by mass spectrometry. To subtract proteins that bound non‐specifically to the anti‐FLAG antibody or the beads, we performed control experiments in parallel with the wild‐type *A. vinelandii* strain (DJ) encoding non‐tagged versions of VnfA1 and VnfA3. A comparison of the obtained peptide profiles revealed a significant enrichment of the bait proteins in the FLAG‐tagged samples compared to the control samples. In the presence of V, both VnfA2 and VnfA3 were enriched in the pull‐down fraction when VnfA1 was used as bait (Figure 4b), suggesting that VnfA1 interacts with both of its paralogs as suggested by the ChIP‐Seq data (in the case of the VnfA1 and VnfA3 interaction) and in line with the bacterial two‐hybrid analysis (Figure 4a). Conversely, in the presence of V, we observed that VnfA1, but not VnfA2, was enriched when VnfA3 was used as bait (Figure 4c). This suggests that VnfA1 and VnfA3 can interact in vivo (Figure 4a) and that VnfA3 is unable to interact with VnfA2 as also confirmed by the bacterial two‐hybrid analysis (Supplementary Figure S11). Finally, we also observed that in the absence of V (Fe‐only conditions), VnfA3 pulled down VnfA1 (Figure 4d) as observed when the cultures were grown in the presence of V (Figure 4c). Again, no evidence for interaction between VnfA3 and VnfA2 was observed. More importantly, under Fe‐only conditions, VnfZ was also pulled down with VnfA3 in line with our observations that VnfZ is required to support co‐activation of the Class B promoters by VnfA1 and VnfA3 (Figure 3).

### 2.8 VnfA1 represses *anfA* expression in the presence of vanadium

**FIGURE 5:**
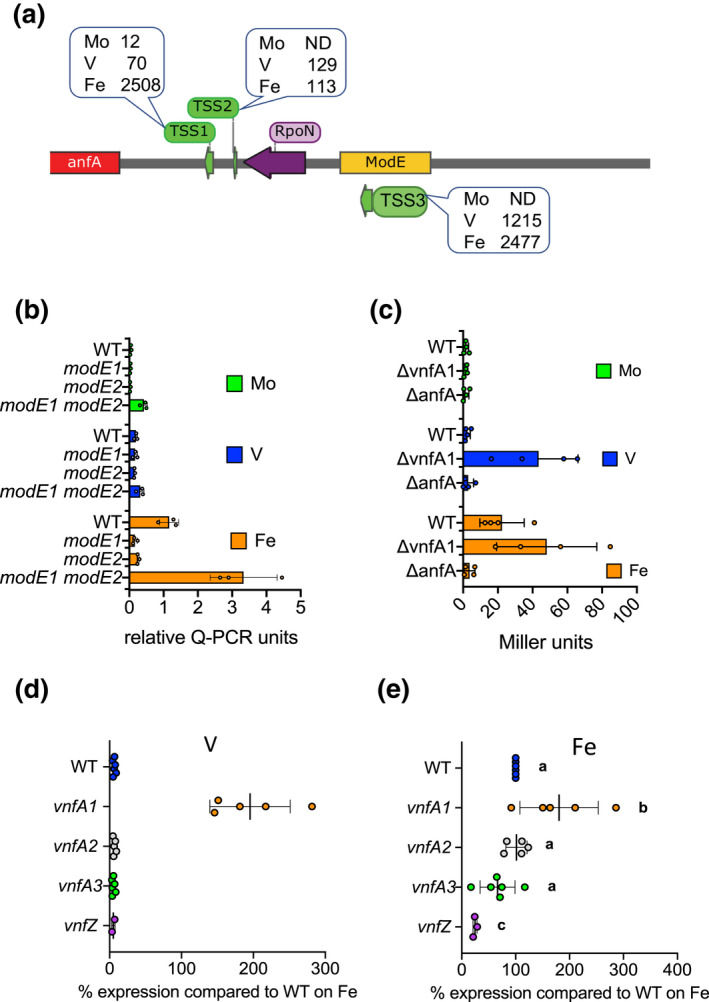
Metal‐dependent regulation of the *anfA* promoter. (a) Map of the promoter showing the location of the three TSSs identified by Cappable‐seq and the locations of the RpoN promoter and putative ModE binding site. TSS1 Is correctly positioned downstream of the −12 and −24 elements of the RpoN‐dependent promoter. The callouts with numbers above each TSS indicate the filtered reads observed in the wild‐type strain in either Mo, V or Fe‐only conditions. ND indicates not detected. (b) qRT‐PCR analysis of a*nfA* transcripts in the WT strain DJ and in response to *modE1* (strain CAA023), *modE2* (strain CAA022) and a double *modE1* , *modE2* deletion (strain CAA026), under Mo, V and Fe‐only conditions. Transcript levels were normalised against *gyrB* . (c) Influence of *vnfA1* and a*nfA* on expression as reported from an *anfA‐lacZ* fusion, located in the *algU* locus, a neutral site in the *Azotobacter vinelandii* genome. The relevant strains are the *anfA‐lacZ* fusion in the WT (CAA140), Δ*vnfA1* (CAA148) and Δ*anfA* (CAA144) backgrounds. (d and e) qRT‐PCR analysis of transcripts in the wild‐type and deletion mutant strains indicated on the x axis, normalised to 100% of the wild‐type value when grown under Fe‐only conditions. Relevant strains are: WT (DJ, blue dots), *vnfA1* (CAA013, yellow dots), *vnfA2* (CAA171, red dots), *vnfA3* (CAA129, green dots) *vnfZ* (CAA206, purple dots) and *anfA* (CAA030, violet dots). (d) Analysis of a*nfA* transcripts in strains grown in the presence of V. (e) analysis of *anfA* transcripts in strains grown under Fe‐only conditions. Different alphabetical characters above the plot indicate statistical differences as determined by ANOVA with post hoc Tukey's HSD.

Previous studies have revealed that expression of AnfA, the transcriptional activator of genes encoding the iron‐only nitrogenase, is repressed by both molybdenum and vanadium, in contrast to VnfA expression, which is only repressed by Mo (Premakumar et al., 1998). Cappable transcript mapping revealed that the *anfA* promoter is complex, containing three transcription start‐sites (TSS) (Figure 5a). TSS1 is correctly positioned downstream of consensus −12 and − 24 sequences delineating a σ^54^ (RpoN)‐dependent promoter, TSS2 is relatively weak and TSS3 is located within a putative ModE binding site. Quantitation of filtered reads revealed that all three transcripts are repressed by Mo and TSS1 is strongly repressed by V. To further investigate Mo regulation, we carried out the qRT‐PCR analysis of *anfA* transcripts in wild‐type and *modE1*and *modE2* mutants. In contrast, to the *vnfA1* promoter, repression of *anfA* transcription by Mo was not completely relieved in the double *modE1, modE2* mutant (compare Figure 5b with Figure 1), suggesting that additional factors are required to achieve optimal AnfA expression. Moreover, transcription was significantly repressed in the presence of V as previously reported (Premakumar et al., 1998) and as anticipated, the highest level of expression was observed under Fe‐only conditions (Figure 5b). To examine the potential role of σ^54^‐dependent activators in regulating *anfA* transcription from TSS1, we inserted an *anfA‐lacZ* fusion into a neutral site (*algU*) in the *A. vinelandii* genome. Deletion of *vnfA1* in this background, resulted in enhanced expression of *anfA* in the presence of V and Fe, but not in Mo, suggesting that VnfA1 is also a repressor of *anfA* transcription (Figure 5c). In contrast, deletion of *anfA* resulted in a strong decrease in promoter expression under Fe‐only conditions, implying that AnfA is required to autoactivate transcription from the RpoN‐dependent TSS1 promoter in the absence of Mo and V. To further investigate the role of VnfA paralogs in regulating *anfA* expression we also used qRT‐PCR to examine *anfA* transcripts under the same conditions (Figure 5d,e). In the presence of vanadium, the *anfA* promoter was silent in the wild type and all the *vnf* mutant strains tested, with the exception of the *vnfA1* deletion (Figure 5d), which confirms that VnfA1 represses *anfA* transcription in the presence of vanadium as observed with the *anfA‐lacZ* fusion in Figure 5c. Similar levels of expression were observed in the *vnfA1* deletion background under Fe‐only conditions and whereas *vnfA2* and *vnfA3* mutations had no significant influence on *anfA* transcripts in the absence of both Mo and V, the *vnfZ* deletion reduced *anfA* transcription to 25% (Figure 5e). Overall, these results suggest that a basal level of *anfA* transcription is provided by TSS2 and TSS3 in the absence of Mo, which is enhanced by autoactivation of TSS1 by AnfA under Fe‐only conditions. However, autoactivation by AnfA is apparently repressed by VnfA1 in the presence of V. VnfA1, therefore, plays an important role in curbing expression of AnfA, when V is available, which consequently prevents expression of the Fe‐only nitrogenase under these conditions. This repressor function of VnfA1 is also supported by previous data suggesting that *vnfA1* influences a*nfH* expression (Walmsley et al., 1994) and that Fe‐only nitrogenase component proteins are present in a *vnfA1* mutant strain when grown in the presence of V (Joerger et al., 1989). However, although the FLAG‐tagged allele of VnfA1 was competent to repress *anfA* transcription in the presence of V (Supplementary Figure S2, panel C), we could not detect a ChIP‐seq peak for VnfA1 at the *anfA* promoter suggesting that the repression exerted by VnfA1 in the presence of vanadium might be indirect. We also considered the possibility that VnfA1 might directly interact with AnfA to modulate its activity. In bacterial two‐hybrid analysis, AnfA interacted with VnfA1, but not with VnfA3. (Supplementary Figure S11). This specificity might reflect the unique ability of VnfA1 to exert a negative influence on *anf* transcription in the presence of V, by forming complexes with AnfA, a property that is not shared with VnfA3.

### 2.9 VnfA paralogs regulate the expression of Fe‐only nitrogenase

**FIGURE 6:**
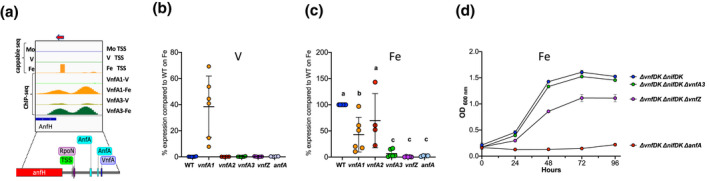
Influence of *vnf* regulatory genes on activation of the *anfH* promoter and Fe‐only nitrogenase dependent growth. (a) Browser tracks of the *anfH* regulatory region. The top three tracks are Cappable‐seq TSS reads from the wild‐type strain incubated under Mo, V and Fe‐only conditions as indicated. The four tracks below this show ChIP‐seq peaks for VnfA1 and VnfA3 from the wild‐type strain grown in the presence of V or Fe‐only conditions as indicated. The regulatory sequence is expanded below to indicate the location of the RpoN promoter, AnfA binding sites mapped by footprinting (Austin & Lambert, 1994) and a putative VnfA binding site detected by MEME analysis. (b and c) qRT‐PCR analysis of *anfH* transcripts in the wild‐type and deletion mutant strains indicated on the x axis, normalised to 100% of the wild‐type value when grown under Fe‐only conditions. Relevant strains are: WT (DJ, blue dots), *vnfA1* (CAA013, yellow dots), *vnfA2* (CAA171, red dots), *vnfA3* (CAA129, green dots) *vnfZ* (CAA206, purple dots) and *anfA* (CAA030, violet dots). (b) Analysis of *anfH* transcripts in strains grown in the presence of V. (c) Analysis of an*fH* transcripts in strains grown under Fe‐only conditions. Different alphabetical characters above the plot indicate statistical differences as determined by ANOVA with post hoc Tukey's HSD. (d) Influence of *vnf* regulatory genes on diazotrophic growth dependent on the Fe nitrogenase in strains lacking structural genes for the Mo and V nitrogenases. Cultures were grown in triplicate under Fe‐only conditions with excess ammonium, then centrifuged, resuspended in nitrogen‐free medium and then diluted to an initial OD_600_ _nm_ of 0.2. Relevant strains are: *ΔvnfDGK, ΔnifDGK* (CAA298, blue dots); *ΔvnfDGK, ΔnifDGK, ΔvnfA3* (CAA299, green dots); *ΔvnfDGK, ΔnifDGK, ΔvnfZ* (CAA293, purple dots) and *ΔvnfDGK, ΔnifDGK, ΔanfA* (CAA292, red dots). Many of the data points overlap, so standard deviations are often not visible.

The transcriptional activator for iron‐only nitrogenase, AnfA, activates transcription of the *anf* structural operon in the absence of Mo and V by binding to two enhancer sites located upstream of the RpoN‐dependent *anfH* promoter (Austin & Lambert, 1994) (Figure 6a). Surprisingly, in our ChIP‐seq analysis, we observed that this promoter is also a target for binding VnfA1 and VnfA3 under Fe‐only conditions. Two distinct ChIP‐seq peaks were distinguished, the promoter distal peak overlapping with a VnfA site identified by MEME analysis and the promoter‐proximal peak overlapping with the RpoN promoter, perhaps indicative of interaction between VnfA paralogs and sigma 54‐RNA polymerase (Figure 6a). To investigate the potential role of Vnf regulators in controlling transcription of the *anfHDGK* operon, we carried out qRT‐PCR analysis with relevant deletion strains to compare transcript levels of *anfG* with the wild‐type strain. As anticipated from the role of VnfA1 in repressing expression of *anfA*, no *anfG* transcripts were detectable in the presence of V, with the exception of the *vnfA1* deletion strain (Figure 6b). These results suggest that vanadium does not directly regulate the activity of AnfA, which is apparently competent to at least partially activate *anfHDGK* transcription in the absence of VnfA1. Under Fe‐only conditions, the *vnfA1* mutant reduced transcript levels to around 40% of the wild‐type, which implies that VnfA1 might play a role in supporting activation of the *anfH* promoter by AnfA under these conditions (Figure 6c). Whereas *vnfA2* had no significant influence on transcript levels, the *vnfA3* mutant reduced transcripts to 6% of the wild‐type, implicating a role for VnfA3 in the co‐activating transcription of the promoter with AnfA under Fe‐only conditions. As expected, only low levels of transcripts were detectable in the *anfA* mutant and the *vnfZ* mutation also abrogated transcription (Figure 6c). Overall, the direct binding of VnfA1 and VnfA3 to the *anfH* promoter observed by ChIP‐seq, in combination with the transcript analysis, suggests that these paralogs co‐activate transcription of the *anf* structural genes together with AnfA under Fe‐only conditions. The ChIP‐seq data indicate that the intensity of the peaks is strongly enhanced in the absence of V, suggesting a mechanism for promoting co‐activation by VnfA paralogs when V is unavailable. These results also suggest that VnfZ plays a major role in enabling the co‐activation, perhaps by controlling the activity of the paralogs in response to metal availability. The *anfH* promoter, therefore, has similar properties to the VnfA paralog‐dependent Class B promoters, with the added complexity of the stringent requirement for AnfA.

The above results reveal that the *vnf* regulatory genes play an important role in controlling the expression of the *anf* genes and are, therefore, likely to influence growth supported by the Fe‐only nitrogenase. To investigate this, we constructed strains with deletions in both Mo and V structural genes (Δ*nifDK*, Δ*vnfDGK*) to ensure that growth was entirely dependent on the Fe‐only nitrogenase under nitrogen‐deficient conditions and introduced further *vnfA3*, *vnfZ* and *anfA* deletions into this strain background. Strains were grown in Fe‐only medium in the presence of excess ammonium, then washed and resuspended in an N‐free medium to examine growth phenotypes. As expected, the *anfA* deletion, prevented growth under these conditions (Figure 6d). Surprisingly, the *vnfA3* deletion had no apparent impact on the growth rate, perhaps reflecting some level of functional redundancy of the VnfA paralogs. However, the *vnfZ* deletion reduced the exponential growth rate by 50% compared with the Δ*nifDK*, Δ*vnfDGK* strain (0.02 h^−1^ cf. 0.04 h^−1^) and also decreased the total accumulation of biomass (Figure 6d), conversant with the impact of *vnfZ* on *anf* transcription and the ability of VnfZ to interact with both VnfA1 and VnfA3.

## 3 DISCUSSION

**FIGURE 7:**
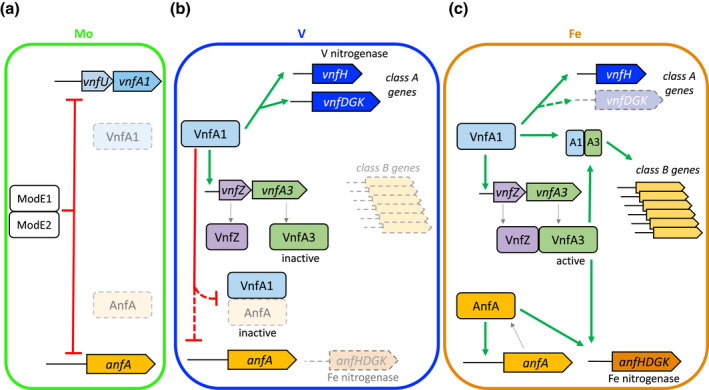
Model for hierarchical regulation of nitrogenase isoenzyme expression in response to metal availability. (a) In Mo replete conditions, Mo‐dependent nitrogenase is expressed and the Mo regulatory proteins ModE1 and ModE2 directly repress the transcription of the *vnfUvnfA1* and *anfA* operons, thus preventing expression of the alternative nitrogenases. (b) In the absence of Mo and presence of V, VnfA1 is expressed and represses transcription of *anfA* . Since we have not detected the binding of VnfA1 to the *anfA* promoter, this repression may be indirect and may involve an interaction between VnfA1 and AnfA that prevents auto‐activation of *anfA* expression. Under these conditions, VnfA1 activates transcription of the class A genes including the V nitrogenase structural genes *vnfH* and *vnfDGK*. VnfA1 also activates transcription of the *vnfZ vnfA3* operon, but VnfA3 is apparently inactive in the presence of V, presumably because it does not interact with VnfZ. Consequently, class B genes are not activated in the presence of V. (c) Under Fe‐only conditions (when neither Mo or V are available), VnfA1 no longer represses *anfA* transcription and VnfZ interacts with VnfA3, enabling co‐activation of class B gene promoters by VnfA1 and VnfA3. Under these conditions expression of V‐dependent nitrogenase decreases, potentially as a consequence of VnfA1‐VnfA3 interactions that repress activation of *vnfDGK* transcription. In the absence of V, VnfZ and VnfA3 also co‐activated *anfHDGK* transcription with AnfA resulting in the expression of the Fe‐only nitrogen.

Although it is well established that expression of the three nitrogenase isoenzymes in *A. vinelandii* is regulated at the transcriptional level by three discrete bEBP homologues, NifA, VnfA1 and AnfA, the mechanisms that maintain the hierarchy of metal‐dependent regulation of the three nitrogenases are not well understood. Clearly, molybdenum regulates the expression of the alternative nitrogenase activators VnfA1 and AnfA, ensuring that the V and Fe‐only nitrogenases are not synthesised when Mo nitrogenase is active. We have refined this model by demonstrating that Mo repression of *vnfA1* and *anfA* transcription is mediated by two ModE paralogs, that are functionally redundant at these promoters as observed for ModE‐like regulators in other diazotrophs (Demtröder et al., 2019a). In contrast to the relative simplicity of Mo‐dependent regulation (Figure 7a), we have uncovered unprecedented complexity in the regulation of alternative nitrogenases in response to vanadium. Our data not only reveal the importance of VnfA1 in gene regulation but also identify the roles of the paralogous bEBP, VnfA3, in fine‐tuning alternative nitrogenase gene expression in response to metal acquisition. A regulatory model summarising most of our findings is shown in Figure 7.

Given that VnfA1 is the master transcriptional regulator for vanadium‐dependent nitrogen fixation, it is perhaps not surprising that the *vnfA1* regulon extends outside the structural and maturation genes required for vanadium nitrogenase biosynthesis, including its paralogs *vnfA2* and *vnfA3* whose expression is upregulated by VnfA1. However, the stringent requirement for VnfA1 to activate promoters that are only expressed in the absence of Mo and V (Fe‐only conditions) is unexpected and reveals a role for VnfA1 and its paralogs in activating gene expression under conditions of V‐limitation. As anticipated from the similarity of their DNA binding recognition helices, our ChIP‐Seq data demonstrate that VnfA1 and VnfA3 bind to the same target sigma‐54 ‐dependent promoters. Since activation of bEBPs is generally dependent on the assembly of hexameric rings and both VnfA3 and VnfA2 interact with VnfA1, it is likely that transcriptional activation can be modulated by the formation of hetero‐hexamers. Although co‐binding of VnfA1 and VnfA3 was observed in all target promoters in the presence of vanadium, VnfA3 had relatively little influence on transcriptional regulation of the Class A promoters that are activated either in V replete or V‐limiting (Fe‐only) conditions. In contrast, at the Class B promoters (expressed in Fe‐only conditions), which are stringently VnfA1‐dependent and yet are repressed in the presence of vanadium, transcriptional activation was enhanced by the presence of VnfA3, potentially driven by the formation of VnfA1‐VnfA3 hetero‐oligomers (Figure 7, compare panels B and C). The extent of paralog co‐dependence is variable amongst this class, but three of these promoters stringently require both VnfA1 and VnfA3 to activate transcription. At these three promoters, we observe lower *p*‐values and higher log‐fold changes in the ChIP‐seq data for both VnfA1 and VnfA3 under Fe‐only conditions, suggesting that, in this case, DNA binding by these paralogs may be regulated by metal availability.

The *vnfZ* gene, which is co‐transcribed and translationally coupled to *vnfA3* appears to play a major role in controlling VnfA3 activity since transcriptional activation of Class B promoters is ablated in the absence of VnfZ. Our co‐IP studies in *A. vinelandii* demonstrate that VnfZ interacts with VnfA3 in Fe‐only conditions, suggesting that VnfZ activates VnfA3 in the absence of V. Since VnfZ is a paralog of VodA, the substrate‐binding component of a putative ABC transporter for vanadate, it is possible that VnfZ is inactivated when bound to V and dissociates from VnfA3 under these conditions, since we do not observe an enrichment of VnfZ in co‐IP experiments when strains are grown in the presence of V. In line with our finding that VnfA3 is not required to activate the Class A promoters, VnfZ had little influence on the regulation of promoters that are activated in the presence of vanadium (Figure 7b). However, the expression of the *vnfDGK* operon, encoding the VFe component of vanadium nitrogenase, is a notable exception. We observe that the expression of this operon is downregulated in Fe‐only conditions and VnfZ is required to maintain this repression in the absence of V (Figure 7c). Perhaps, VnfZ sets up an interaction between VnfA1 and VnfA3 under Fe‐only conditions that disfavours activation of this promoter, in contrast to the Class B promoters, where the concerted action of these paralogous bEBPs stimulates transcriptional activation. This implies that promoter architecture plays a significant role in configuring the mode of regulation by the paralogous activators.

The involvement of VnfA paralogs in regulating the expression of the Fe‐only nitrogenase provides additional checkpoints in the regulatory hierarchy to ensure that this isoenzyme is only expressed when Mo and V are unavailable (Figure 7). Control of a*nf* gene expression is exerted at two levels. Firstly, VnfA1 negatively regulates transcription of *anfA* in the presence of vanadium, a property that appears to be exclusive to VnfA1, as it is not observed with either VnfA2 or VnfA3. Although this regulation might be indirect since we do not detect a VnfA1 ChIP‐seq peak in the *anfA* promoter region, it is possible that this reflects direct interaction between VnfA1 and AnfA, as detected in our bacterial two‐hybrid analysis. Such an interaction could potentially inhibit AnfA activity in the presence of vanadium, thus preventing autoactivation of *anfA* expression from the σ^54^‐dependent *anfA* promoter (Figure 7b). Although the mechanism by which VnfA1 senses V is currently unknown, it is possible that the vanadium status is conveyed to VnfA1 by the V nitrogenase structural components since deletion of *vnfDGK* enables the expression of the *anfHDGK* operon in the presence of V (Luque & Pau, 1991)

The second mode of control of Fe‐only nitrogenase expression by the VnfA paralogs is exerted at the *anfH* promoter, in which binding of both VnfA1 and VnfA3 to the promoter increases under Fe‐only conditions. Our results suggest a role for VnfA1‐VnfA3 in coactivating the promoter with AnfA (Figure 7c). The dependency on VnfZ is indicative of co‐activation in the absence of V as observed with the Class B promoters. However, the mechanism of co‐activation is likely to be different since we and others have observed that AnfA and VnfA1‐VnfA3 have discrete binding sites in the promoter (Austin & Lambert, 1994; Frise et al., 1994). Although activation of the *anfH* promoter by AnfA alone was detected when these *A. vinelandii* components were expressed in the heterologous host *Klebsiella pneumoniae* (Frise et al., 1994), it is possible that activation, in this case, was a consequence of overexpression of these activators. Likewise, activation of the a*nfH* promoter by a truncated form of AnfA, lacking the N‐terminal GAF domain has been demonstrated in vitro Austin, (Austin & Lambert, 1994), although it is not clear whether this reflects the optimal level of transcription required for expression of the Fe‐only nitrogenase in vivo. Although under our standard growth conditions, *vnfA3* did not influence the ability of *A. vinelandii* to grow on dinitrogen using the Fe‐only nitrogenase, the *vnfZ* deletion had a significant influence on the growth rate, perhaps reflecting the involvement of VnfZ in controlling both VnfA1 and VnfA3 activity. Intriguingly, a recent study based on Tn‐Seq to analyse gene fitness in *A. vinelandii*, revealed that insertion mutations in *vnfA3* exhibited a fitness penalty when grown in the presence of Mo, either in the absence or presence of fixed nitrogen (Knutson et al., 2021). However, in our hands, we do not observe any obvious growth defects when *vnfA3* deletion strains are cultured on media containing molybdenum.

Although interactions between bEBP homologues have been rarely reported in the literature, a notable precedent for our findings is provided by the HrpR and HrpS proteins, which interact as hetero‐hexamers to activate the RpoN‐dependent *hrpL* promoter, conferring Type 3 secretion‐mediated pathogenicity in *Pseudomonas syringae* (Hutcheson et al., 2001; Jovanovic et al., 2011). The evolutionary advantages of HrpRS co‐dependence are unclear, although residues that influence nucleotide‐dependent oligomerisation in the AAA+ domains of these bEBPs are likely to fulfil an important mechanistic role (Lawton et al., 2014) and the formation of hetero‐oligomers may confer advantages with respect to regulation by the negative regulator HrpV (Preston et al., 1998), which only interacts with HrpS (Jovanovic et al., 2011). There are potential parallels here with the regulation of VnfA1 and VnfA3 by VnfZ since the interaction of HrpV with HrpS has been suggested to play an essential role in the assembly of the HrpRS complex (Jovanovic et al., 2014). It is possible that VnfZ might perform a similar role in promoting the formation of heteromeric VnfA1‐VnfA3 complexes, in line with the observation that VnfZ potentially interacts with both VnfA1 and VnfA3 as revealed by our bacterial two‐hybrid analysis. Further rare examples of hetero‐oligomeric interactions between bEBPs include FleQ and FleT, which act in concert to activate σ^54^ ‐dependent promoters controlling flagella gene expression in *Rhodobacter sphaeroides* (Peña‐Sánchez et al., 2009; Poggio et al., 2005) and the co‐dependent RedR1 and RedR2 bEBPs that activate expression of the resorcinol degradation pathway in *Azoarcus anaerobius* (Pacheco‐Sánchez et al., 2017).

Notably both the co‐dependent *P. syringae* HrpR‐HrpS and *R. sphaeroides* FleQ‐FleT bEBPs, lack N‐ terminal regulatory domains, so regulation of their interactions are limited to control of complex assembly, nucleotide‐dependent subunit interactions in the AAA+ domain and DNA binding affinity. In contrast, the presence of N‐terminal GAF domains in the three VnfA paralogs suggests multiple scenarios for regulating their activities including influences on oligomerisation, catalysis and interaction with σ^54^ RNA polymerase, as observed in other bEBPs with regulatory domains (Batchelor et al., 2013; Bush & Dixon, 2012; Gao et al., 2020; Shingler, 2011). However, the regulatory functions of the GAF domains in VnfA paralogs are currently enigmatic. The presence of conserved cysteine residues in the N‐terminus of these proteins is indicative of metal‐binding or ligation of a metal cluster. Although anaerobic reconstitution of recombinant VnfA1 expressed in *E. coli* suggests the GAF domain may contain a [Fe_3_‐S_4_] cluster, this domain does not appear to play a major role in regulating VnfA1 activity in *E. coli*, except when cultures are treated with a strong oxidising agent (Nakajima et al., 2010; Yoshimitsu et al., 2011). Since *vnfA1* is co‐expressed in *A. vinelandii* in an operon with v*nfU*, encoding a protein that exhibits homology to the C‐terminal domain of NifU and is likely to play a role in cluster maturation, it is possible that not all components required for assembly of the metal cluster in the VnfA1 GAF domain are present in *E. coli*. This is also pertinent to our use of the bacterial two‐hybrid system in *E. coli*, whereby cluster deficient GAF domains in bEBPs may perturb protein–protein interactions or, for example, prevent exploration of the regulatory role of V in promoting the formation or dissociation of protein complexes. This emphasises the importance of using the native organism to confirm the physiological relevance of complex formation and in the case of our Co‐IP analysis to demonstrate vanadium regulation of VnfZ‐VnfA3 interactions in *A.vinelandii*

Whatever sensory functions and signal perception mechanisms are employed by the GAF domains of VnfA paralogs, the evolution of non‐identical regulatory domains suggests the potential for elegant fine‐tuning of alternative nitrogenase expression and mechanisms for integrating signals within complexes formed by the interacting partners. Co‐dependent interactions between VnfA paralogs resulting in the formation of heterohexameric rings may enable further fine‐tuning of catalytic activity as a consequence of subunit specialisation in the AAA+ domain. This may also provide a mechanism for negative regulation consequent upon hetero oligomer formation, not only amongst the VnfA paralogs but also for the interactions detected between the nitrogenase master regulators VnfA1 and AnfA. Overall, our findings reveal an unprecedented level of interaction between bEBPs in a single organism, resulting in a highly sophisticated hierarchy of metal‐dependent regulation of alternative nitrogenases. This remarkable capacity to fine‐tune gene expression in response to metal availability may reflect the need to prevent wasteful energy utilisation by multiple isoenzymes and the incorporation of non‐cognate active site cofactors into nitrogenases, with consequences for altered substrate reduction (Gollan et al., 1993; Pau et al., 1993; Perez‐Gonzalez et al., 2022; Yang et al., 2014)

## 4 EXPERIMENTAL PROCEDURES

### 4.1 Strains and growth conditions

The bacterial strains used in this study are listed in Table S2. For routine procedures, *E. coli* strains were grown at 37°C in LB medium (Sambrook et al., 1989) and *A. vinelandii* was cultured at 30°C in a modified NIL medium (Dos Santos, 2011; Martinez‐Argudo et al., 2004), which contains 0.2 g/L MgCl_2,_ 90 mg/L CaCl_2,_ 0.8 g/L KH_2_PO_4,_ 0.2 g/L K_2_HPO_4_, 14 mg/L Na_2_SO_4_, 120 mg/L Fe_2_(SO4)_3_, supplemented with 2% sucrose. For all experiments in a liquid medium, apart from routine maintenance or genetic manipulation of strains, *A. vinelandii* was grown at 250 rpm in a NIL medium that had been filtered through activated charcoal to remove trace amounts of molybdenum (to <8 ppb) as described by (Schneider et al., 1991). Growth in charcoal‐filtered NIL medium, which contains 30 μM iron, is referred to as “Fe‐only conditions”. When required, this medium was supplemented with either Mo (1 μM) or V (1 μM) as indicated. For growth in nitrogen‐excess conditions, the medium contained 25 mM ammonium acetate. Antibiotics were used as follows: carbenicillin 50 μg/ml (*E. coli*), chloramphenicol 15 μg/ml (*E. coli*), tetracycline 5 μg/ml (*E. coli* and *A. vinelandii*), kanamycin 50 μg/ml (*E. coli*) and 3 μg/ml (*A. vinelandii*), streptomycin 2 μg/ml (*A. vinelandii*), gentamycin 0.1 2 μg/ml (*A. vinelandii*) trimethoprim 100 μg/ml (*A. vinelandii)*.

### 4.2 Recombinant DNA work

General molecular biology techniques were performed according to established protocols (Sambrook et al., 1989). Enzymatic isothermal assembly (Gibson et al., 2009) was performed with the NEBuilder HiFi DNA Assembly Master Mix (NEB #E2621). Site‐direct mutagenesis by overlapping PCR was performed as described previously (Urban et al., 1997). High‐fidelity DNA polymerase and restriction enzymes were provided by New England Biolabs. DNA purification was performed using commercially available kits provided by Macherey‐Nagel. Sanger DNA sequencing and oligonucleotide synthesis was conducted by Eurofins MWG Operon.

### 4.3 Genetic manipulation of *A. vinelandii*

*A. vinelandii*, mutants were generated either by transformation or conjugation as indicated in Table S2. The transformation was carried out essentially as described previously (Bueno Batista et al., 2021; Dos Santos, 2011, 2019). Indirect selection of recombinants was achieved by the congression procedure (Brigle et al., 1987; Robinson et al., 1986) in which co‐transformation with plasmid pDB303 harbouring the mutation *rpoB113* conferring rifampicin resistance is used for the initial selection of transformants. Alternatively, conjugation using the sucrose counter‐selection method (Schafer et al., 1994) was used to introduce mutations. Briefly, *A. vinelandii* DJ and derivatives of *E. coli* strain S17‐1 (harbouring the mobilizable plasmid pK18mobsacb carrying the DNA manipulation of interest) were grown to early exponential phase, mixed in a ratio of 100:5 and incubated for 24 h on an agar plate containing 90% NIL medium (with glucose instead of sucrose) and 10% LB. The conjugation mixture was then scraped from the plate, re‐suspended in 1 ml of 10‐fold diluted P buffer (4.6 mM K_2_HPO_4_,1.5 mM KH_2_PO_4_), and plated on NIL glucose plates with 5 μg kanamycin and 50 μg chloramphenicol (for *E. coli* counter‐selection). After 5 to 7 days, the kanamycin‐resistant colonies (containing the integrated plasmid) were patched on NIL media with sucrose to select for double cross‐over events. Given that *A. vinelandii* can accumulate multiple copies of its chromosome, newly recombinant colonies were exhaustively streaked on selective media (10 times or more) to ensure efficient chromosome segregation and homogeneity of mutant genotypes. Deletions were verified by the absence or amplification by PCR using primers inside the targeted gene.

### 4.4 Construction of 3x‐FLAG alleles of vnfA1 and vnfA3

The *A. vinelandii* strains expressing forms of VnfA1 (CAA005) or VnfA3 (CAA025) with a C‐terminal triple‐FLAG tag fusion (DYKDHDGDYKDHDIDYKDDDDK) were created by a two‐step fusion‐PCR approach. For both constructs, *A. vinelandii* purified genomic DNA was used as a template for two separate PCR‐reaction. In the first step, the reaction amplified the promoter and coding regions of *vnfA1 or vnfA3* using the primer pairs 15/17 and 192/196, respectively. The second reaction amplified an 800 bp sequence at the 3‘end of their coding regions using the primer pairs 16/18 and 194/197, respectively, in order to introduce a 24 bp overlapping sequence encoding the triple‐FLAG tag. In the final step, a PCR reaction using the primers 25/26 and 193/195 was used to amplify the entire *vnfA1* or *vnfA3* genes and their promoters, fusing the two products from step 1 together and incorporating the 3xFLAG tag sequence between them. Both PCR products were inserted in the pJet12Blunt vector using the manufacturer's recommendations (CloneJET PCR Cloning Kit, ThermoFisher). The resulting vectors were named pUCvnfA1::3FLAG and pUCvnfA3::3FLAG respectively. Both plasmids were introduced into *A. vinelandii* DJ by congression. The resulting strains CAA005 and CAA025 were used in the ChIP‐seq experiments described in this study.

### 4.5 Construction and assay of lacZ fusions in *A. vinelandi*

A transcriptional fusion of the *anfA* promoter with *lacZ* was inserted in a neutral position in the *A. vinelandii* genome (the *algU* gene is interrupted by an IS transposase in strain DJ of *A. vinelandii*). To construct this, we used a 6‐fragment isothermal assembly (NEBuilder® HiFi DNA Assembly, NEB) with the following PCR fragments: the backbone of the pUC19 plasmid (amplified with primers 363 and 368), 800 bp upstream and the 5″ end of the *algU* gene (amplified with primers 813 and 814), 357 bp upstream of the *anfA* ORF (amplified with primers 815 and 816), the full *lacZ* encoding sequence of *E. coli K*‐12 MG1566 (amplified with primers 817 and 818), the trimethoprim resistance gene *dhfR*II from the plasmid pTJ1 (amplified with primers 819 and 820) and 800 bp downstream of the IS insertion site starting in the *algU* ORF (amplified with primers 363 and 368). The assembled plasmid was designated as pUC*anfA::lacZ*. A 5.6 kbp fragment containing all the fragments excluding the plasmid backbone was then amplified by PCR with the primers 813 and 822 and transferred by transformation to the *A. vinelandii* DJ, Δ*anfA* and Δ*vnfA1* backgrounds. The recombinants were selected with trimethoprim resistance. The resulting strains are designated as CAA140, CAA144 and CAA148, respectively.

An *A. vinelandii* strain harbouring a protein fusion between VnfA3 and LacZ was constructed as follows: the N‐terminal portion of VnfA3 up to codon 253 was amplified with primers 777 and 778 and fused by isothermal recombination with *lacZ* from codon 8 (amplified with primers 779 and 780) and the 800 bp fragment downstream of *vnfA3* (amplified with primers 781 and 782) in the pKTmob‐sac vector. The resulting plasmid, pKTmobvnfA3::lacZ, was conjugated with *A. vinelandii* WT and Δ*vnfZ* (CAA206), resulting in strains CAA270 and CAA271, respectively.

To measure the level of expression of *A. vinelandii lacZ* fusions, cells were grown in the presence of ammonium acetate (25 mM) in metal depleted media for 2 days then washed and inoculated into ammonium‐free NIL medium with the addition of either Mo (1 μM), V (1 μM) or no addition (Fe‐only conditions, 30 μM Fe) as indicated. The cells were incubated for 24 h and β‐galactosidase activity assays were performed using 1 ml of culture as described previously (Miller, 1972) and reported in Miller Units.

### 4.6 Bacterial two‐hybrid assays

Bacterial adenylate cyclase two‐hybrid assays (BACTH) were performed as described previously (Karimova et al., 1998) using vectors and strains supplied by Euromedex, Souffelweyersheim, France). Briefly, the DNA fragments that encode the full‐length VnfA1, VnfA2, VnfA3, AnfA and VnfZ proteins were cloned into BACTH T18 and T25 containing vectors (primers listed in Supplementary Table S4, plasmids listed in Supplementary Table S3). The original empty vectors and the pUT18C‐zip/ pKNT25‐zip fusion combination were used as negative and positive controls, respectively. Plasmids were co‐transformed by electroporation into *E. coli* strain BTH101 and selected on LB medium containing 100 mg/ml carbenicillin and 50 mg/ml kanamycin. Single colonies were picked and grown for 16 h in 10 ml of LB with both antibiotics. β‐galactosidase activity assays were performed using 100 μl of culture as described previously (Miller, 1972) and reported in Miller Units.

### 4.7 RNA purification and qRT‐PCR

To minimise the effects of growth differences between strains we carried out short‐term de‐repression experiments in which *A. vinelandii* strains were grown under conditions of nitrogen excess and subsequently subjected to nitrogen step down as described previously (Poza‐Carrión et al., 2014). Briefly, cells growing under ammonium excess (NIL medium with ammonium acetate 25 mM) were collected by centrifugation, resuspended to an O.D_600_ of 0.5 in NIL medium, either with no addition (Fe‐only conditions) or supplemented with either Mo (1 μM) or V (1 μM) and subsequently incubated for 6 h prior to RNA extraction. To ensure the preservation of intracellular RNA, the cultures were immediately mixed with 1/5 of stop solution (5% Phenol saturated with 0.1 M citrate pH 4.3, 95% ethanol) (Bernstein et al., 2002) and then rapidly chilled on ice for 20 min. RNA was purified using the TRI Reagent (Sigma #T9424) following manufacturer instructions. Genomic DNA was removed by three treatments with the TURBO DNA‐free DNAse (Ambion #AM1907) according to the manufacturer's instructions. cDNA synthesis was performed with SuperScript II Reverse Transcriptase (Invitrogen #18064014) using 0.1–1 μg of total RNA as recommended by the manufacturer. The resulting cDNA was diluted 6‐fold (to fit the genomic DNA calibration curve) and 2 μl used as a template in a 20 μl qPCR performed with the SensiFAST SYBR No‐ROX Kit (#BIO‐98005) reagent and the Bio‐Rad CFX96 instrument. Absolute quantification of target genes alongside the normalising housekeeping gene (*gyrB*) was performed using the provided CFX Maestro™ software. The relative quantity of the sample of interest was calculated using a calibration curve of serial dilutions of purified genomic DNA (CFX Maestro Software User Guide, Appendix A). Primers were designed using the Primer3 online resource, ensuring comparable efficiencies and specificity as judged by the presence of a single peak in the melting curve. The primers used are listed in Supplementary Table S4. Relative qPCR units were defined as the ratio between the absolute levels of each gene and the housekeeping gene *gyrB*.

### 4.8 Sample preparation for Cappable‐sequencing and data analysis

Transcription start site determination at single base‐pair resolution was carried out using the method developed in (Ettwiller et al., 2016). Cells grown for 6 h after nitrogen step down in either iron‐only, vanadium or molybdenum‐containing media were used to purify 10 μg of RNA from each sample as described above. The concentration and integrity of the RNAs were verified with a Bioanalyzer (Agilent™) with the RNA 6000 nano kit (Agilent™ #5067–1511) following the manufacturer's instructions. Library preparation and sequencing were performed by Vertis Biotechnologie AG. Analysis of the single‐ended reads from TSS‐enriched and unenriched libraries was based on (Ettwiller et al., 2016). Scripts provided by the authors in the Github repository https://github.com/Ettwiller/TSS were used according to the instructions provided in https://github.com/Ettwiller/TSS/blob/master/README.md. Briefly, reads were aligned to the *A.vinelandii* DJ genome using bowtie2 in local alignment mode (bowtie2 option *‐‐local*). Then *bam2firstbasegtf.pl* was used to trim the mapped reads to their 5′ single nucleotide position and calculate a score and assign a direction to each position. Next, *filter_tss.pl* was used to filter out nucleotide positions based on scores of the corresponding unenriched libraries. Finally, *cluster_tss.pl* was used to cluster nearby nucleotide positions to a single position (with the highest score). For visualisation in IGV or IGB, bespoke Perl and R scripts were used to make bedgraph files from the scores in the GTF files produced by the above scripts. To quantify relative expression levels at the 5′end of transcripts, log2 fold‐changes were calculated between scores in the different experimental conditions (Supplementary Table S7).

### 4.9 Sample preparation for ChIP‐Seq library sequencing and data analysis

The protocol was adapted from the method described in (Batista et al., 2018; Bush et al., 2016). *A. vinelandii* DJ (control), CAA005 (*vnfA1*::3FLAG) and CAA025 (*vnfA3*::3FLAG) strains were grown diazotrophically to an OD_600_ of 0.4 in NIL media containing iron‐only or with the addition of 1 μM vanadium. For each sample, 100 ml of culture was crosslinked with 1% formaldehyde (Sigma #F8775), incubated for 30 min at 30°C with shaking and neutralised with 125 mM of glycine for 5 min on ice. The cells were washed twice in ice‐cold PBS pH 7.4 (Sigma #P4417‐50TAB) and resuspended in 1 ml of IP lysis Buffer (10 mM Tris–HCl pH 8.0, 50 mM NaCl, 0.8% Triton X‐100, 1x protease inhibitor [Roche #11836170001]). The cells were then lysed, and the DNA was sheared by sonication 15 times at an amplitude of 8 microns to obtain fragments between 200 and 1000 base pairs long. The clarified lysate was diluted to 1/3 to a volume of 1.5 ml with IP buffer (50 mM Tris–HCl pH 8.0, 250 mM NaCl, 0.8% Triton X‐100, 1x protease inhibitor) prior to immuno‐precipitation with EZview Red ANTI‐FLAG M2 affinity gel (Sigma#F2426) following the manufacturer instructions. Briefly, 45 μl of beads were washed three times with ice‐cold TBS (Sigma # T5030‐50TAB) and added to the 1500 μl of cross‐linked lysate and incubated in a rotating wheel at 4°C for 18 h then washed four times with IP Buffer. The DNA fragments were then eluted from the beads with 100 μl of IP Elution buffer (50 mM Tris–HCl pH 7.6, 10 mM EDTA, 1% SDS) overnight at 65°C. The beads were removed by centrifugation and the volume of the supernatant was adjusted to 200 μl with TE buffer (10 mM Tris–HCl pH 7.4, 1 mM EDTA) and treated with 3 μl of proteinase K 10 mg/ml (Roche # 03115879001) for 2 h at 55°C. The samples were cleaned by phenol, phenol/chloroform extraction and purified using a Macherey‐Nagel NucleoSpin® Gel and PCR Clean‐up (Catalogue#740609.50) following the manufactured instructions. For library production and sequencing, 100 μl of each sample at 10 to 20 ng. μL^−1^ were sent to Genewiz™. Reads received from Genewiz were aligned to the genome using the align function of Subread (Liao et al., 2013). The number of reads mapping to every 30 nucleotide section of the *A. vinelandii* DJ genome was counted using the featCounts function of Subread. Counts thus obtained were normalised by calculating a local enrichment for every 30 nucleotide section of the genome as the ratio of the density of reads in the section to the density of reads in the surrounding 3000 nucleotides. The ratio of enrichments in ChIP (FLAG) samples and the enrichments in the corresponding control (WT) samples were calculated as log2 fold‐changes. *p*‐values were calculated assuming a normal distribution of log2 fold‐changes and adjusted using the Benjamini and Hochberg method as implemented in R. Genome positions with adjusted *p*‐values <= .001 were selected and genes to the left, right and overlapping positions were listed. For each combination of left, right and overlapping genes, only the most significant genomic position was retained. Further filtering was done according to the direction of genes in relation to the ChIP‐enriched regions (Supplementary Table S8).

### 4.10 Sequencing data

Sequencing data for the Cappable‐seq and ChIP‐seq experiments are available from the ArrayExpress repository (https://www.ebi.ac.uk/arrayexpress/) under accession numbers E‐MTAB‐11706 and E‐MTAB‐11716, respectively.

### 4.11 Sample preparation for Co‐IP and mass spectrometry analysis

Prior to Co‐IP, cultures were grown exactly as described for the ChIP‐seq sample preparation either in the presence or absence of V (Fe‐only conditions). For each strain and growth condition, triplicate exponentially grown cultures (O.D_600nm_ 0.5–0.6) were cross‐linked with 1% formaldehyde (Sigma F8775) at 30°C and 250 rpm. Following cross‐link quenching with 125 mM glycine, cells were collected by centrifugation (6500 × *g*, 4°C, 5 min) at 4°C and washed twice with ice‐cold PBS (Sigma P4417) and resuspended in 1 ml of lysis buffer (150 mM NaCl, 1% Triton® X‐100, 50 mM Tris HCl pH 8.0) amended with 1x cOmplete™ Mini EDTA‐free Protease Inhibitor Cocktail (Roche 118,361,170,001) as recommended by the manufacturer. Samples were then lysed by sonication in a water‐ice bath (8 × 15 s on followed by 15 s off at 8 microns amplitude). After sonication, the lysate was centrifuged twice (16,000 × *g*, 4°C, 5 min) and the supernatant was retained in a fresh tube. VnfA1‐FLAG and VnfA3‐FLAG proteins were pulled down from the cleared protein extract using the μMACS epitope tag protein isolation kit (Miltenyi Biotec FLAG 130–101‐591) and eluted from the μ columns (Miltenyi Biotec 130–042‐701) with SDS‐PAGE sample buffer (50 mM Tris HCl pH 6.8, 50 mM DTT, 1% SDS, 1 mM EDTA, 0.005% bromophenol blue, 10% glycerol) as recommended by the manufacturer. Equivalent amounts of the pulled‐down lysate was then loaded onto a 10% acrylamide resolving gel and ran briefly until the dye front entered the gel (150 V per gel for 3–5 min). Each sample was then cut out and the gel slices were prepared from mass spectrometry as previously described using standard procedures. Briefly, the gel slices were de‐stained with 30% ethanol, washed with 50 mM TEAB buffer pH 8.0 (Sigma), incubated with 10 mM DTT for 30 min at 65°C followed by incubation with 30 mM iodoacetamide (IAA) at room temperature (both DTT and IAA solutions were prepared in 50 mM TEAB). After a final wash step with 50% acetonitrile in 50 mM TEAB, the gel was dehydrated with 100% acetonitrile and dried under a vacuum. Finally, the gels were soaked with 50 mM TEAB containing 10 ng/μl Sequencing Grade Trypsin (Promega) and incubated at 40°C for 8 h. The resulting peptides were prepared for liquid chromatography–tandem mass spectrometry (LC–MS/MS) as described previously (Bender et al., 2017) and identified using an Orbitrap Eclipse™ Tribrid™ mass spectrometer coupled to an UltiMate® 3000 RSLCnano LC system (Thermo Fisher Scientific, Hemel Hempstead, UK). Data were acquired with the following mass spectrometer settings in positive ion mode: MS1/OT: resolution 120 K, profile mode, mass range m/z 300–1800, spray voltage 2800 V, AGC 4e5, maximum injection time of 50 ms; MS2/IT: data‐dependent analysis was performed using HCD and CID fragmentation with the following parameters: top20 in IT rapid, centroid mode, isolation window 1.0 Da, charge states 2–5, threshold 1.0e4, CE = 33, AGC target 1.0e4, max. Inject time 35 ms, dynamic exclusion 1 count, 15 s exclusion, exclusion mass window ±10 ppm. Recalibrated peaklists were generated with MaxQuant 2.0.1.0 (Tyanova et al., 2016) in LFQ mode using the *A. vinelandii* protein sequence database (from Uniprot, downloaded on 24/08/2021, 5241 entries) plus the Maxquant contaminants database (250 entries). The quantitative LFQ results from MaxQuant with default parameters were used together with search results from an in‐house Mascot Server 2.7 (Matrixscience, London, UK) on the same databases. All Mascot searches were collated and verified with Scaffold v.5 (Proteome Software). Proteins were identified using identification probabilities of 99% for proteins (minimal 5 unique peptides) and 95% for peptides. The quantitative spectra value in Scaffold from both the tagged (VnfA1‐FLAG; CAA005 or VnfA3‐FLAG; CAA025) and untagged (wild type, DJ) strains, generated from each of three independent experiments were used for further differential expression analysis using the Benjamini‐Hochberg test for multiple corrections (Benjamini & Hochberg, 1995). The analysis was performed using the statistical functions built into Scaffold v.5 (Proteome Software) following the developer's instructions.

## AUTHOR CONTRIBUTIONS

C.A.A, R.L. and M.B.B. performed experiments, C.A.A., M.B.B. and R.D. designed experiments and analysed the data, G.C. performed bioinformatic analysis, C.O.M. provided proteomics analysis and C.A.A., M.B.B. and R.D. wrote the paper.

## CONFLICT OF INTEREST

The authors have declared no conflict of interest.

## Supporting information


Supinfo S1

Table S7

Table S8

Table S9

## Data Availability

The proteomics data that support the findings of this study are openly available in the ProteomeXchange Consortium (PRIDE) repository at http://doi.org/10.6019/PXD033387 number PXD033387.
