# Supplementary material for: Interactions between paralogous bacterial enhancer‐binding proteins enable metal‐dependent regulation of alternative nitrogenases in Azotobacter vinelandii
Source: Mol Microbiol. 2022 Jun 29;118(1-2):105–24. doi: 10.1111/mmi.14955 (PMC9542535; doi:10.1111/mmi.14955)
Supplement: Supplementary file 1 — Supinfo S1 [file MMI-118-105-s001.pdf]

Supplementary Information for:

Interactions between paralogous bacterial enhancer binding proteins enable metal-dependent regulation of alternative nitrogenases in *Azotobacter vinelandii*

Corinne Appia-Ayme[1], Richard Little[1], Govind Chandra[1], Carlo de Oliveira Martins[2], Marcelo Bueno Batista[1] and Ray Dixon[1]

[1]Department of Molecular Microbiology, John Innes Centre, Norwich NR4 7UH, UK
[2]Department of Biochemistry and Metabolism, John Innes Centre, Norwich NR4 7UH, UK

This file contains:

Supplementary Figures S1- S12

Supplementary Tables S1-S6

Supplementary References

Supplementary Figures

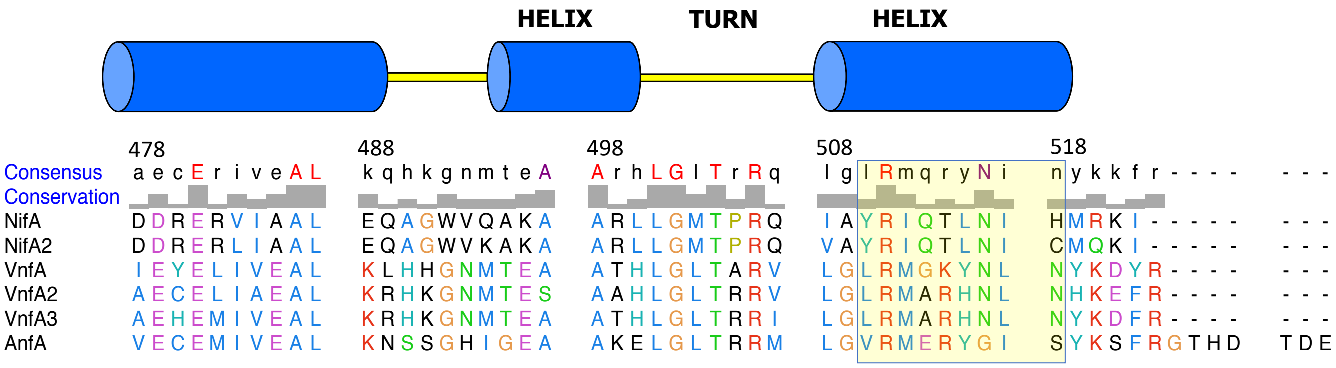

Figure S1. Similarity between the DNA binding domains of bEBPs from *A. vinelandii* that regulate the three nitrogenases (NifA, VnfA and AnfA) and their paralogs. Secondary structure was assigned according to the NMR structure of the C-terminal domain of *Klebsiella oxytoca* NifA (Ray *et al.*, 2002) and Alphafold2 (Jumper *et al.*, 2021, Mirdita *et al.*, 2022) models of VnfA1 and VnfA3. The putative recognition helix of each protein is highlighted in pale yellow. Residue numbering above the sequences refers to *A. vinelandii* NifA.

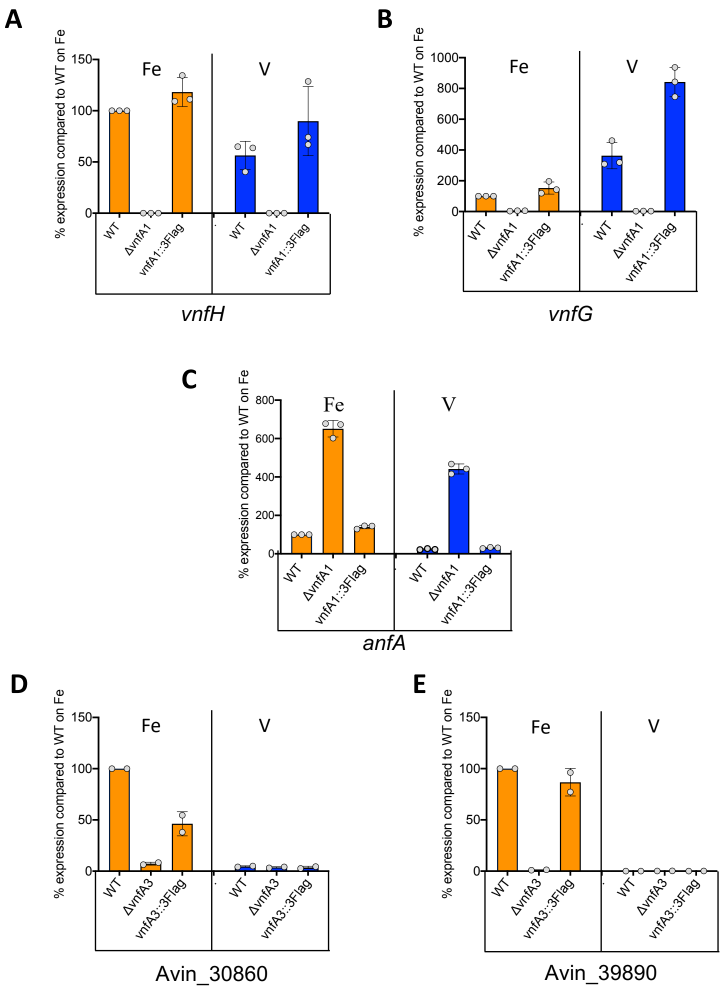

Figure S2. Activity of C-terminal 3XFLAG alleles of *vnfA1* and *vnfA3* when introduced into their native locations in the *A.vinelandii* genome. All graphs show qRT-PCR analysis of the genes indicated at the bottom of each x axis, in either wild type (WT, DJ), deletion *vnfA1* (CAA013) or *vnfA3* (CAA129) mutant strain backgrounds or in strains carrying *vnfA1*::3FLAG (CAA005) or *vnfA3::*3FLAG alleles (CAA025) as indicated beneath each bar. Cultures were incubated either under Fe-only conditions (orange bars) or in the presence of vanadium (blue bars). Data for each gene are normalised to 100% of the wild-type value for that gene when strains are grown under Fe-only conditions. **A,** analysis of v*nfH* transcripts; B, analysis of v*nfG* transcripts; **C,** ability of VnfA1::3FLAG to repress the *anfA* promoter in comparison with wild type and the *vnfA1* deletion mutant; **D** and **E,** ability of VnfA3::3FLAG to activate the Class B promoters upstream of Avin_30860 and Avin_38890 respectively. As in the case of the wild-type strain, activation of these promoters occurs only in the absence of V.

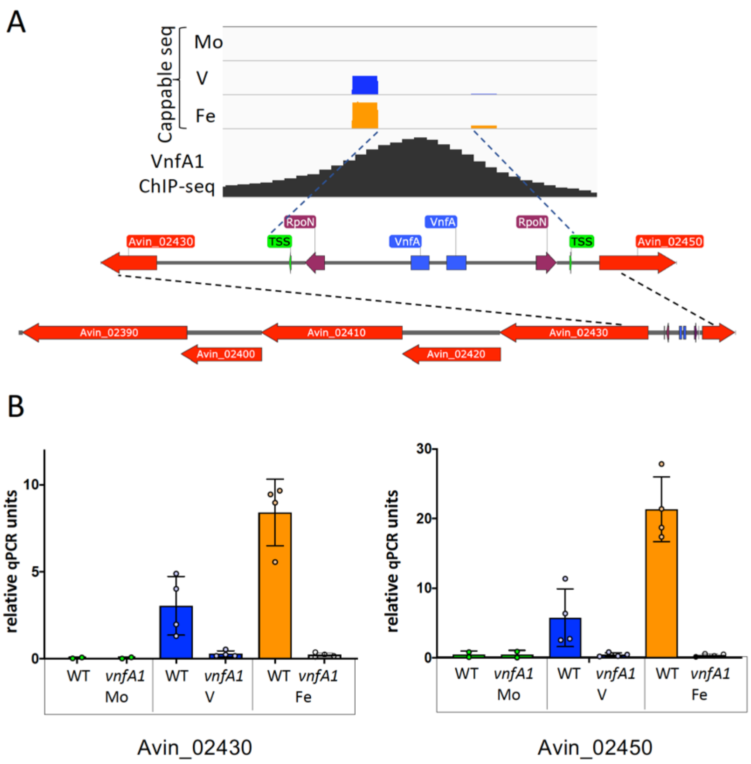

Figure S3. Analysis of the divergent Avin_02430 and Avin_02450 VnfA1 dependent promoters. A, Browser tracks showing Cappable-seq TSS reads in the wild-type strain, incubated under Mo, V or Fe-only conditions as indicated. The bottom track shows the corresponding VnfA1 ChIP-seq peak identified in strain CAA005 (VnfA1-FLAG) grown in the presence of V. A map of the intergenic region, showing the location of TSSs, RpoN consensus sequences and VnfA1 sites identified by MEME analysis are shown beneath the tracks and expanded beneath to show the extent of the VnfA1-dependent Avin_02430 - Avin_02390 operon. **B,** qRT-PCR analysis of Avin_02430 (left) and Avin_02450 (right) in wild-type (DJ) and *vnfA1* deletion (CAA013) strains as indicated, incubated in Mo (green bars), V (blue bars) and Fe-only conditions (orange bars).

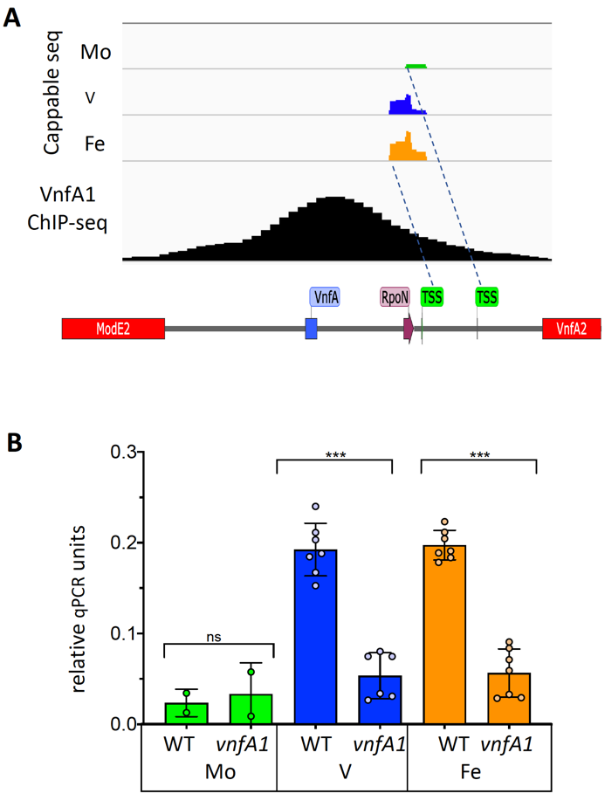

Figure S4. Analysis of the *vnfA2* promoter. A, Browser tracks showing Cappable-seq TSS reads in the wild-type strain, incubated under Mo, V or Fe-only conditions as indicated. The bottom track shows the corresponding VnfA1 ChIP-seq peak identified in strain CAA005 (VnfA1-FLAG) grown in the presence of V. A map of the *modE2-vnfA2* intergenic region is shown below the browser tracks. There are two TSSs for *vnfA2*. The downstream TSS is active under all conditions tested, whereas the upstream RpoN-dependent TSS is activated in V and Fe only conditions. A putative VnfA binding site identified by MEME analysis is also shown. **B,** qRT-PCR analysis of *vnfA2* in wild type (DJ) and the *vnfA1* deletion strain (CAA013) incubated in the presence of Mo (green), V (blue) and Fe-only (orange) conditions. *vnfA2* is apparently expressed at a basal level under all three conditions, but full activation in V and Fe-only conditions is partially dependent on *vnfA1*. ANOVA analysis with Sidak's multiple comparisons was used to compare means. ns, indicates non-significant, *** *P* value < 0.0001**.**

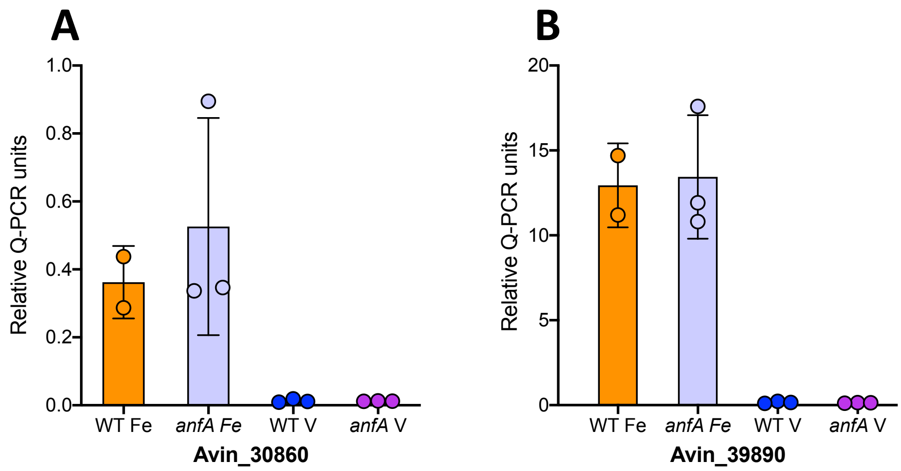

Figure S5. Activation of Class B promoters is not significantly influenced by deletion of an*fA*. qRT-PCR analysis was carried out using the wild-type strain (DJ) or the *ΔanfA* strain (CAA030) as indicated, in either Fe only conditions (orange and light blue bars, respectively) or in the presence of V (blue and purple bars respectively). **A,** qRT-PCR of Avin_30860 **B,** qRT-PCR of Avin_39890.

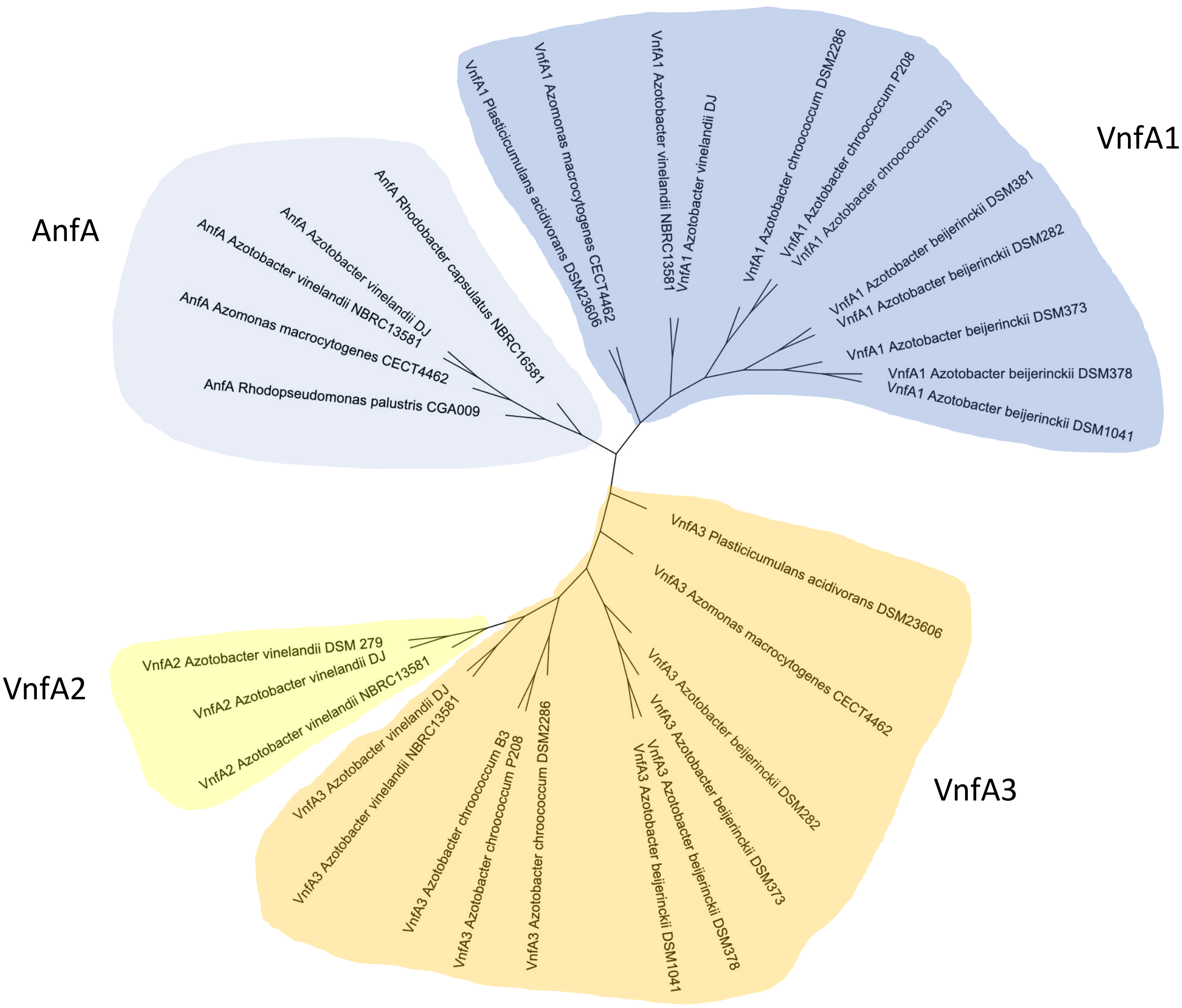

Figure S6. Taxonomic distribution of VnfA paralogs in comparison with AnfA . The phylogenetic tree indicates that VnfA1 , VnfA3 and AnfA are monophyletic and VnfA2 have evolved from VnfA3. Protein sequences were aligned by MAFFT, curated by BMGE and subjected to PhyML analysis at NGphylogeny.fr (Lemoine *et al.*, 2019) The tree was plotted using iTOL (https://itol.embl.de/). Not all representatives of AnfA are shown.

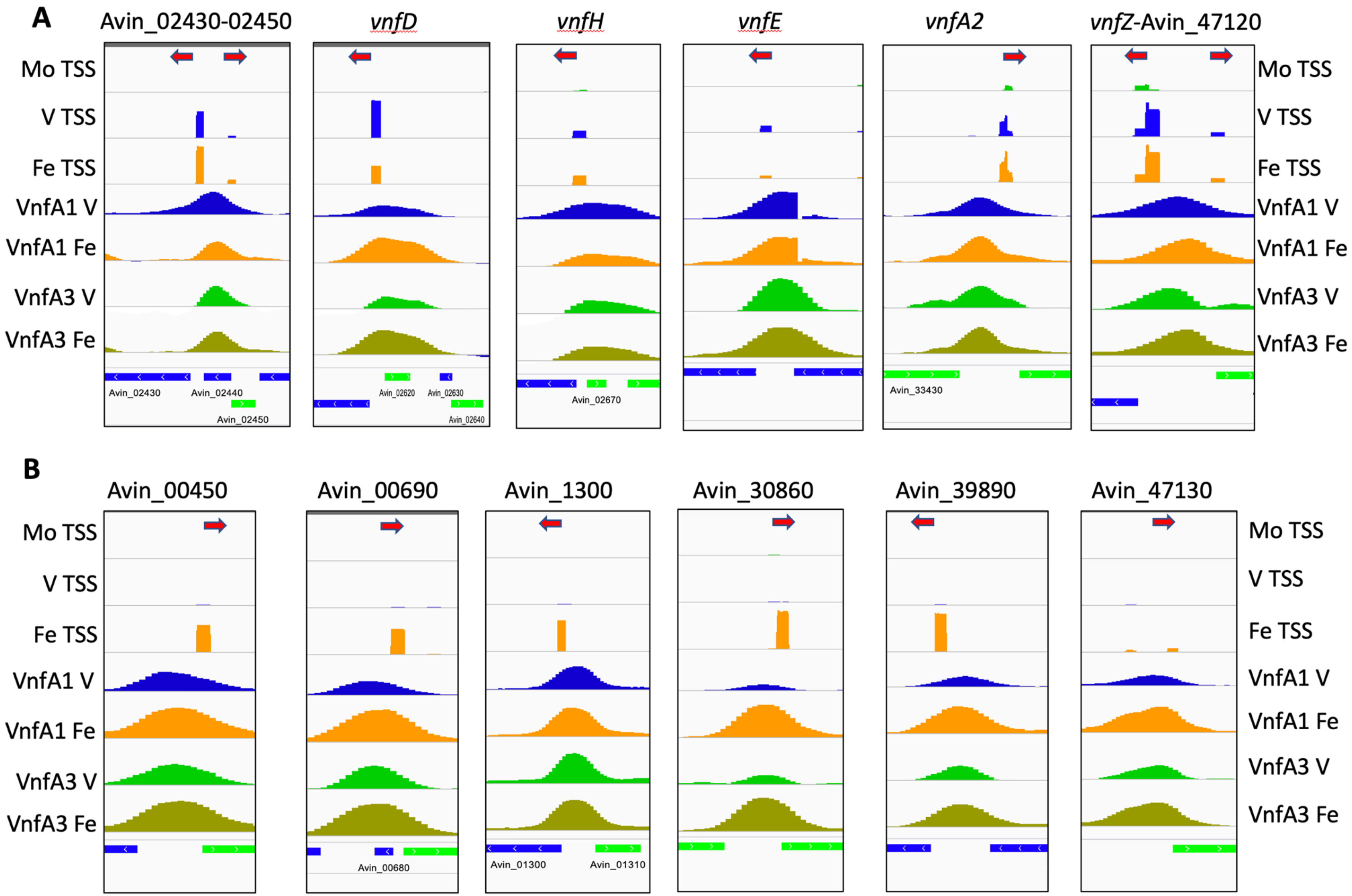

Figure S7. Gallery of TSSs and ChIP-seq peaks for VnfA-activated promoters. IGV browser tracks for each gene include TSS reads for the wild-

type strain (DJ) incubated under Mo, V and Fe-only conditions as indicated (top 3 tracks) with the direction of transcription indicated by the red arrows. The next 4 tracks are ChIPseq peaks for VnfA1 and VnfA3 identified in strains CAA013 and CAA129 respectively, grown in the presence of V or Fe-only conditions as indicated. The bottom track shows genes assigned in the 2013 NCBI annotation of the *A. vinelandii* DJ genome uid57597 (NC_012560.fna, available at ftp://ftp.ncbi.nlm.nih.gov/genomes/archive/old_refseq/Bacteria/) . **A**, Gallery of Class A genes activated in both V and Fe-only conditions. The VnfA1 ChIPseq peaks for *vnfE* are distorted by the presence of the nucleotide sequence of the 3X FLAG allele encoded at the 3'end of *vnfA1,* which is adjacent to *vnfE*. Genes annotated as Avin_02440, Avin_02620 and Avin_02670 in the bottom track are pseudogenes, which have been removed in the more recent NCBI annotation pipeline. **B,** Gallery of Class B genes activated only in the absence of Mo and V (Fe-only conditions). The gene annotated as Avin_00680 in the bottom track is a pseudogene, removed in the recent NCBI annotation pipeline.

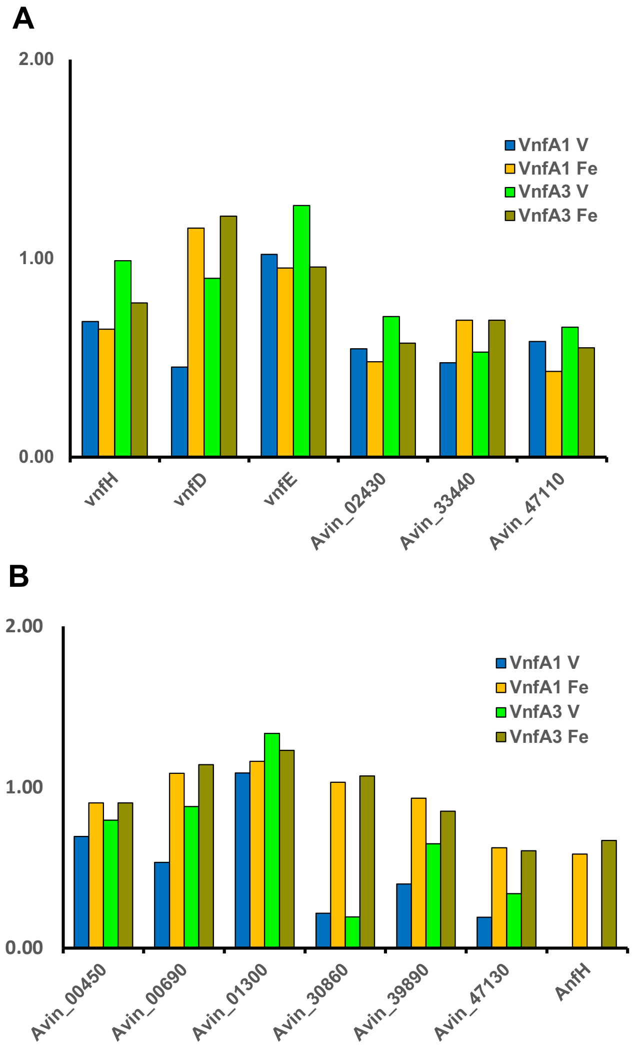

Figure S8. Comparison of log_2 fold changes for VnfA1 and VnfA3 ChIP-seq peaks in V or Fe-only conditions. A, Class A promoters. **B,** Class B promoters and the *anfH* promoter. Data are derived from Supplementary Table S8.

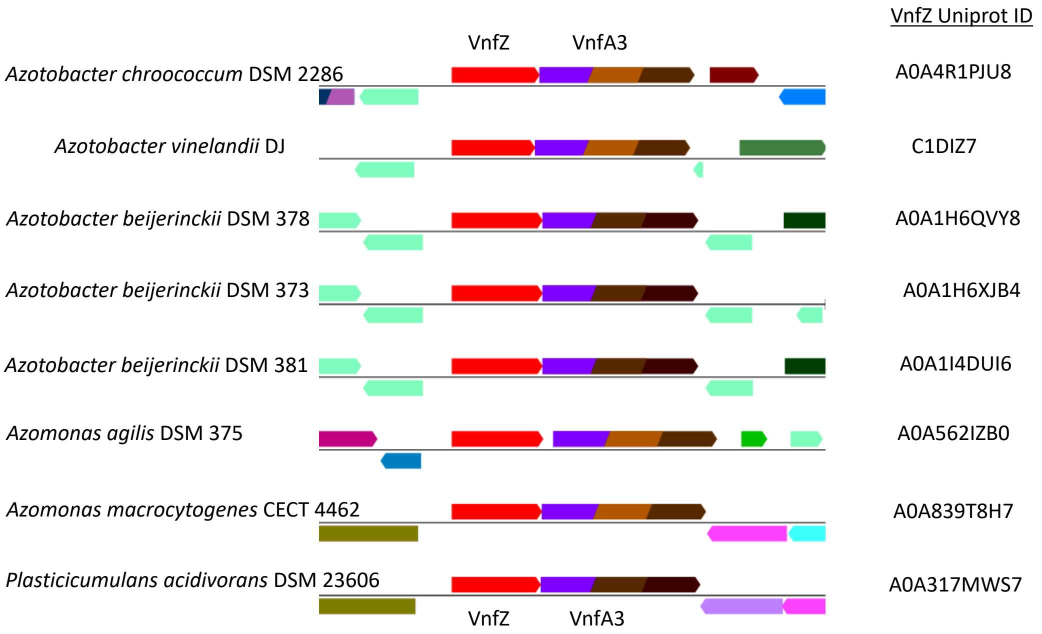

Figure S9. The genes encoding VnfZ and VnfA3 are co-localised in genomes of Azotobacter species and other related diazotrophs that encode vanadium nitrogenase. Gene neighbourhoods were assigned using the EFI-GNT web resource (Zallot *et al.*, 2019) using a protein BLAST query with *A. vinelandii* VnfZ (Uniprot ID C1DIZ7). VnfZ homologs are shown in red and the adjacent VnfA3 sequences in multicolor, reflecting their GAF, AAA+ and DNA binding domains.

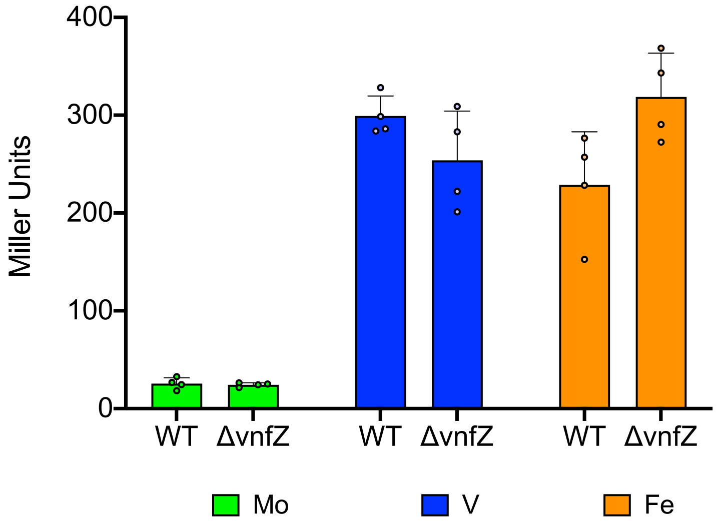

Figure S10. In-frame deletion of *vnfZ* does not affect expression of downstream *vnfA3*. A translational *vnfA3::lacZ* fusion (at codon 253 of *vnfA3*) was introduced into the native location in the *A. vinelandii* genome, either in the wild-type background (WT, strain CAA270) or in the *ΔvnfZ* background (strain CAA271).

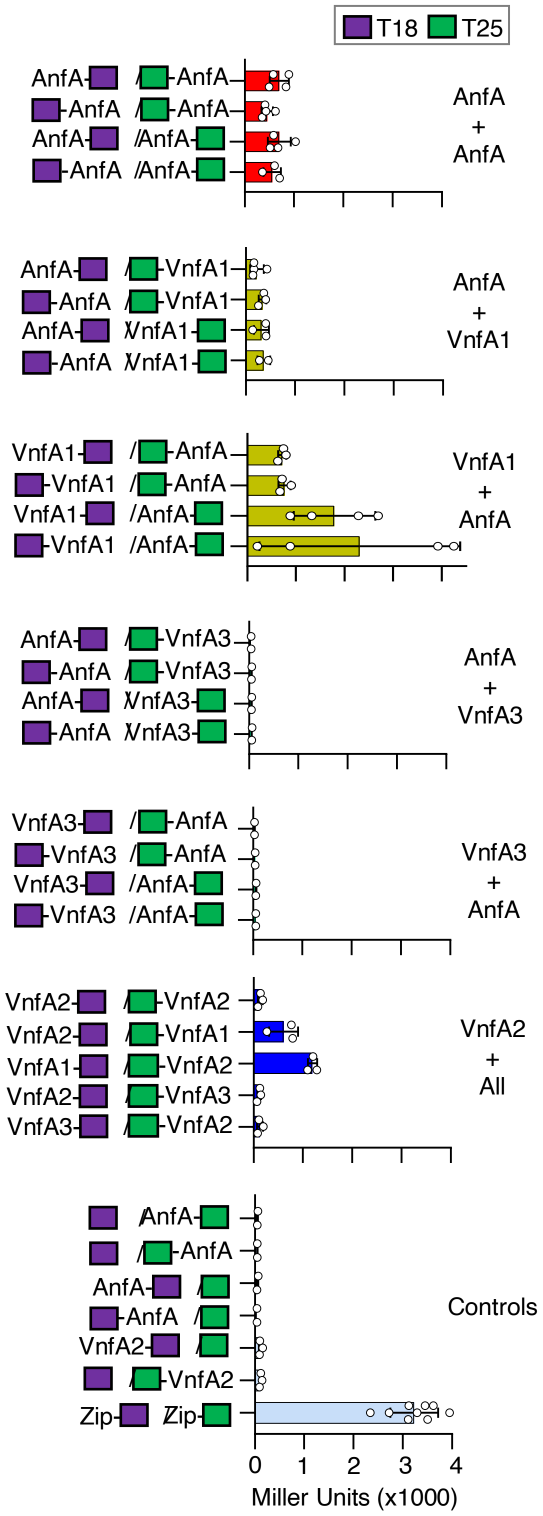

Figure S11. Evidence for interaction between AnfA with VnfA1, and VnfA2 with VnfA1 as determined by β-galactosidase activity reported by the bacterial two hybrid (BACTH) system in *E. coli.* The locations of the T18 and T25 fragments of adenylate cyclase in the fusion proteins are indicated in violet and green rectangles respectively. The light blue bar indicates a positive control using leucine zipper domain fusion proteins (Zip-T25 + Zip-T18).

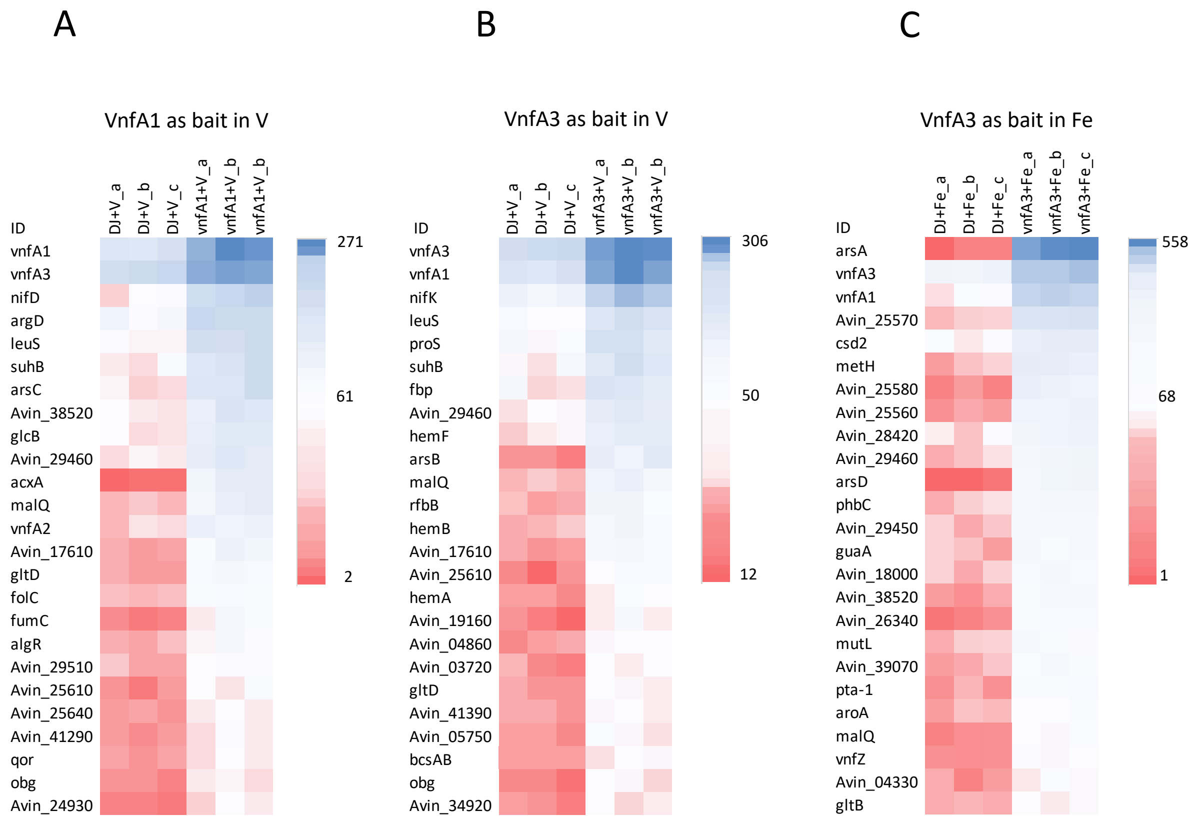

Figure. S12 Overview of the top 25 detected proteins after *in vivo* co-immunoprecipitation followed by mass spectrometry. (A) Analysis of VnfA1-FLAG in the presence of V, (**B)** Analysis of VnfA3-FLAG in the presence of V, (**C)** Analysis of VnfA3-FLAG in the absence of V (Fe-only conditions). In each panel, the heat-maps represent the number of identified peptides in each sample after *in vivo* cross-linking and immunoprecipitation using anti-FLAG magnetic beads. The raw data is available in the ProteomeXchange Consortium (PRIDE) repository (PXD033387) and in Supplementary Table S9 . For more details see the Experimental Procedures section and the legend of Figure 4. Panels A, B and C in this figure correlate to panels B, C and D from Figure 4, respectively.

Supplementary Tables

Table S1: VnfA1-activated promoters identified by correlation of Cappable-seq and ChiP-seq analysis and verification by qRT-PCR.

Promoter	Product description	VnfA1 ChIP-seq pval		Fold change qRT-PCR WT/*vnfA1*	
		V	Fe	V	Fe
Activated in both V and Fe only conditions (Class A)					
vnfH	V nitrogenase iron protein	2.73E-74	7.39E-16	4815 ± 1337 n=6	1618 ± 701 n=6
vnfD *	V nitrogenase alpha subunit	2.77E-32	3.14E-52	318 ± 169 n=6	57 ± 22 n=6
vnfE	V nitrogenase FeV-co biosynthesis protein	3.46E-166	8.48E-36	199 ± 18 n=6	24 ± 10 n=6
Avin_02430	ABC-type iron transport system, substrate binding protein	4.86E-47	2.07E-08	18 ± 9 n=3	32 ± 6 n=3
Avin_02450	hypothetical protein	4.86E-47	2.07E-08	23 ± 12 n=3	52 ± 13 n=3
Avin_33440 (*vnfA2*)	sigma54-dependent activator protein, VnfA2	1.56E-35	2.31E-16	2.96 ± 1.56 n=8	2.25 ± 0.43 n=8
Avin_47110 (*vnfZ*)	phosphonate binding protein	1.23E-53	1.33E-06	3 ± 0.6 n=3	6 ± 1 n=3
Avin_47120	hypothetical protein	1.23E-53	1.33E-06	85 ± 33 n=6	25 ± 6 n=6
Activated in Fe only conditions (Class B)					
Avin_00450	ABC transporter, substrate binding protein	1.43E-76	5.13E-32	2 ± 0.4 n=5	46 ± 18 n=6
Avin_00690	acid phosphatase, V-dependent haloperoxidase	4.12E-45	8.98E-68	6 ± 3 n=5	243 ± 61 n=6
Avin_01300 (*modA2*)	periplasmic molybdate binding protein	6.06E-189	8.98E-53	8 ± 3 n=6	513 ± 99 n=7
Avin_30860	TonB-dependent siderophore receptor	5.31E-07	5.03E-42	1 ± 0.45 n=5	61 ± 21 n=6
Avin_39890	TonB-dependent siderophore receptor	5.52E-25	3.17E-34	0.5 ± 0.23 n=5	332 ± 177 n=6
Avin_47130	TonB-dependent siderophore receptor	2.41E-05	9.21E-15	1 ± 0.48 n=4	23 ± 11 n=5

* qRT-PCR quantification was performed on *vnfG*, the second gene in the *vnfD-vnfG-vnfK* operon

Table S2 - Strains used in this study

Name	Description	Reference or source
E. coli strains		
K-12 MG1655	F- lambda- *ilvG*- *rfb*-50 *rph*-1	(Blattner *et al.*, 1997)
NEB 5-alpha	Derivative of DH5α. T1 phage resistant and *endA* deficient. Used for cloning and plasmid maintenance	NEB
S17-1 λpir	*TpR SmR recA, thi, pro, hsdR*-M+RP4: 2-Tc:Mu: Km Tn7 λpir<br>Used for conjugation	(de Lorenzo *et al.*, 1993)
BTH101	F−, *cya*-99, *araD*139, *galE*15, *galK*16, *rpsL*1, *hsdR*2, *mcrA*1, *mcrB*1	(Karimova *et al.*, 1998)
A. vinelandii strains		
Av DJ	ATCC BAA-1303. High frequency transforming variant of *A. vinelandii* UW generated in 1984 by Dennis Dean	(Setubal *et al.*, 2009)
CAA005[a]	*vnfA1::3FLAG, rpoB113* , Rif^R	This work
CAA013[a]	*ΔvnfA1, rpoB113,* Rif^R	This work
CAA022[c]	*modE2::kan*	This work
CAA023[a]	*ΔmodE1, rpoB113* , Rif^R	This work
CAA025[a]	*vnfA3::3FLAG, rpoB113,* Rif^R	This work
CAA026[a]	*ΔmodE1, modE2::kan, rpoB113,* Rif^R derived from CAA023	This work
CAA030[a]	*ΔanfA, rpoB113,* Rif^R	This work
CAA129[b]	*ΔvnfA3*	This work
CAA140[c]	*anfA::lacZ* transcriptional fusion inserted into the *algU* locus, Tmp^R	This work
CAA144[ac]	*ΔanfA, anfA::lacZ* transcriptional fusion inserted into the *algU* locus, Tmp^R	This work
CAA148[ac]	*ΔvnfA1, anfA::lacZ* transcriptional fusion inserted into the *algU* locus	This work
CAA171[b]	*ΔvnfA2*	This work
CAA206[b]	*ΔvnfZ* M131-K279 in frame deletion	This work
CAA270[b]	Translational fusion *vnfA3::lacZ*	This work
CAA271[b]	*ΔvnfZ,* translational fusion *vnfA3::lacZ*	This work
CAA299[b]	*ΔvnfA3, ΔvnfDGK, ΔnifDGK*	This work
CAA293[b]	*ΔvnfZ, ΔvnfDGK, ΔnifDGK*	This work
CAA292[ab]	*ΔanfA, ΔvnfDGK, ΔnifDGK,* Rif^R	This work
CAA298[b]	*ΔvnfDGK, ΔnifDGK*	This work

Abbreviations: Rif^R: rifampicin, Km^R: kanamycin, Tmp^R : Trimethoprim resistance.

[a] derived by congression

[b] derived by conjugation

[c] derived by transformation

Table S3 – Plasmids used in this study

Name	Description	Reference or source
pGLR2	Km^R, used to amplify the kan gene for the construction of insertion mutants	(Benedetti *et al.*, 2012)
pJet1.2/blunt	Ap^R, cloning vector	CloneJET PCR Cloning Kit, Thermo Scientific
pK18mobsacBKm	Km^R, mobilisable vector with sucrose selection used for the construction of mutants	(Schäfer *et al.*, 1994)
pDB303	Ap^R, Rif^R, congression plasmid, *rpoB113* for selection	Dennis Dean
pUC19	Ap^R, high copy blue/white cloning plasmid	Sigma-Aldrich OGS590
pUT18	Ap^R, cloning vector for the fusion of T18 at the C-terminus of a protein	(Karimova *et al.*, 2001)
pUT18C	Ap^R, cloning vector for the fusion of T18 at the N-terminus of a protein	(Karimova *et al.*, 2001)
pT25	Cm^R, cloning vector for the fusion of T25 at the N-terminus of a protein	(Karimova *et al.*, 2005)
pKNT25	Km^R, cloning vector for the fusion of T25 at the C-terminus of a protein	(Karimova *et al.*, 2005)
pUT18-Zip	Ap^R, fusion of T18 at the C-terminus of the zip peptide for positive control	(Karimova *et al.*, 2001)
pKNT25-Zip	Km^R, fusion of T25 at the C-terminus of the zip peptide for positive control	(Karimova *et al.*, 2001)
pTJ1	TmpR, ApR. Used to amplify the *dhfR*II gene for Tmp resistance	(Damron *et al.*, 2013)
pPR34	Ap, lab stock used to construct pRL345	(Söderbäck *et al.*, 1998)
pJetmodE1	*ΔmodE1*, Ap^R	This work
pJetmodE2Km	*ΔmodE2::Km*, Ap^R, Km^R,	This work
pUCΔvnfA1	Ap^R, 800pb upstream and downstream of *vnfA1* cloned in pUC19 to create a gene deletion by congression.	This work
pRL345	Ap^R, 1kb upstream and downstream of *anfA* cloned in pPR34 to create an *anfA* deletion by congression.	This work
pJetvnfA1::3FLAG	Ap^R, 800bp upstream and downstream of *vnfA1* cloned in pJet1.2to introduce a triple FLAG-tag allele at the C-terminus of VnfA1.	This work
pJetvnfA3::3FLAG	Ap^R, 800bp upstream and downstream of *vnfA3* cloned in pJet1.2 to introduce a triple FLAG-tag allele at the C-terminus of VnfA1	This work
pUCanfA::lacZ	Tmp^R, Ap^R, transcriptional fusion of the *anfA* promoter and *lacZ* flanked by 800pb of the *algU-mucA* region of *A. vinelandii* chromosome.	This work
pKmob ΔvnfA3	Km^R, 800bp upstream and downstream of *vnfA3* cloned in pK18mobSacB for the	This work

	construction of deletion mutant CAA129 by conjugation.	
pKmob ΔvnfA2	Km^R, 800bp upstream and downstream of *vnfA2* cloned in pK18mobsacB for the construction of deletion mutant CAA171 by conjugation.	This work
pKmob ΔvnfZ M131-K279	Km^R, deletion from M131to K279 in *vnfZ* cloned in the pK18mobsacB for the construction of an in-frame deletion mutant CAA206 by conjugation.	This work
pKTmob vnfA3::lacZ	Km^R, translational fusion *vnfA3::lacZ* used to construct strains CAA270 and CAA271	This work
pKmob ΔvnfDGK	Km^R, deletion of *vnfD* to *vnfK* cloned in pK18mobsacB for the construction of a deletion mutants CAA292, CAA293, CAA298 and CAA299 by conjugation.	This work
pKmobΔnifDK	Km^R, deletion of *nifD* to *nifK* cloned in pK18mobsacB for the construction of a deletion mutants CAA292, CAA293, CAA298 and CAA299 by conjugation.	This work
pT18vnfA1	ApR, fusion of T18 at the C-terminus of VnfA1	This work
pTC18vnfA1	Ap^R, fusion of T18 at the N-terminus of VnfA1	This work
pT25vnfA1	Cm^R, fusion of T25 at the N-terminus of VnfA1	This work
pNT25vnfA1	Km^R, fusion of T25 at the C-terminus of VnfA1	This work
pT18vnfA3	Ap^R, fusion of T18 at the C-terminus of VnfA3	This work
pTC18vnfA3	ApR, fusion of T18 at the N-terminus of VnfA3	This work
pT25vnfA3	Cm^R, fusion of T25 at the N-terminus of VnfA3	This work
pNT25vnfA3	Km^R, fusion of T25 at the C-terminus of VnfA3	This work
pT18anfA	Ap^R, fusion of T18 at the C-terminus of AnfA	This work
pTC18anfA	Ap^R, fusion of T18 at the N-terminus of AnfA	This work
pT25anfA	Cm^R, fusion of T25 at the N-terminus of AnfA	This work
pNT25anfA	Km^R, fusion of T25 at the C-terminus of AnfA	This work
pT18nifA	Ap^R, fusion of T18 at the C-terminus of NifA	This work
pTC18nifA	Ap^R, fusion of T18 at the N-terminus of NifA	This work
pT25nifA	Cm^R, fusion of T25 at the N-terminus of NifA	This work
pNT25nifA	Km^R, fusion of T25 at the C-terminus of NifA	This work
pT18 vnfA2	Ap^R, fusion of T18 at the C-terminus of vnfA2	This work
pT25 vnfA2	Cm^R, fusion of T25 at the N-terminus of VnfA2	This work
pT18vnfZ	Ap^R, fusion of T18 at the C-terminus of VnfZ	This work

pTC18vnfZ	Ap^R, fusion of T18 at the N-terminus of VnfZ	This work
pT25vnfZ	Cm^R, fusion of T25 at the N-terminus of VnfZ	This work
pNT25vnfZ	Km^R, fusion of T25 at the C-terminus of VnfZ	This work

Abbreviations: Rif^R: rifampicin, Ap^R: ampicillin, Km^R: kanamycin, Cm^R: chloramphenicol, Tmp^R : Trimethoprim resistance.

Table S4 - Primers used in this study

ID	Sequence 5'---> 3'	gene target/use
qRT-PCR primers		
509	CTGCCCTTCAACTGGTTCGA	Avin_00450
510	CGGATATCCTCGTCGAAGCG	Avin_00450
585	CTGATCGCCGCTCTGTTAAT	Avin_00690
586	GGGTCAGATCCAGTCTGTCG	Avin_00690
503	CACCATCAGTTCCGGCTCTT	Avin_01300 (*modA2*)
504	TTTTCCGGGCTCTTCTGGTC	Avin_01300 (*modA2*)
497	CAAGGCCGAGGAAGAAGCC	Avin_02450
498	GACGACGGTCCGGAAGTTC	Avin_02450
551	ATGGAGCTGTCGATGCTGAC	Avin_02390
552	GGCGCGTAGTAGTTGAGCTT	Avin_02390
549	GATCGTCGGCTTCCTGTATC	Avin_02400
550	TAGATCCAGCGGAGGAAGTC	Avin_02400
547	GTGGTGCTGATCGACGAGA	Avin_02410
548	GGTGGAAATCAGCACGATCT	Avin_02410
545	ACCAAGGGCGACATCATTT	Avin_02420
546	GTGAGACCGTTGCAGAACAG	Avin_02420
537	GTACCTGGAATTCGGTCAGG	Avin_02430
538	ACGTCCTGGCTGTTCGAG	Avin_02430
577	CGCATCGAACGTTCCTATTT	Avin_30860
578	CTTGACGAGGGTGTCGAACT	Avin_30860
579	GGAAGTCACCGAGTCCAGAA	Avin_39890
580	CGGCTCAGGTTGTATTCCAT	Avin_39890
495	CAAGGTGGAGGAACTGGACC	Avin_47120
496	TCCTTGGGAAAGAGCATGCC	Avin_47120
593	GAAGACCACCCTCAAGTTCG	Avin_47130
594	GGTGGTGTAGAGGCCGTAGA	Avin_47130
141	TCCACTACAACAGCCCCAAT	*anfA*
142	TTTTCATGGCCGAAGAGTTC	*anfA*
54	TTTGTCTGGCCGACGACTAT	*anfG*
55	ATCCATGCGGTCTTTCAGTC	*anfG*
147	CAAGAAGCACAAGGTGACGA	*gyrB*
148	TGGTCTGCGAACTGAACTTG	*gyrB*
445	GTTCGCGCTTCACTTATGC	*modA*
446	TGCTTGAAGGCGTTCTTCTT	*modA*
447	GACCGTGCTCGGCTTCTAC	*modB*
448	GAAGGTGAACGGCAATGTG	*modB*

270	GCCATCGATACCATGAACAA	*modE1*
271	GCCGTAGTCGGTGAGCTG	*modE1*
268	AAGGAAATCTTCGCCTGGAT	*modE2*
269	GACCTCACCCCACAAGACAT	*modE2*
133	GCAAGTACGGCTTCGAGAAC	*nifA*
134	GTACGGTGCTGTTCCACTTG	*nifA*
135	CTCTACGACAACGCCGAACT	*vnfA1*
136	CTCGACGTTCTCCACCAAGT	*vnfA1*
137	CCATAGGCTGATCGAGAAGG	*vnfA2*
138	CGTTGTAGTGGATGGCACTG	*vnfA2*
139	GGGTTGATCGAGAAGGTCAC	*vnfA3*
140	GCTGCTGCTGTTGTAGTGGA	*vnfA3*
541	GCATGACCACCGAACTGTC	*vnfE*
542	GCTGGTAGCGCTCGACTG	*vnfE*
178	CAAGGCCCAGGAAATCTACA	*vnfH*
179	GAGTGGGCGTATTTCACGAT	*vnfH*
463	GCCGTACAGGACAACATCGT	*vnfU*
464	TCGAGCACCTTGACCAGTTC	*vnfU*
180	AAGTTGCTGGTCGGTAGCAC	*vnfZ*
181	GCGACTCTGCAGGATGTAGG	*vnfZ*
Verification of ChiP-Seq target enrichment		
477	TAGGAACCTCCGTTGATTCG	*vnfH* promoter
478	ATCGACTTATCGATCCTTGTGG	*vnfH* promoter
491	CTCAACTCTCGTCGGCACAA	*vnfH* promoter
492	GCCACCCTTGCCGTAAATTG	*vnfH* promoter
493	CGAGCACCCCAGTACCATG	*vnfH* promoter
494	ACTCCATAAATCCAGGCCAGG	*vnfH* promoter
489	GCGTACAGGGATCGTTCG	*vnfE* promoter
490	CTGGAGCAAAGGTCGCGC	*vnfE* promoter
638	CGAAGTCCGTTTCCGTACTC	Avin_01300 promoter
639	CCTGTTTCCTTGGGAGATTG	Avin_01300 promoter
640	GTCGAATGTTCCGGGAAGTA	Avin_30860 promoter
641	TCGCGACACGAAAGTACAAC	Avin_30860 promoter
642	GCAAAAAGTCGCTGGTATGG	Avin_39890 promoter
643	TCTCGGCATCTGGTACTTCC	Avin_39890 promoter
644	GAATTACCCAGGCGAACATC	Avin_47130 promoter

645	GGTTCCTGGATGTCGAAAAA	Avin_47130 promoter
Primers used for the construction of BATCH plasmids		
595	AGGAAACAGCTATGACCATGATGTCCAGCCTCCCCCAATAC	Construction of pT18vnfA1
596	GCTGAATTCGAGCTCGGTACGCGGTAGTCCTTGTAGTTGAG	Construction of pT18vnfA1
597	AGGAAACAGCTATGACCATGATGAAACCGCATTCGGCG	Construction of pT18vnfA3
598	GCTGAATTCGAGCTCGGTACGCGGAAATCCTTGTAGTTCAG	Construction of pT18vnfA3
599	GTACCGAGCTCGAATTCAG	Cloning in the pUT18
600	CATGGTCATAGCTGTTTCC	Cloning in the pUT18
601	GTACCGAGCTCGAATTCATC	Cloning in the pUTC18
602	CCGGGGATCCTCTAGAGTC	Cloning in the pUTC18
603	CGACTCTAGAGGATCCCCGGATGAAACCGCATTCGGCG	Construction of pTC18vnfA3
604	GATGAATTCGAGCTCGGTACGCGGAAATCCTTGTAGTTCAG	Construction of pTC18vnfA3
605	CGACTCTAGAGGATCCCCGGATGTCCAGCCTCCCCCAATAC	Construction of pTC18vnfA1
606	GATGAATTCGAGCTCGGTACGCGGTAGTCCTTGTAGTTGAG	Construction of pTC18vnfA1
607	CCTAAGTAACTAAGAATTCGGC	Cloning in pKT25
608	TACCCGGGGATCCTCTAG	Cloning in pKT25
609	CTCTAGAGGATCCCCGGGTAATGTCCAGCCTCCCCCAATAC	Construction of pT25vnfA2
610	CGAATTCTTAGTTACTTAGGGCGGTAGTCCTTGTAGTTGAG	Construction of pT25vnfA2
611	CTCTAGAGGATCCCCGGGTAATGAAACCGCATTCGGCG	Construction of pT25vnfA3
612	CGAATTCTTAGTTACTTAGGGCGGAAATCCTTGTAGTTCAG	Construction of pT25vnfA3
613	CGGGTACCGAGCTCGAATTC	Cloning in pKNT25
614	CATGGTCATAGCTGTTTCCTG	Cloning in pKNT25
615	AGGAAACAGCTATGACCATGATGTCCAGCCTCCCCCAATAC	Construction of pNT25vnfA1
616	GAATTCGAGCTCGGTACCCGGCGGTAGTCCTTGTAGTTGAG	Construction of pNT25vnfA1
617	AGGAAACAGCTATGACCATGATGAAACCGCATTCGGCG	Construction of pNT25vnfA3
618	GAATTCGAGCTCGGTACCCGGCGGAAATCCTTGTAGTTCAG	Construction of pNT25vnfA3

619	CCTAAGTAAGTAAGAATTCGGC	Cloning in pKT25
620	CGAATTCTTACTTACTTAGGGCGGTAGTCCTTGTAGTTGAG	Construction of pT25vnfA1
621	CGAATTCTTACTTACTTAGGGCGGAAATCCTTGTAGTTCAG	Construction of pT25vnfA3
630	AGGAAACAGCTATGACCATGGTGGTGGAACGACTCAACG	Construction of pT18vnfZ
631	GCTGAATTCGAGCTCGGTACTGGTCGACCTCCGGATGC	Construction of pT18vnfZ
632	CGACTCTAGAGGATCCCCGGGTGGTGGAACGACTCAACG	Construction of pTC18vnfZ
633	GATGAATTCGAGCTCGGTACTGGTCGACCTCCGGATGC	Construction of pTC18vnfZ
634	CTCTAGAGGATCCCCGGGTAGTGGTGGAACGACTCAACG	Construction of pT25vnfZ
635	CGAATTCTTACTTACTTAGGTGGTCGACCTCCGGATGC	Construction of pT25vnfZ
636	AGGAAACAGCTATGACCATGGTGGTGGAACGACTCAACG	Construction of pNT25vnfZ
637	GAATTCGAGCTCGGTACCCGTGGTCGACCTCCGGATGC	Construction of pNT25vnfZ
660	AGGAAACAGCTATGACCATGATGAGCGATCAGGCAACC	Construction of pT18anfA
661	GCTGAATTCGAGCTCGGTACCTCATCGGTATCATGCGTACC	Construction of pT18anfA
662	CGACTCTAGAGGATCCCCGGATGAGCGATCAGGCAACC	Construction of pTC18anfA
663	GATGAATTCGAGCTCGGTACTCACTCATCGGTATCATG	Construction of pTC18anfA
664	CTCTAGAGGATCCCCGGGTAATGAGCGATCAGGCAACC	Construction of pT25anfA
665	CGAATTCTTACTTACTTAGGTCACTCATCGGTATCATG	Construction of pT25anfA
666	AGGAAACAGCTATGACCATGATGAGCGATCAGGCAACC	Construction of pNT25anfA
667	GAATTCGAGCTCGGTACCCGCTCATCGGTATCATGCGTACC	Construction of pNT25anfA
668	AGGAAACAGCTATGACCATGATGAATGCAACCATCCCTCAG	Construction of pT18nifA
669	GCTGAATTCGAGCTCGGTACGATCTTGCGCATGTGGATG	Construction of pT18nifA
670	CGACTCTAGAGGATCCCCGGATGAATGCAACCATCCCTCAG	Construction of pTC18nifA
671	GATGAATTCGAGCTCGGTACTCAGATCTTGCGCATGTGG	Construction of pTC18nifA
672	CTCTAGAGGATCCCCGGGTAATGAATGCAACCATCCCTCAG	Construction of pT25nifAfwd
673	CGAATTCTTACTTACTTAGGTCAGATCTTGCGCATGTGG	Construction of pT25nifA

674	AGGAAACAGCTATGACCATGATGAATGCAACCATCCCTCAG	Construction of pNT25nifA
675	GAATTCGAGCTCGGTACCCGGATCTTGCGCATGTGGATG	Construction of pNT25nifA
684	AGGAAACAGCTATGACCATGAAAGTTCTCAGATGCGGATTG	Construction of pT18vnfZ
685	CGACTCTAGAGGATCCCCGGAAAGTTCTCAGATGCGGATTG	Construction of pTC18vnfZ
686	CTCTAGAGGATCCCCGGGTAAAAGTTCTCAGATGCGGATTG	Construction of pT25vnfZ
687	AGGAAACAGCTATGACCATGAAAGTTCTCAGATGCGGATTG	Construction of pNT25vnfZ
700	AGGAAACAGCTATGACCATGATGGGCGAATGCCATACC	Construction of pT18vnfA2
701	GCTGAATTCGAGCTCGGTACCCGAAACTCCTTGTGGTTC	Construction of pT18vnfA2
702	CTCTAGAGGATCCCCGGGTAATGGGCGAATGCCATACC	Construction of pT25vnfA2
703	CGAATTCTTACTTACTTAGGTCACCGAAACTCCTTGTG	Construction of pT25vnfA2
797	AGGAAACAGCTATGACCATGATGAGCCCACCTCCCGAG	Construction of pT18ExtvnfA2
798	CTCTAGAGGATCCCCGGGTAATGAGCCCACCTCCCGAG	Construction of pT25ExtvnfA2
Primers used for mutant construction		
15	AGGCGGTCGAACTGTACTTG	Construction of pJetvnfA1::3FLAG
16	CTTGGAGCAGACCACCATGT	Construction of pJetvnfA1::3FLAG
17	AGTCGATGTCGTGGTCCTTGTAGTCGCCGTCGTGGTCCTTGTAGTCGCGGTAGTCCTTGTAGTTGA	Construction of pJetvnfA1::3FLAG
18	CGACTACAAGGACCACGACATCGACTACAAGGACGATGACGACAAGTGAGCGCGGCGTACCGCCCG	Construction of pJetvnfA1::3FLAG
25	ACATCATCGGCAATTCCAAG	Construction of pJetvnfA1::3FLAG
26	GGTGCATGGTCTGGACCTC	Construction of pJetvnfA1::3FLAG
94	GCCAAGGTCAACGAAGTCAT	Construction of pUCvnfA1
95	GCTTCGACGATGAGTTCGTAAGCAGGATGCTGATGATGC	Construction of pUCvnfA1
96	GCATCATCAGCATCCTGCTTACGAACTCATCGTCGAAGC	Construction of pUCvnfA1

97	AGGCTCTTGGTGCCGTAGA	Construction of pUCvnfA1
809	CAATTCTCATATGCCTTACGGCTGTTGCAAATGATG	Construction of pRL345
810	TTCGTCGAGGGTACGCATGATACCGATGAGTG	Construction of pRL345
811	ATGCGTACCCTCGACGAAGTAATCCGTGTCGAAC	Construction of pRL345
812	GCTAGGATCCGCATGACCGAGCGGAAATTGCAG	Construction of pRL345
162	ATGGCGGTGGCTCGTGCGAC	Construction of pJetmodE1
163	GGTGACCACCACGACCTTGGCGGCCCGGTTGATCGATCC	Construction of pJetmodE1
164	AAGGTCGTGGTGGTCACCGCCGATAGCTGCAAGGCCCTC	Construction of pJetmodE1
165	TGTCCTTGCCCTTGGTCAGG	Construction of pJetmodE1
166	GATCGGCTCGTACAGATCG	Construction of pJetmodE1
167	GATGAGGTCCGCTCTCAGG	Construction of pJetmodE1
168	AAAAGGATCCGTCGCTTGGTCGGTCATTTC	Construction of pJetmodE2Km
169	AAAAGAATTCTATTTCTAGGCACAGCTTCACG	Construction of pJetmodE2Km
170	AAAACTCGAGGTCACTTCCCGGATGATTTC	Construction of pJetmodE2Km
171	AAAAGAATTCGTTGATCGAGCCCTCTCGT	Construction of pJetmodE2Km
198	AAAAGGATCCGACTAGCGAGGTGACCCTGA	Construction of pJetmodE2Km
199	AAAATCTAGACCTGCTGGACGAAGATCC	Construction of pJetmodE2Km
192	AACTGGTGGCCAGCGCCATC	Construction of pUCvnfA3::3FLAG
193	AGAGCGTCATCGAGAGCGAG	Construction of pUCvnfA3::3FLAG
194	TACAGCAGGGAGGTGATGTG	Construction of pUCvnfA3::3FLAG
195	TCAGCGAACGGTACAGCTCG	Construction of pUCvnfA3::3FLAG
196	AGTCGATGTCGTGGTCCTTGTAGTCGCCGTCGTGGTCCTTGTAG TCGCGGAAATCCTTGTAGTTCAG	Construction of pUCvnfA3::3FLAG

197	CGACTACAAGGACCACGACATCGACTACAAGGACGATGACGA CAAGTGAGGTCCGGCTGTACGGTGC	Construction of pUCvnfA3::3FLAG
363	GCCCGCTTTCCAGTCGGG	Vector amplification for cloning in pUC19
368	TAAGCCAGCCCCGACACC	Vector amplification for cloning in pUC19
410	CGTTCGGCTGCGGCGAGC	Vector amplification for cloning in pK18mobsacB
415	GAAACGGTGCTGACCCCG	Vector amplification for cloning in pK18mobsacB
411	CCGCTCGCCGCAGCCGAACGGGAGCGCCGCCTGGGTCT	construction pKmob ΔvnfA3
412	GACCTCAGCGTTTCATGGTCGACCTCCGGATGCG	construction pKmob ΔvnfA3
413	GACCATGAAACGCTGAGGTCCGGCTGTA	construction pKmob ΔvnfA3
414	TCCGGGGTCAGCACCGTTTCAACGGTACAGCTCGGCCTC	construction pKmob ΔvnfA3
589	GCTCGCCGCAGCCGAACGACGCTCTTTCAAACTCACCG	construction pKmobΔ vnfA2
590	GGCTCACCGAAACTCCTTGTGCAACAGATCGGTATGGCAT	construction pKmob ΔvnfA2
591	ATGCCATACCGATCTGTTGCACAAGGAGTTTCGGTGAGCC	construction pKmob ΔvnfA2
592	CGGGGTCAGCACCGTTTCCATCGTGGTGGCATTCTGTC	construction pKmob ΔvnfA2
654	GCTTCACGCTGCCGCAAG	Vector amplification for cloning in pK18mobsacB
655	TGTTATCCGCTCACAATTCCACAC	Vector amplification for cloning in pK18mobsacB
656	GGAATTGTGAGCGGATAACAGCACTCGGCGGGCTCTTC	Construction of pKmob ΔvnfZ M131-K279
657	AGGGCAGCTTCATGTGCCGGGGCACCCA	Construction of pKmobΔ vnfZ M131Z-K279

658	CCGGCACATGAAGCTGCCCTGGCGACGC	Construction of pKmob ΔvnfZ M131-K279
659	TGCTTGCGGCAGCGTGAAGCTGCCGATGATGTTGCTGGGC	Construction of pKmob ΔvnfZ M131-K279
775	GAAACGGTGCTGACCCCG	pKTmob-sac amplification
776	CGTTCGGCTGCGGCGAGC	pKTmob-sac amplification
777	CCGCTCGCCGCAGCCGAACGCCATGAAACCGCATTCGGC	Construction of pKmob vnfA3::lacZ
778	CGACGGCCAGGGCGCTGCTGCTGTTGTAG	Construction of pKmob vnfA3::lacZ
779	CAGCAGCGCCCTGGCCGTCGTTTTACAAC	*lacZ* amplification
780	CAGCCGGACCTTATTTTTGACACCAGACCAAC	*lacZ* amplification
781	TCAAAAATAAGGTCCGGCTGTACGGTGC	Construction of pKmob vnfA3::lacZ
782	TCCGGGGTCAGCACCGTTTCTGCTCGACACCCTCGGCC	Construction of pKmob vnfA3::lacZ
787	CCGCTCGCCGCAGCCGAACGTTCGCCGTCCTCGACCGG	Construction of pKmob ΔvnfDGK
788	CAACTCACCACATGATTGAAGTCTCCTCGGCTCTATG	Construction of pKmob ΔvnfDGK
789	TTCAATCATGTGGTGAGTTGAGGTGCCG	Construction of pKmob ΔvnfDGK
790	TCCGGGGTCAGCACCGTTTCAGCGGATCTCGGCGACGA	Construction of pKmob ΔvnfDGK
805	CCGCTCGCCGCAGCCGAACGAGCCGGTACCGTGGAAGATC	Construction of pKmob ΔnifDGK
806	AGCGTACCAGCATGGGTATGACTCCTCTTGC	Construction of pKmob ΔnifDGK
807	CATACCCATGCTGGTACGCTAAGTCGTCGGTTC	Construction of pKmob ΔnifDGK
808	TCCGGGGTCAGCACCGTTTCTCGCGGGGCGAGATCTGG	Construction of pKmob ΔnifDGK
813	TTCCCGACTGGAAAGCGGGCGCACTGCCTCTTCTCGGC	Amplification of *algU* for the construction of pUCanfA::lacZ
814	ACAGCCTTCTATGATCAGCTTAGCCTGCTG	Amplification of *algU* for the construction of pUCanfA::lacZ

815	AGCTGATCATAGAAGGCTGTCCGGCCCG	Amplification of *anfA* promoter for the construction of pUCanfA::lacZ
816	TCATGGTCATGCCAATCCGCCTTGTTTATCCATTGG	Amplification of *anfA* promoter for the construction of pUCanfA::lacZ
817	GCGGATTGGCATGACCATGATTACGGATTC	Amplification of *lacZ* for the construction of pUCanfA::lacZ
818	TTATCTAGAATTATTTTTGACACCAGACC	Amplification of *lacZ* for the construction of pUCanfA::lacZ
819	GTCAAAAATAATTCTAGATAATTCTTAGGCCAC	Amplification of *dhfR*II for the construction of pUCanfA::lacZ
820	CGTCGTCCACCACGAACCCAGTTGACATAAG	Amplification of *dhfR*II for the construction of pUCanfA::lacZ
821	TGGGTTCGTGGTGGACGACGTCCTCCAG	Amplification of *mucA* for the construction of pUCanfA::lacZ
822	CGGGTGTCGGGGCTGGCTTAATGTCCTTGGAAGAAGGCTC	Amplification of *mucA* for the construction of pUCanfA::lacZ

Table S5. MEME analysis of VnfA1 ChIP-seq targets identified in cultures grown in the presence of V

Identified motifs

Locus tag	Strand	Position	P value	upstream	Sites	downstream
Avin_00450	-	99	8.45E-07	AATGGTTTTA	TGTTCGCAAAGGTCC	GCTCCAGAGC
Avin_00450	+	201	2.98E-06	ggtatccgaa	TGTACGGACCGGAAC	actggaccgt
Avin_00690	+	222	3.31E-06	tgtacgcaca	TGTTCGGCCGTGAAC	ggcggacctc
Avin_00690	-	203	9.94E-06	CGAACATGTG	CGTACACAGAGGTAC	GGGAACGCGC
Avin_01300	+	162	1.45E-06	gtacattcgc	CGTTCGGAACGGAAC	gttttcccct
Avin_01300	-	142	5.61E-06	GAACGGCGAA	TGTACGCAAGTGTCC	TCCTGCCATC
Avin_01300	-	245	6.19E-06	GGCTACAGGC	TGTACGCCGGAGTAC	GGAAACGGAC
Avin_02430*	+	215	2.47E-07	cgcgatgaaa	TGTTCGGCAAAGAAC	atttcgcttt
Avin_02430*	-	185	3.74E-05	TCGCGGCAGA	CGTACCGGAGCGAAC	CCTCGGCGCC
Avin_02450*	-	132	2.47E-07	CGCGATGAAA	TGTTCGGCAAAGAAC	ATTTCGCTTT
Avin_02450*	+	162	3.74E-05	TCGCGGCAGA	CGTACCGGAGCGAAC	CCTCGGCGCC
Avin_02610	-	140	1.10E-06	AAAGGTTTTT	CGTTCGGCCAAGTAC	GGAGAGCGGG
Avin_02610	-	110	5.13E-05	GCGGGGGGTC	TGTACGTGGCTGTGC	GTGGCCGTCG
Avin_02660	-	22	6.46E-07	GGAAGCGATC	CGTTCCGCATGGTAC	TGGGGTGCTC
Avin_02660	-	52	3.71E-06	GCTCCGTCGA	CGTACCCGCAGGTAC	AGCCGGGAAG
Avin_02770	-	56	1.63E-06	GAACGATCCC	TGTACGCGGTGGTAC	TTTCACGCGG
Avin_02770	+	76	2.11E-06	GTACAGGGAT	CGTTCGCCACGGTAC	GTCCCCTCCG
Avin_07600	-	128	2.35E-06	CTTGGCAAAG	CGTTCCGGATTGAAC	AGATCGCCGA
Avin_13040	-	157	1.80E-05	GGGGGCGAAT	CGTACTGAATGGTGC	TTGCCTTGAC
Avin_17940	+	137	2.35E-06	CGGTTCCTTT	TGTACGCTGAGGTAC	AGGCGGTCAA
Avin_17960	-	639	2.35E-06	CGGTTCCTTT	TGTACGCTGAGGTAC	AGGCGGTCAA
Avin_20480	-	89	3.31E-06	GAGGCGAGGA	TGTTCTGGGTTGTAC	TATTCACCTC
Avin_26410	+	180	6.46E-07	GCGAACGGAT	CGTTCGAAATTGTAC	GCTAATCATT
Avin_30860	-	194	6.46E-07	AGCGGTCGAA	TGTTCCGGGAAGTAC	TCGAATACTG
Avin_30860	+	221	1.10E-06	cgaccgctat	CGTTCGGCGAAGTAC	gcggaaaacg
Avin_30860	-	150	1.53E-05	CGATGGAGTG	TGTTCGGAAGTGTCA	GCGGGGAACA
Avin_33440	-	111	1.53E-05	TGACGAATTT	CGTACCGAAAAGAGC	AAAGCACCAT
Avin_36070	-	129	1.18E-05	CCGTTCCGCT	TGCACGTGAATGTAC	GATTCTCCTT
Avin_39360	+	143	2.47E-07	GTCGCGGAAC	TGTTCCGGCAGGTAC	TTCGGCGGCA
Avin_39610	+	166	8.45E-07	TAACCGGCGC	TGTTCGGAATTGTCC	GATCGTCACC
Avin_39890	+	485	1.45E-06	tcaatggctg	TGTTCCAGGAGGTAC	tcggccgggt
Avin_39890	+	454	1.53E-05	acgagcacgc	CGTACCAAAAAGTAA	gggttctcaa
Avin_41400	+	129	6.46E-07	CTGTTGATCA	TGTACAGGAATGTAC	CTAAGCTCGA
Avin_41400	+	89	2.27E-05	CCTTCCTCCT	TGCTCGGCCCTGTAC	CGAAAGCTGT
Avin_47110*	-	210	1.95E-05	CGGCCCGGGC	TGTTCCGGCCGGTGC	GCAATCGGCC
Avin_47120*	-	291	1.95E-05	CGGCCCGGGC	TGTTCCGGCCGGTGC	GCAATCGGCC
Avin_47130	-	453	3.71E-06	CGGCATCGCC	TGTACTGAAATGTCC	TCCGGCGGAC
Avin_47130	-	525	1.53E-05	GGCTCTGGAG	CGTTCGAATGGGTAC	GGAGTGGGGC
Avin_47130	-	423	4.54E-05	CGGACAGGGA	TGTTCGCCTGGGTAA	TTCGCGCAAT
Avin_48350	+	152	1.98E-09	ATCGGTACGT	TGTACGGAAATGTAC	CGCCGGGAGG
Avin_48880	+	90	6.84E-06	GAAGCGAACG	CGTTCCTCAAGGAAC	ATCCCGAGGT
Avin_49190	-	144	2.11E-06	TCTTCGAATA	TGTTCCCTATTGTAC	CTTTGTATGT
Avin_50990	-	94	8.27E-06	CGCCACGGTC	AGTTCGGCCAGGTAC	AGGGTCTTGT
Avin_50990	+	183	5.46E-05	ACCAGCGAAT	TGCACGAACTGGAAC	AACGCGTCAA

* Please note that MEME identifies duplicate sites in divergently transcribed genes

MEME logo

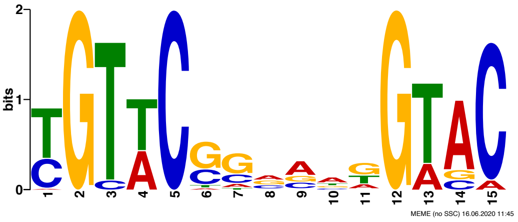

Sequences used in the search

>Avin_00450
gggcaagagactgatttcatcatgggtatcgatcatcaccggctgttgccgaacggacag
tgcaaccccgatgccacccgctcgcagcgctctggagcggacctttgcgaacataaaacc
atttttgttcgaacgcgatcaggtaatggccggccttcggcggggagtggattccggaac
gaacatcatcggtatccgaatgtacggaccggaacactggaccgtgcgagccgcaaggca
tgcctgcggctctgcccaagcgcgctccgccaccggtcgccgccaccgccggccgttgcg
gaagcggccatactggaagacggaggtgagttcatgacgagtgccgcgcagat
>Avin_00690
ggcgggtgtgggcgtcatgctttccagcggtcgttgcggcgcttccggggctcccggcga
tacgagccggatgcccgtcggaatcgcgaaaagtggcccgcaatcgggtggaatacctgg
agtgaagcaccctttccaagaccgccgcgccgggagggacgtggagattccgtgttcccg
ccgggtggatgcgcgcgttcccgtacctctgtgtacgcacatgttcggccgtgaacggcg
gacctcggtataacgttgaaaaagaaggagatttgtctacatcagggaactaaacgcttc
tttcatacccggacgtgcgttttcgcgcgtggcatggagcttgccctgagtcaggcaatc
ggccatatcgccggtttcccgaaaattctccaagcgacgacgcaaaggaacactgcatga
atcttcatcctcgcctcgccaccggcctgatcgccgctctgttaatgctcggtacgagcc
atgcgacagcccactccaacgaaacgccgacctacggcagtat
>Avin_01300
gcgttccagcatctcttggggggtcggcatggcggatggtccttcttgtggggagcgcac
gaaggatacggcaaggccgcaaccggcgcacggcggtcgcggcgccgcaaaacaggcccg
ccacgctccgtgatggcaggaggacacttgcgtacattcgccgttcggaacggaacgttt
tcccctggtagcgttgggtcgcaatccggtttttttgacgctcaggtaccgaagtccgt
ttccgtactccggcgtacagcctgtagccatggcacggagcttgcgcttggacctttcag
gttcaatcaatctcccaaggaaacaggaacatgaaaagactgctg
>Avin02430
ggcgctgcccagcttgcgcgcgccgatcaggcggcgggtggcgttgacgatgccgcgtga
cttcatggcgtgttcctcgtgcggatggatgcgccggggacgatgccagcggcgtgccag
aaccccgcggcagccgccggccgggccgcgtccggtcccggatgccgatggcacggcgcc
gagggttcgctccggtacgtctgccgcgatgaaatgttcggcaaagaacatttcgctttt
gtttccgccacgcgccgggcgcccgtggcaccggcccgacgcggtctcgggcgctgacgg
gccggctggcccgaatctcgcttaaaaccgattcatcggctggaaaacccgcatcgcgac
tttccagccctccgcacatcgctacggagccgaatccccgcccgaagctggagcgaagcg
aaatccgaacagccgccggaaggaggtcagcgtgacgacaccacagcacatcaccgccga
caccgcctatcacgcgttgc
>avin_02450
gtcgcgatgcgggttttccagccgatgaatcggttttaagcgagattcgggccagccggcccgtcagcgcccgagaccgcgtcgggccggtgccacgggcgcccggcgcgtggcggaaacaaaagcgaa
atgttctttgccgaacatttcatcgcggcagacgtaccggagcgaaccctcggcgccgtgccatcggcatccgggaccggacgcggcccggccggcggctgccgcggggttctggcacgccgctggcatc
gtccccggcgcatccatccgcacgaggaacacgccatg
>avin_02610
cggcgcggaagtggcgatccccagccgcacgtccccatggtttccgggtctggaaccgtcccccggacttcccgtacggacgcccggaagccaccgccccgacggccacgcacagccacgtacagaccc
cccgctctccgtacttggccgaacgaaaaaccttttacgtaacccgccggccgtccacccggttgtcccgaacaggaaaaaaaagcccggaaaaggcttgccccggcgcttttccaaaacctcgaaaatg
cgcacaaataattgattcgaaaggattaatctgagacagcggcgatgccgcgaaaaattcctgcaagcgctgcaaggacatatggcacgcatcctgcccttacctcttcgccaaccggtttttccggtcccg
gtcgagtgcgtcaggggactcgatccacgcatagagccgaggagacttcaatcatgccaatggtattgct
>avin_02660
ccggcgccgtcgagcaccccagtaccatgcggaacggatcgcttcccggctgtacctgcgggtacgtcgacggagcgcacagcatcctggcctggatttatggagtccaataaacctgcaaaaattaaaa
taattcactaattaaatatgtttttttgattttatattccaaaaaataggcaatcatcgacttatcgatccttgtggcaccgcccttgctcaactctcgtcggcacaaatcaaaacgccaacgaatcaacggag
gttcctaagatggcattgcgtcagtgtgcaatttacg
>avin_02770
gtacaacctcaactacaaggactaccgctgagcgcggcgtaccgcccgcgtgaaagtaccaccgcgtacagggatcgttcgccacggtacgtcccctccgaaaaaacccgccggaacccttcagggacg
cgggtcttgcgcgattggcgcgacctttgctccagctcctgcaggggttcgcgacggggcgctccgcccggcggaccgcacctttccatccaccgcacgcgacgggcatcgcccgtccggagagggacgat
gaatcagaccgaaatccagaacctgctcg
>avin_07600
atcacgtggccctggaccaggcgcgcgaagccgttcgctccgaaggcaagatcggcaccaccgggcgtggcatcggccctgcctacgaggacaaggtggcgcgccgcggcctgcgcatcggcgatctgt
tcaatccggaacgctttgccaagaagctgcacgaactgctggaataccacaatttcgtcctgcagaacttctacaaggtcgagccggtggacttccagaagacgctcgacgaggcgctgggctatgccga
gtcgctcaagccgatgatcgccgatgtcgccg
>avin_13040
agtcggtatgatttgagagattttctggtgaggatgcctagcccgctagaaatcaggtaggggatgcctagcctaccgatctcccgccgttgcggacgcgctgacccgagcccccgatagcgtctgtttccg
gccgccttggccttgtcaaggcaagcaccattcagtacgattcgccccccttattttcagggtggcccctgaggctataacgaatgaaaacttttactgcgaaaccggaaaccgttaagcgcgactggtatgt
cgtcgacgctgccg
>avin_13080

agtcctgtcaagtccatgcgctacaagggctggctgagcaagttgtggctgctggtattctgtgtctctttcctgattctcggtgtgcttggagtgctttctcccacgccgggtcgcaccctgctgtcgcaggtat
gcacggttctgtacttcgcattcttcatcctgatgcctttctataccaggatggaaaagaccaaaccggttccggaaagggtgactggctgatgaaaaagcaatttgctgcgttaattcttgcaatcctgccg
gccttttcctttgctgcggggaccgctgtcgaactg
>avin_17940
gggattcatgggtttcccggctggggtccggtttggccttgccgttcgatcctgcgactcgcgtgaccatggaagcttctcctctgccaggggtgatcacccgaaatgaaaaaccggcccgaaggccggttc
cttttgtacgctgaggtacaggcggtcaaaccacctgcacttcgtcggcctgcatgcctttctggcctttcaccgccacgaaggaaacccgctggccttcctgcaggctcttgaagccgctgctctggatgga
gcggtagtgaacgaacaggtcggggccgctttccggggt
>Avin_17960
cgccacatcgtcggacagcatgtggctgccgatgaaggtatcgatgctggaagggtccga
ctgcaagcccagttgatcgaacagtgtggacaggttgcggatgggcatttccatctttct
tcctcctctgttgcctgttggggaacgcgcgtgtcgcttccttttaaccatagcggaaaa
tccgccgacggccgcttgacccagggcaacctccagcacggagcccctcccatccatcga
ctggcggagaaaaggctaggtgatccacctgcgaggaggtagagtaactccactgtgctg
catgcattaccgtgaaacgatccgctcgaagttattccgatccttttttcgattgctcttt
gcacccagtcaacgcccggaccgagccggtccggtatccaactttgtccaaggagacaat
ccatgtccaatcgtcaaaccggtaccgtcaagtggttcaacgatgaaaaaggcttcggct
tcatcaccccggaaagcggccccgacctgttcgttcactaccgctccatccagagcagcg
gcttcaagagcctgcaggaaggccagcgggtttccttcgtggcggtgaaaggccagaaag
gcatgcaggccgacgaagtgcaggtggtttgaccgcctgtacctcagcgtacaaaaggaa
ccggccttcgggccggtttttcatttcgggtgatcacccctggcagaggagaagcttcca
tggtcacgcgagtcgcaggatcgaacggcaaggccaaaccggaccccagccgggaaaccc
atgaatccctcgatgccctgatttccgcttttctccgaagcggtggcgagattcagcaga
tcgccaacggcgtcagcggccaggtcgccggcccgcccagccgccacatcaagctcggca
agaagtagccggagccgccgcctcaggattgccgatagcgcaccagccgccgcagcagcg
gccggcccgccaggtgcagaaacaccggtgcgccggcgatcaggtagaagtaataggtga
cggcgcgccagatcaggatcgccgccccgctggtcgacttgccgaccagcggggcgagca
gcgccgccgaggccagctccgccccgcc
>avin_20480
aaaggtattgatccgcaacgaaaaagcctggccgtcggcttgacctggcaacatccatcgcgcactcttaacgatgatgaggtgaatagtacaacccagaacatcctcgcctctctgggagaaaggttca
atgcgacgttaaggaagtagtgtatgggggctctgacgaaagctgagatggcagaacgtctctacgaggagcttggcctgaacaagcgggaagccaaggaattggtggagctgttcttcgaggaaatcc
gccaggcactggagcacaacgaacaggtcaaattgtccggctttgg
>avin_26410
tgcaacgaatggctcgatgacgacgacacaaccaatctatatgaatgtatacatatcgatatcgacaggaagcgcaggaacacctttggcagacggcctgcaaggcactctaaaaataaaaaatattca
atatatttcatacagatagccaagcaaaagaactatcgtcgcgaacggatcgttcgaaattgtacgctaatcatttttctatgtataaggatatatttcgggcgttctgtcccatccggccccccttcaggaaa
ccacgcctgctcgaactgcccgtagcgctccctcgatcgggtcgatacgg
>Avin_30860
acgctacttccgcgccggcccggcagcgccgcccccggcgaaggaaataccgtcccatgt
gctacctgcggtttccaatccgggtgatcgacaggcctacggatcgctcgccgaatcgcg
acacgaaagtacaaccacctgttccccgctgacacttccgaacacactccatcgatttcg
cagcagtattcgagtacttcccggaacattcgaccgctatcgttcggcgaagtacgcgga
aaacgactttcgcggactggcacaccccttgccccatggcgcaataaattgcgtttacaa
ggagcaagaatgtctctctatcgacaggctgtcgtcgcctacctgggattgctgctgggc
acctcatgcaacagcatggccgaagaagcc
>avin_33440
tccctcttggtgacctatgtgacctatccgggattccggctggatatcgactgcaattagtaactgcctgacctgagcagggaaagcgaccgcttttcgaatggtgctttgctcttttcggtacgaaattcgtc
aagagcgttctcatatagaccaatcaaattgagcattgaggcgagacacgtcatcggcttggccatatccgatacctcagcgtggagcgattgcggcggaatttgcggactttcaccgccaggaatggaac
ttgctggtacctcttggatacggccctatccgcggccaatgaatgtc
>avin_36070
ctgcacgatccccggtcgggatgtgtatccattagagtgcgaaagaagaagcatcagcaattgctcgcgaagacggtatctgatcttccacgcttcgcaggcacccgcaggccggctcaaggagaatcgt
acattcacgtgcaagcggaacggagattgcgttccgtccagcacaagggccggagacttccactccgccgccggtgctgatgcactacgcttcagatctgcgggaatttcaagtcctagaagataggtcag
agggcctcgccgcttctgagcataatcacaagcagcacca
>avin_39360
acgccgcccggcgcggcgacgcgaatcgccgcgcaccgctcagaggatgttggcgtagtccgcctcgatccgttccatgctgaggtgattgaggaagttggagaagcacatccaggccgacagggcattg
aggtcgcggaactgttccggcaggtacttcggcggcaccaccagcccctggtccaccagttggcgcagggtacgcatgtcctccagggtggtcttgccgcagaacagcagggggatctgctccagcttgcc
cttgcgtaccgccaactggatgtagttgtaga
>avin_39610
caatacacgggtcgcacccggcgacagcggagttccaatacgggacatgggatttccttcaacatgcgtacgtcccgtccacagcggcggacgggaacggacgcaacctatgagaacaggccctcgacg
cgggcccgctcggcgcacaatgccattaaccggcgctgttcggaattgtccgatcgtcaccagcacccgatttcgttcggcacacgccgacatacgggacagacaccggcatccggaatggcaggatgga
tcttccttcggccctccgccacagaccggctcccaccactcgcatgcgagcagccgacgacaa
>Avin_39890
gcgagaccgtgtgggcgccgcggttctcgaacaactcgcccgaggtgacgttcagcgaat
aaggagtatccctgagcggcaccttgcccagcgggccgacccgcgcggcctcgtggcgat
agccgctttccgcgctgccctcgctttcttcctgccagtcctcgacctgcagcgtgccca

actccacttccgagccctgctcttcctcgcccacctcggcatgcgccagcggcaggacga
ggctgacgatcaccaacggaacccaccgacccggatgcgtgactctcggcatctggtact
tcccctcactgcgtacacggatgctccggagcggggcatcccacgcagggcaaggagcaa
gcgccataccagcgactttttgcgcaccggcgggtgcaaacgaattccttgttactttt
tgtaaaaaatttccgcttatccgacgagcacgccgtaccaaaaagtaagggttctcaatg
gctgtgttccaggaggtactcggccgggtggacggcaggacaggcgcatcgagcggcgaa
aaagtcgggcccggaccggtgggatcgggcgggcggcggcatgcctgccggtcctcggcc
ttcacgcatttcccggctcgtccacggcctcccctgcggcccgcgcagatgcgcggcga
ggtagtcgatgcaggcgcgcacgcgcggcgccacatggtgcttgtgcgggtagatcgcat
gcagcggcgcggccgggat
>avin_41400
cgcccgacggccaccgcatggcggccgtcatcgatgcccgcccctccaccctcccgtcgcgcggccatgccatgttttccttcctccttgctcggccctgtaccgaaagctgtacaaactgttgatcatgtaca
ggaatgtacctaagctcgatgcatacttatacaaatcatgaaagctgtacaggtattcgcgaggtcgtccatgtccaccgccagaccgaagatcgccatcagcgcctgcctgctcggcgaacgggtgcgct
tcaatgccggccacaaggagtcgcggctgtgcagccgcga
>avin_47110
gttccaccaccgcccgggattcaccgggcagcaatccgcatctgagaactttcataatcggttaattaaagcatctccggacagggctcacgccgcctgggcggcatccgcagaagatacagcaagctcc
gttccagtcgccgtgccttctcgattggtcgccgatcgggaccgaatgcacgaacgcgaactaaacaccggccgattgcgcaccggccggaacagcccgggccggcgacacgcggggaagagcccgccg
agtgcggcggcgaaggcccgc
>Avin_47120
ccacgtaggtgctaccgaccagcaacttcaccggcagacccaggcggcgctccagactgg
cgcgcaggggctcgttgagtcgttccaccaccgcccgggattcaccgggcagcaatccgc
atctgagaactttcataatcggttaattaaagcatctccggacagggctcacgccgcctg
ggcggcatccgcagaagatacagcaagctccgttccagtcgccgtgccttctcgattggt
cgccgatcgggaccgaatgcacgaacgcgaactaaacaccggccgattgcgcaccggccg
gaacagcccgggccggcgacacgcggggaagagcccgccgagtgcggcggcgaaggcccg
ccggacaccggccccggccacgcggcgagcggcaggggcgaagggccgcgacgctggcgc
cgatcttgcgaggggcgcctgtcccctgtccaggagcaaccgccatgtccgaatccgct
tcgccgcagccagccagcgcccgga
>Avin_47130
cgcgggcccttgcggagcgcggagaaattctccggcccgccatgacccgaagcacccacg
gtgggcgaaccgccgttccggcacggcgcaccgccctgcgtcgcgccgatgaaccacacc
gcgcaatcctcccggaacggcgaggcatcgtttacaggccgttgcgatgccgatgcgcaa
cgcatcggcaggcgccgtcgcggagcctcgacaggacgactcgaggcatgtccggcgacg
ctccccgatttcccccgcccctctatccatccagccccgccggccatcacccacgaccca
gcgaatcgcatgcgggaacggctcgccgaacgatcatgtaagggcgaacgtatacatgca
agatacctgcgttaatcaatcttttcctggcttatcgcaccacattcgcaccattgcgcg
aattacccaggcgaacatccctgtccgccggaggacatttcagtacaggcgatgccgcct
tttcccgcgatttttcgacatccaggaacccaagccccactccgtacccattcgaacgc
tccagagccatggcacggggattgacattaagcgtcatcccaaccacaagcctcaggaac
atcgtcatggctcgcttgaaggtcccctgcttactcttcgcccccctacttctcctgccc
ggcggcccagcccaggcaaccgacgatctcaagctcgccgccctg
>avin_48350
caggtccagatggccgagcagtccgccaccggctccggcggcgaccagcagggccagccactgcagcgcggcgggccaggttcggagcgaaaagcgcggcgactgcgacaacccggattctccacgg
cgaaggtaagactcatcggtacgttgtacggaaatgtaccgccgggagggattatcgaccgcgccattgatcaggtctatccgctgccggatagccattggtggcaaccaatgctcatcgcgcgtcgtcgc
ccgcctccagcgctgccagggcggcggccgcgtccatcagttcctcctcgctgaac
>avin_48880
tggcgcaatgaatgattgaaattttaaacaatcaaggtgttacatcatgtcggtacccgcgcgtgccgttcagctcaacgaagcgaacgcgttcctcaaggaacatcccgaggtgctgttcgtcgacctgct
catcgccgacatgaacggcgtggtgcgcggcaaacgcatcgaacgcaccagtctgcacaaagtatatgaacagggcatcaacctgcccgcctcgctgttcgccctggacatcaacggcgccacggtcga
gcgcagcggcctgggcctggacatcggcgattccgac
>avin_49190
ccatcaggacaacaccggcatcggcgcctcattcgccagcggctggcagcgagaaaagacctggatacttctattcctggcaaccgtcgcattgctcacttttagtttcatattagagtcactcatatataaa
acatacaaaggtacaatagggaacatattcgaagaaaattggaaaatcctggtctggatccctgtccttggtgcactctgggcacccatcgtcatatacctgtacagacgttggagaaaaaacagcgttcg
ctacagctattggatagtg
>avin_50680
ggtgtccagggacatgcgggcgaggaaacgggtggcggtcatcgtctggtagcctgcaatttatctatttatccagttttatataaagcaattcacatgccaagtgtcactttgttattattccgctgctataa
cagcgttgtctatactttcgactcacatcaattgtcatttttacgacaatttatagtgcaaatgatatagcggttgtgccaaaccctgcactccatcggacgaggaacacagcatgaaaatcagcgcccgcaa
cgtcttcaaaggtaccgtcagcgccctgaaggagggcgcgg
>avin_50990
gccgcgcctggtctaccaggccctgtggggccggctggcgcagaagaagccctggtccggcgtgctggtcaaccgccgcaaggacaagaccctgtacctggccgaactgaccgtggcgccggtgctcaa
cgaggccggcgagaccatctactacctgggcatgcaccgcgacaccagcgaattgcacgaactggaacaacgcgtcaacaaccagcgcctgatgatcgaggcggtggtcaacgccgccccggcggcga
tggtggtgctcgaccgccagcaccgggtgatgctctccaacccgagcttctgccgcctggccc
>avin_50730

Gaacgagccggcgacggccagccagaaacgaaaagctcgcacgctacgacggagagcctgcgcgaggcgggtggtcggctgcatattgtcatggctccatatctatatacaaaaagccataaggcaca
tgtcatgccaactcaaagaggcggtcgtttaaaattaaaaccaataaaatcaattatttattaataaaaaatacgacctgacatcataaagacgctcgaatacagacaattgttatctgtataacaaacaa
tacaaataagatgtcggaaaatcgacaggacatgcgtctccgtccgatccagccgatcg

Table S6. MEME analysis of VnfA3 ChIP-seq targets

Target sequences were filtered by ChIP-seq *P* value < 3 e-13

Identified motifs

Genome location	Strand	Position	*P* value	Upstream	Sites	Downstream
44986	-	165	8.96E-07	GCGCTCTGGA	GCGGACCTTTGCGAACA	TAAAACCATT
44986	-	62	1.10E-05	CGGTATCCGA	ATGTACGGACCGGAACA	CTGGACCGTG
44986	-	279	2.38E-05	GCAGC	AAAAACAGACCGAAACG	CGGGCAAGAG
45106	-	45	8.96E-07	GCGCTCTGGA	GCGGACCTTTGCGAACA	TAAAACCATT
45106	-	159	2.38E-05	CCCCAGCAGC	AAAAACAGACCGAAACG	CGGGCAAGAG
70936	-	58	3.63E-05	CCGTTCACGG	CCGAACATGTGCGTACA	CAGAGGTACG
128986	+	187	4.96E-07	CCAGGGGAAA	ACGTTCCGTTCCGAACG	GCGAATGTAC
128986	-	209	1.52E-05	GTGATGGCAG	GAGGACACTTGCGTACA	TTCGCCGTTC
233716	+	143	7.98E-07	CAAAAGCGAA	ATGTTCTTTGCCGAACA	TTTCATCGCG
251296	-	12	3.84E-06	GGAATGTGAC	AAGGACATACCCGAGCG	CCAGAAACAC
253546	-	222	2.12E-06	TCGAGCACCC	CAGTACCATGCGGAACG	GATCGCTTCC
262126	-	196	7.12E-07	GCCCGCGTGA	AAGTACCACCGCGTACA	GGGATCGTTC
262126	+	174	8.96E-07	TTCGGAGGGG	ACGTACCGTGGCGAACG	ATCCCTGTAC
262216	-	106	7.12E-07	GCCCGCGTGA	AAGTACCACCGCGTACA	GGGATCGTTC
262216	+	84	8.96E-07	TTCGGAGGGG	ACGTACCGTGGCGAACG	ATCCCTGTAC
718846	+	106	6.63E-06	CATCGGCGAT	CTGTTCAATCCGGAACG	CTTTGCCAAG
886756	-	235	2.99E-07	GCGATGTACG	AAATACCGATCCGAACG	CTTGACTCCT
886756	-	173	5.37E-05	ATTAATACAA	GTATACATACACAAATG	GAATCATGAT
886876	-	115	2.99E-07	GCGATGTACG	AAATACCGATCCGAACG	CTTGACTCCT
886876	-	53	5.37E-05	ATTAATACAA	GTATACATACACAAATG	GAATCATGAT
1269016	+	167	8.60E-06	TTGTCAAGGC	AAGCACCATTCAGTACG	ATTCGCCCCC
1384246	-	69	6.07E-06	TGGATGCAGT	TAGTGCAAATCCGAACG	CTCGAGGCTG
2040946	+	141	1.12E-06	ATGAGGTGAA	TAGTACAACCCAGAACA	TCCTCGCCTC
2323246	-	167	1.30E-05	AAGTCAGAGG	AGGAACCTTTCCGCACG	ATTCTGGCTT
2698876	-	169	1.00E-06	AAAATGATTA	GCGTACAATTTCGAACG	ATCCGTTCGC
3190816	+	123	4.40E-07	AGCAGTATTC	GAGTACTTCCCGGAACA	TTCGACCGCT
3190816	-	152	1.00E-06	GTCGTTTTCC	GCGTACTTCGCCGAACG	ATAGCGGTCG
3190906	+	33	4.40E-07	AGCAGTATTC	GAGTACTTCCCGGAACA	TTCGACCGCT
3190906	-	62	1.00E-06	GTCGTTTTCC	GCGTACTTCGCCGAACG	ATAGCGGTCG
3418936	+	11	7.12E-07	ATCGGCTAAG	ACGTTCATCCACGAACA	CTGATTTCAA
3674626	+	140	9.35E-06	TCAAGGAGAA	TCGTACATTCACGTGCA	AGCGGAACGG
4009696	-	156	4.96E-07	CTGGTGACGA	TCGGACAATTCCGAACA	GCGCCGGTTA
4174816	-	159	4.96E-07	CATCGAGCTT	AGGTACATTCCTGTACA	TGATCAACAG
4174906	-	69	4.96E-07	CATCGAGCTT	AGGTACATTCCTGTACA	TGATCAACAG
4900876	-	134	1.75E-07	TCCCTCCCGG	CGGTACATTTCCGTACA	ACGTACCGAT
4900966	-	44	1.75E-07	TCCCTCCCGG	CGGTACATTTCCGTACA	ACGTACCGAT
4961476	-	132	2.22E-05	GCCGGGTACG	AAGCTCTTTTAAGAACA	CTCGATTTTT

<u>MEME logo</u>

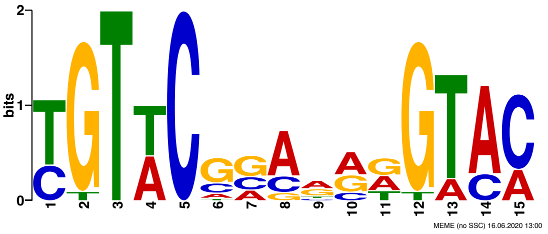

Sequences used in the search

>128986 lfc: 1.334 apv: 1.742e-61
GGGCGATCGCCAGGCATGCCAGCAGTCTTTTCATGTTCCTGTTTCCTTGGGAGATTGATT
GAACCTGAAAGGTCCAAGCGCAAGCTCCGTGCCATGGCTACAGGCTGTACGCCGGAGTAC
GGAAACGGACTTCGGTACCTGAGCGTCAAAAAAAACCGGATTGCGACCCAACGCTACCAG
GGGAAAACGTTCCGTTCCGAACGGCGAATGTACGCAAGTGTCCTCCTGCCATCACGGAGC
GTGGCGGGCCTGTTTTGCGGCGCCGCGACCGCCGTGCGCCGGTTGCGGCCTTGCCGTATC
>262126 lfc: 1.266 apv: 1.012e-55
GATTCATCGTCCCTCTCCGGACGGGCGATGCCCGTCGCGTGCGGTGGATGGAAAGGTGCG
GTCCGCCGGGCGGAGCGCCCCGTCGCGAACCCCTGCAGGAGCTGGAGCAAAGGTCGCGCC
AATCGCGCAAGACCCGCGTCCCTGAAGGGTTCCGGCGGGTTTTTTCGGAGGGGACGTACC
GTGGCGAACGATCCCTGTACGCGGTGGTACTTTCACGCGGGCGGTACGCCGCGCTCAGCG
GTAGTCCTTGTAGTTGAGGTTGTACTTGCCCATGCGCAGGCCGAGCACGCGGCGGGTCA
>4900876 lfc: 1.161 apv: 9.299e-47
CAGTCCGCCACCGGCTCCGGCGGCGACCAGCAGGGCCAGCCACTGCAGCGCGGCGGGCCA
GGTTCGGAGCGAAAAGCGCGGCGACTGCGACAACCCGGATTCTCCACGGCGAAGGTAAGA
CTCATCGGTACGTTGTACGGAAATGTACCGCCGGGAGGGATTATCGACCGCGCCATTGAT
CAGGTCTATCCGCTGCCGGATAGCCATTGGTGGCAACCAATGCTCATCGCGCGTCGTCGC
CCGCCTCCAGCGCTGCCAGGGCGGCGGCCGCGTCCATCAGTTCCTCCTCGCTGAACACCC
>262216 lfc: 1.013 apv: 2.395e-35
CCCTGCAGGAGCTGGAGCAAAGGTCGCGCCAATCGCGCAAGACCCGCGTCCCTGAAGGGT
TCCGGCGGGTTTTTTCGGAGGGGACGTACCGTGGCGAACGATCCCTGTACGCGGTGGTAC
TTTCACGCGGGCGGTACGCCGCGCTCAGCGGTAGTCCTTGTAGTTGAGGTTGTACTTGCC
CATGCGCAGGCCGAGCACGCGGCGGGTCAGGCCGAGGTGGGTGGCCGCCTCGGTCATGTT
GCCGTGGTGCAGCTTGAGGGCTTCGACGATGAGTTCGTACTCGATGGCGCCGAGGCGCGC
>1384246 lfc: 0.991 apv: 8.897e-34
AACCGGTGGATCATTCCATCGCTCGTCCGATATCGTTTCCTACAGTGTCAATCCTCGGCA
GCCTCGAGCGTTCGGATTTGCACTAACTGCATCCAGCGCCGAACGACTTCTCGACAAGGC
TTCATGCCTTGGTCAAAGTGGGAGTTTCAGGCCGTGCTCCCCAATCATCTGTACGATTTG
AGGCTGGCGTCGAGGTTCTGAGTTTCGTAGTTCAGGGATGAGTCATCTGGGACGGATTGT
CCGCTATATCAAAAGAATTGAACCACCTACCATAGTGTGGCGGCTTTCTGAGGTAATGGA
>253426 lfc: 0.988 apv: 1.384e-33
ATCACTTTCTTGCCGGCTTCGGCGAGGGCGGCGACCAGGTTCTGGGTGGTGGTGGACTTG
CCGATGCCACCCTTGCCGTAAATTGCACACTGACGCAATGCCATCTTAGGAACCTCCGTT
GATTCGTTGGCGTTTTGATTTGTGCCGACGAGAGTTGAGCAAGGGCGGTGCCACAAGGAT
CGATAAGTCGATGATTGCCTATTTTTTGGAATATAAAATCAAAAAAACATATTTAATTAG
TGAATTATTTTAATTTTTGCAGGTTTATTGGACTCCATAAATCCAGGCCAGGATGCTGTG
>2698876 lfc: 0.966 apv: 3.680e-32
GCTCGATGACGACGACACAACCAATCTATATGAATGTATACATATCGATATCGACAGGAA
GCGCAGGAACACCTTTGGCAGACGGCCTGCAAGGCACTCTAAAAATAAAAAATATTCAAT
ATATTTCATACAGATAGCCAAGCAAAAGAACTATCGTCGCGAACGGATCGTTCGAAATTG
TACGCTAATCATTTTTCTATGTATAAGGATATATTTCGGGCGTTCTGTCCCATCCGGCCC
CCCTTCAGGAAACCACGCCTGCTCGAACTGCCCGTAGCGCTCCCTCGATCGGGTCGATAC
>4159126 lfc: 0.912 apv: 1.645e-28
AGCTCACGGCGCCGGCCGGCATCGCCAGCGAGACGACGCAGGCGGCGGCCAGGCAGCGAA
GGACGGAAAGGGATGACATCGCGGCACTCCAAGATGAGCCATGGTGCTTCGCCCCGACGG
GCAAAGCCTCTATTGTCCCCGTCTCCTCCGGACCGCCGCCACCCCGAAACGCCTTTCGCG
CCGAATGGAACATCTCTGTTAGGCTCCCCGCTTGCCCCACTCCTCCAGCCAAGGAACTTT
CGATGAACGCACGCCGCGCCGTCTTTCTCGATCATGCCTCCCTCGATCTCGGCGACCTCG
>1197556 lfc: 0.911 apv: 1.725e-28
GCTCGACAGTTTCTCGGTTCTTGGCGAGAAGGATGATGGCTACAAGGTCGATCGGACTTC
TTCGCCCAAGCACACCGCGCCGTTGTTGGATACGCCACAAACCATCAGCGTGATTCCGGA
AACCCTCTATCGGGAGCAAGGCGCGCGCAACCTGACCGAGGTCCTGAGAAACACTCCGGG
GATCAGTTTCAACGCCGGCGAGAACGGTTTCTCCACCAGCTCCAACAACTTCAGTCTGCG
CGGCTTCGACACCAGCGGCAACATCTTCATCGATGATTCGCGCGACTCCGGCAGCTACAC
>3595336 lfc: 0.895 apv: 1.753e-27
TCTAACCGACTGAACTACCGCTGCGCTTGACTCCAAGATGGTGGGTGGTGACGGGATCGA
ACCGCCGACCCTCTGCTTGTAAGGCAGATGCTCTCCCGGCTGAGCTAACCACCCGTTCGC
TCTCGAAGTGGGGCGCATTCTAGAGAGGCGATTTCGCCTTGGCAAGTCTTCGCTGAAAAA
ATTTTCAGATGTTCCAAAGGGTTAGGGTTGGCCCCCAGGATCAGGAAGAGAATAATGCTC
GTTTTGCGCAATGGAGATCGCCAGCCCCATGTGGTTCCGCAACCTGCTCGTCTATCGCCT
>606736 lfc: 0.865 apv: 1.102e-25
GTCTGCGTTTCGCCATTCGCGAAGGCGGTCGTACCGTCGGCGCCGGTGTGGTGGCCAAGA
TCATCGAGTAATCGATGGGATGGTAGAAAAAGCCCCTTTTGGGGCTTTTTCTATTGCTGA

TCATTTTGTTTGACACCCCAGGGGGGCATCCGTACAATTGCGCCTCCTTTTATCGGGCGT
ATTCCGCTCGGTAGGGTGGCAACTTCGAGTCTGAGGTCAAAATGCAAAACCAACAAATCC
GTATTCGGTTGAAGGCTTTTGACCATCGCCTGATTGATCAATCTACCCAGGAAATCGTGG
>2040946 lfc: 0.847 apv: 1.454e-24
GAAGCAGCGGGGGAATACCTGACCGATCTTCGGCTGTTCGATGTCTATCAGGGTAAAGGT
ATTGATCCGCAACGAAAAAGCCTGGCCGTCGGCTTGACCTGGCAACATCCATCGCGCACT
CTTAACGATGATGAGGTGAATAGTACAACCCAGAACATCCTCGCCTCTCTGGGAGAAAGG
TTCAATGCGACGTTAAGGAAGTAGTGTATGGGGGCTCTGACGAAAGCTGAGATGGCAGAA
CGTCTCTACGAGGAGCTTGGCCTGAACAAGCGGGAAGCCAAGGAATTGGTGGAGCTGTTC
>253546 lfc: 0.810 apv: 1.866e-22
GATTCGTTGGCGTTTTGATTTGTGCCGACGAGAGTTGAGCAAGGGCGGTGCCACAAGGAT
CGATAAGTCGATGATTGCCTATTTTTTGGAATATAAAATCAAAAAAACATATTTAATTAG
TGAATTATTTTAATTTTTGCAGGTTTATTGGACTCCATAAATCCAGGCCAGGATGCTGTG
CGCTCCGTCGACGTACCCGCAGGTACAGCCGGGAAGCGATCCGTTCCGCATGGTACTGGG
GTGCTCGACGGCGCCGGACGGCCGATCGGCGAAGAGGCGAGGGGCTGGATGGTACGAGGA
>4580686 lfc: 0.810 apv: 1.866e-22
CGGCTTCTTCTGGCAGCACGGCGGCTGGACCGGCGTCGGCCTGTTCATCGCCGCCCTGCT
CGGCCTCGCCCTGATCGTGGCCCTGCGCCTGTCGCGCCTGCCGCCGAAGCAGGCACTGGC
CTGAATCCGCGAAACGCAAAAGCCCGATCGGGCTTTTGCGTCATGCGCGGACGTTCAGCG
GTGATATTGCGCCGACAGTTCGTGCACCGCCTCGAAGAAGGCGCCGGCATGGGCCGGATC
GACCTCGGGGGTGATGCCGTGGCCGAGGTTGAAGACATGCCCGGAGCCCGCGCCGTAACG
>2032126 lfc: 0.802 apv: 5.565e-22
GCGGCCTCCGGTTTGTCCTGGCTTCTCAAGATGCCTGCGGCATTCGCGAGCTGTCATTTC
GCTGGTTTCATTTCCCGCCCATCGTACATTTTCGATTTGTGCACTGATTCCCTGAAAGCG
GAGAGTTTCGTTGATAAGCTGAAAAATCCTCTCCCAATACGGCAATCAGGAGGTTCCAAA
TGCTGCTTACGGGAATCGTCGTGGGGAACCACCGGACTATTCCGCGCTATGCCATCCAGA
CCGAGGAGGGATATGTGGTCGTCGATGTGGAAAGCGGTGATCTGGCTTTTCAGCATGTGG
>4580116 lfc: 0.793 apv: 1.629e-21
GGCGCTGGCCGCCCTCGGCCTTCTGGCGCTGCTCGCGGCACTGACGTTCTGGCGCCTGCT
GCCGGATTCGCGGCACTTCCAGGCGACCCCACTGAGCTTCGCCAACCTGGCCCACGGCCT
GCGCCTGCACTTTTCCGACCAGGGCCTGCCCTGGCTGTTCCTCGAAGCCTTCCTGCTGGT
CGGCAGCTTCGTCGCCCTGTTCAACTACATGAGCTTCCGTCTGCTCGAGCCGCCCTACGG
ACTGAGCATGAGCCTGGTCGGCCTGCTTTCGGTGGTCTACCTGTCGGGCACCTACAGTTC
>1784026 lfc: 0.761 apv: 8.153e-20
AAAGCGGAAATCAGGGCATCGAGGGATTCATGGGTTTCCCGGCTGGGGTCCGGTTTGGCC
TTGCCGTTCGATCCTGCGACTCGCGTGACCATGGAAGCTTCTCCTCTGCCAGGGGTGATC
ACCCGAAATGAAAAACCGGCCCGAAGGCCGGTTCCTTTTGTACGCTGAGGTACAGGCGGT
CAAACCACCTGCACTTCGTCGGCCTGCATGCCTTTCTGGCCTTTCACCGCCACGAAGGAA
ACCCGCTGGCCTTCCTGCAGGCTCTTGAAGCCGCTGCTCTGGATGGAGCGGTAGTGAACG
>2323246 lfc: 0.759 apv: 1.094e-19
AAGGGTTTCCGCGGTTATGCCAGTACCGTCGGAGATGAAGAAAGCGGTTCGTTTCATTGC
GCCCTTGGCCTTAAGGATATGACGATGGTTAGGCTATGATGGGACCCGGCATTGCCGGGC
ATCCTGACCCAACATTGTCACTTATTTTCACGGGTCAAGCCAGAATCGTGCGGAAAGGTT
CCTCCTCTGACTTCCCAACCCCTAGTGGAGAATTCATCTTGGTAGAGTACGTAGTTTCCC
TTGACAAGCTCGGCGTCCACGATGTGGAGCGAGTAGGGGGCAAAAACGCATCCCTGGGCG
>233716 lfc: 0.707 apv: 4.822e-17
GGAGGGCTGGAAAGTCGCGATGCGGGTTTTCCAGCCGATGAATCGGTTTTAAGCGAGATT
CGGGCCAGCCGGCCCGTCAGCGCCCGAGACCGCGTCGGGCCGGTGCCACGGGCGCCCGGC
GCGTGGCGGAAACAAAAGCGAAATGTTCTTTGCCGAACATTTCATCGCGGCAGACGTACC
GGAGCGAACCCTCGGCGCCGTGCCATCGGCATCCGGGACCGGACGCGGCCCGGCCGGCGG
CTGCCGCGGGGTTCTGGCACGCCGCTGGCATCGTCCCCGGCGCATCCATCCGCACGAGGA
>4122916 lfc: 0.702 apv: 8.398e-17
GTCGTTCTGCCGTTCACCGGCACGCTCGACGGCATCCGTGTCGTGCGGCCGGGCCAGATC
GCCGCGAATGGCCTCGGCACGCTGCCGATAGTCCCGGTGCAGACGCTCCAGGATGCTCTT
TGGATCGAATACGTCGGACATGCTCGCTCCCGAGAATCGGCCATACTCGTTTTCAGTGTG
GCCCAGACTGGGCGATATGCAGTGACCTGCATCAAACGCCCGGCCACCAGCGGGACAGCC
GGCTTGACAGCCCCACCGCATTGCAAAATGCTGCGCACCGGCCGGCGAGGACCGGCCCGT
>4578286 lfc: 0.664 apv: 6.416e-15
CAGAAACCGCTGAGATAGTGGGTGGAGTTGGCCTGGCTGTCGAGATCGACCAGCAGGGTG
CGATAGCCCTCCGCCGCGCTCACCGCCGCCAGATTGCAGGCGATGCTCGACTTACCGACC
CCGCCCTTCTGATTGAACACCACACGCCGCATGCCATCGCCTCCTTCCGCCCGATCTACC
GAAGATTCCGCAAGCATTTAACCCCATTTCCGCCGGCGCGTGCGCTTATCCCTTGGTGAT
CGCCTGCGGCGGCTGCGCCTCGCGCGCCTCCCGTTCGAGCAACTCGGCGAAGCGCCGCAC
>1072966 lfc: 0.659 apv: 9.850e-15
AGTACGCCTGCATCCTCAGTGTGGTGACCCATGGCGACAACAGCGCGGACCGCTACAAGG
CCTGTCTGACGGGCATTTCCGGGCTGGTTCACGTGACCGTGGAGGTGAATCGCTGCGAGG
TGGGACAGGCAGGCGGGCGAGGGTGATCCCTCTCACTCTAGCGACCGCGAGCGGAGCCGC

CAAGGTCATTCGGCGGAGGTATAAAAAAAGCCCGGCAGGCGCTGAAAAGTACTTTTTTCT
GTCATCCGTTTTGCTGTATCAACGAAACCAGACCGTCTGACAGACCAAGGAGGATGCCCT
>4782256 lfc: 0.654 apv: 1.630e-14
ATTCACCGGGCAGCAATCCGCATCTGAGAACTTTCATAATCGGTTAATTAAAGCATCTCC
GGACAGGGCTCACGCCGCCTGGGCGGCATCCGCAGAAGATACAGCAAGCTCCGTTCCAGT
CGCCGTGCCTTCTCGATTGGTCGCCGATCGGGACCGAATGCACGAACGCGAACTAAACAC
CGGCCGATTGCGCACCGGCCGGAACAGCCCGGGCCGGCGACACGCGGGGAAGAGCCCGCC
GAGTGCGGCGGCGAAGGCCCGCCGGACACCGGCCCCGGCCACGCGGCGAGCGGCAGGGGC
>4009696 lfc: 0.625 apv: 3.065e-13
GTCGCACCCGGCGACAGCGGAGTTCCAATACGGGACATGGGATTTCCTTCAACATGCGTA
CGTCCCGTCCACAGCGGCGGACGGGAACGGACGCAACCTATGAGAACAGGCCCTCGACGC
GGGCCCGCTCGGCGCACAATGCCATTAACCGGCGCTGTTCGGAATTGTCCGATCGTCACC
AGCACCCGATTTCGTTCGGCACACGCCGACATACGGGACAGACACCGGCATCCGGAATGG
CAGGATGGATCTTCCTTCGGCCCTCCGCCACAGACCGGCTCCCACCACTCGCATGCGAGC
>251296 lfc: 1.213 apv: 1.951e-76
TGTGTTTCTGGCGCTCGGGTATGTCCTTGTCACATTCCAGCAATACCATTGGCATGATTG
AAGTCTCCTCGGCTCTATGCGTGGATCGAGTCCCCTGACGGCACTCGACCGGGACCGGAA
AAACCGGTTGGCGAAGAGGTAAGGGCAGGATGCGTGCCATATGTCCTTGCAGCGCTTGCA
GGAATTTTTCGCGGCATCGCCGCTGTCTCAGATTAATCCTTTCGAATCAATTATTTGTGC
GCATTTTCGAGGTTTTGGAAAAGCGCCGGGGCAAGCCTTTTCCGGGCTTTTTTTTCCTGT
>70936 lfc: 1.140 apv: 8.976e-68
AGGGACGTGGAGATTCCGTGTTCCCGCCGGGTGGATGCGCGCGTTCCCGTACCTCTGTGT
ACGCACATGTTCGGCCGTGAACGGCGGACCTCGGTATAACGTTGAAAAAGAAGGAGATTT
GTCTACATCAGGGAACTAAACGCTTCTTTCATACCCGGACGTGCGTTTTCGCGCGTGGCA
TGGAGCTTGCCCTGAGTCAGGCAATCGGCCATATCGCCGGTTTCCCGAAAATTCTCCAAG
CGACGACGCAAAGGAACACTGCATGAATCTTCATCCTCGCCTCGCCACCGGCCTGATCGC
>3190816 lfc: 1.069 apv: 1.785e-59
GGTTTCCAATCCGGGTGATCGACAGGCCTACGGATCGCTCGCCGAATCGCGACACGAAAG
TACAACCACCTGTTCCCCGCTGACACTTCCGAACACACTCCATCGATTTCGCAGCAGTAT
TCGAGTACTTCCCGGAACATTCGACCGCTATCGTTCGGCGAAGTACGCGGAAAACGACTT
TCGCGGACTGGCACACCCCTTGCCCCATGGCGCAATAAATTGCGTTTACAAGGAGCAAGA
ATGTCTCTCTATCGACAGGCTGTCGTCGCCTACCTGGGATTGCTGCTGGGCACCTCATGC
>44986 lfc: 0.974 apv: 2.498e-49
ACCGGTGGCGGAGCGCGCTTGGGCAGAGCCGCAGGCATGCCTTGCGGCTCGCACGGTCCA
GTGTTCCGGTCCGTACATTCGGATACCGATGATGTTCGTTCCGGAATCCACTCCCCGCCG
AAGGCCGGCCATTACCTGATCGCGTTCGAACAAAAATGGTTTTATGTTCGCAAAGGTCCG
CTCCAGAGCGCTGCGAGCGGGTGGCATCGGGGTTGCACTGTCCGTTCGGCAACAGCCGGT
GATGATCGATACCCATGATGAAATCAGTCTCTTGCCCGCGTTTCGGTCTGTTTTTGCTGC
>4900966 lfc: 0.920 apv: 7.136e-44
CAACCCGGATTCTCCACGGCGAAGGTAAGACTCATCGGTACGTTGTACGGAAATGTACCG
CCGGGAGGGATTATCGACCGCGCCATTGATCAGGTCTATCCGCTGCCGGATAGCCATTGG
TGGCAACCAATGCTCATCGCGCGTCGTCGCCCGCCTCCAGCGCTGCCAGGGCGGCGGCCG
CGTCCATCAGTTCCTCCTCGCTGAACACCCGTACCCCGTGGCGCCTGAGCAGCGCGGCGG
TGACGCCCTCGCCGGGCACCCGCCGGCCGGCGAAACTGCCGTCGTAGTTCTCGCGATTGC
>718846 lfc: 0.901 apv: 4.049e-42
GCGCGCGAAGCCGTTCGCTCCGAAGGCAAGATCGGCACCACCGGGCGTGGCATCGGCCCT
GCCTACGAGGACAAGGTGGCGCGCCGCGGCCTGCGCATCGGCGATCTGTTCAATCCGGAA
CGCTTTGCCAAGAAGCTGCACGAACTGCTGGAATACCACAATTTCGTCCTGCAGAACTTC
TACAAGGTCGAGCCGGTGGACTTCCAGAAGACGCTCGACGAGGCGCTGGGCTATGCCGAG
TCGCTCAAGCCGATGATCGCCGATGTCGCCGCGCGCCTGCACGAACTGCGCAAGCAGGGC
>886876 lfc: 0.895 apv: 1.401e-41
TCGTCGCCCTGGCGCAGCCGGGCGAACACATGGGGCTTTCTGATCATGATTCCATTTGTG
TATGTATACTTGTATTAATGAGAATTGTTATTGCTTTGATAGGCAGGAGTCAAGCGTTCG
GATCGGTATTTCGTACATCGCGGTGAGTCGTTCCCGTCGCCGCCGCTAACGCCCGGCGGG
CAAATCCGGGCAAGATAGGCCGTTGAACAAGACAGAGAGTCCGATCGTGAATACCGACAC
CGAATCCTTCGTCGTCGCGGCCACTCCCGAGGAGGCGGTGGAGCGCCTCGCCGCCCTGCA
>3190906 lfc: 0.784 apv: 1.239e-31
GAACACACTCCATCGATTTCGCAGCAGTATTCGAGTACTTCCCGGAACATTCGACCGCTA
TCGTTCGGCGAAGTACGCGGAAAACGACTTTCGCGGACTGGCACACCCCTTGCCCCATGG
CGCAATAAATTGCGTTTACAAGGAGCAAGAATGTCTCTCTATCGACAGGCTGTCGTCGCC
TACCTGGGATTGCTGCTGGGCACCTCATGCAACAGCATGGCCGAAGAAGCCGCTTCCGAT
CCTCTGGAGCTCGGCGAAACCCGGATCGTCGACGATCAGCTCGCGGACGGCAGCGCCGAG
>4174816 lfc: 0.732 apv: 1.737e-27
CTGAGTTCCGCACCCCTCCCGCGACATATTCGCCCGACGGCCACCGCATGGCGGCCGTCA
TCGATGCCCGCCCCTCCACCCTCCCGTCGCGCGGCCATGCCATGTTTTCCTTCCTCCTTG
CTCGGCCCTGTACCGAAAGCTGTACAAACTGTTGATCATGTACAGGAATGTACCTAAGCT
CGATGCATACTTATACAAATCATGAAAGCTGTACAGGTATTCGCGAGGTCGTCCATGTCC

ACCGCCAGACCGAAGATCGCCATCAGCGCCTGCCTGCTCGGCGAACGGGTGCGCTTCAAT
>3674626 lfc: 0.729 apv: 3.218e-27
ATTTAGAAGGCAACTGCACGATCCCCGGTCGGGATGTGTATCCATTAGAGTGCGAAAGAA
GAAGCATCAGCAATTGCTCGCGAAGACGGTATCTGATCTTCCACGCTTCGCAGGCACCCG
CAGGCCGGCTCAAGGAGAATCGTACATTCACGTGCAAGCGGAACGGAGATTGCGTTCCGT
CCAGCACAAGGGCCGGAGACTTCCACTCCGCCGCCGGTGCTGATGCACTACGCTTCAGAT
CTGCGGGAATTTCAAGTCCTAGAAGATAGGTCAGAGGGCCTCGCCGCTTCTGAGCATAAT
>3418936 lfc: 0.688 apv: 3.707e-24
ATCGGCTAAGACGTTCATCCACGAACACTGATTTCAAATTTCTCGAAACCAACGACGAAA
CCGTCTCCCTCTTGGTGACCTATGTGACCTATCCGGGATTCCGGCTGGATATCGACTGCA
ATTAGTAACTGCCTGACCTGAGCAGGGAAAGCGACCGCTTTTCGAATGGTGCTTTGCTCT
TTTCGGTACGAAATTCGTCAAGAGCGTTCTCATATAGACCAATCAAATTGAGCATTGAGG
CGAGACACGTCATCGGCTTGGCCATATCCGATACCTCAGCGTGGAGCGATTGCGGCGGAA
>4961476 lfc: 0.668 apv: 1.000e-22
CCGGCCCGATGCGGAAACAGCTCGGCAAGTCACTCCGCAGCCCCTGCTCCAAGAACGAAG
AATTTACCGACCCAGCCATTTCCCCGAACTTCCATTCGGGCCAGGTATATTTGGAAAAGT
GAAAAATCGAGTGTTCTTAAAAGAGCTTCGTACCCGGCCGCACCAAAACTCGCACACTAC
GACCAAGTACACAGAACCGATATGAACATCCAACTCGAAGTTGAAAAAAGGCAATGGAAA
ACGGCCATACTTGCGTTAACTGCCGCAACAGGCCGAGCCGATCATGAGGTTTTCATATAC
>1269016 lfc: 0.651 apv: 1.392e-21
TATTTTCTCGTTAGTCGGTATGATTTGAGAGATTTTCTGGTGAGGATGCCTAGCCCGCTA
GAAATCAGGTAGGGGATGCCTAGCCTACCGATCTCCCGCCGTTGCGGACGCGCTGACCCG
AGCCCCCGATAGCGTCTGTTTCCGGCCGCCTTGGCCTTGTCAAGGCAAGCACCATTCAGT
ACGATTCGCCCCCTTATTTTCAGGGTGGCCCCTGAGGCTATAACGAATGAAAACTTTTAC
TGCGAAACCGGAAACCGTTAAGCGCGACTGGTATGTCGTCGACGCTGCCGGCCAGACCCT
>45106 lfc: 0.615 apv: 3.585e-19
AAGGCCGGCCATTACCTGATCGCGTTCGAACAAAAATGGTTTTATGTTCGCAAAGGTCCG
CTCCAGAGCGCTGCGAGCGGGTGGCATCGGGGTTGCACTGTCCGTTCGGCAACAGCCGGT
GATGATCGATACCCATGATGAAATCAGTCTCTTGCCCGCGTTTCGGTCTGTTTTTGCTGC
TGGGGTTGTCCCTGGCCACGGCCCAGGCGCGTCAGCTTACCGATATGAGCGGCCGTACCG
AGGAGATTCCCGAGCGGCCGCAGCGGGTGTTCCCCGCTGTGACCATGATGACGCCGGTGA
>886756 lfc: 0.595 apv: 7.457e-18
ATCAGCTCGTCCCCGGAGCGCAGCAGGTGCTCCATGTCGAGAATCACGAAGTGATAGCCG
GCGGCGGCGATCATTTCGCAGAGCAGGGGAGAGGGGACCGAATTGAGCAAGCCGAAGACT
TCGTCGCCCTGGCGCAGCCGGGCGAACACATGGGGCTTTCTGATCATGATTCCATTTGTG
TATGTATACTTGTATTAATGAGAATTGTTATTGCTTTGATAGGCAGGAGTCAAGCGTTCG
GATCGGTATTTCGTACATCGCGGTGAGTCGTTCCCGTCGCCGCCGCTAACGCCCGGCGGG
>4174906 lfc: 0.589 apv: 1.736e-17
GCGGCCATGCCATGTTTTCCTTCCTCCTTGCTCGGCCCTGTACCGAAAGCTGTACAAACT
GTTGATCATGTACAGGAATGTACCTAAGCTCGATGCATACTTATACAAATCATGAAAGCT
GTACAGGTATTCGCGAGGTCGTCCATGTCCACCGCCAGACCGAAGATCGCCATCAGCGCC
TGCCTGCTCGGCGAACGGGTGCGCTTCAATGCCGGCCACAAGGAGTCGCGGCTGTGCAGC
CGCGAGTTCGCCAGATATTTCGACTTCGTTCCGCTCTGCCCGGAAATGGCCATCGGCCTC

Supplementary References

Benedetti, I. M., de Lorenzo, V. and Silva-Rocha, R., (2012) Quantitative, Non-Disruptive Monitoring of Transcription in Single Cells with a Broad-Host Range GFP-luxCDABE Dual Reporter System. *PLoS ONE* **7**: e52000-e52000, https://doi.org/10.1371/journal.pone.0052000.

Blattner, F. R., Plunkett, G., 3rd, Bloch, C. A., Perna, N. T., Burland, V., Riley, M., Collado-Vides, J., Glasner, J. D., Rode, C. K., Mayhew, G. F., Gregor, J., Davis, N. W., Kirkpatrick, H. A., Goeden, M. A., Rose, D. J., Mau, B. and Shao, Y., (1997) The complete genome sequence of Escherichia coli K-12. *Science* **277**: 1453-1462, https://doi.org/10.1126/science.277.5331.1453.

Damron, F. H., McKenney, E. S., Schweizer, H. P. and Goldberg, J. B., (2013) Construction of a broad-host-range Tn7-based vector for single-copy P(BAD)-controlled gene expression in gram-negative bacteria. *Applied and Environmental Microbiology* **79**: 718-721, https://doi.org/10.1128/aem.02926-12.

de Lorenzo, V., Cases, I., Herrero, M. and Timmis, K. N., (1993) Early and late responses of TOL promoters to pathway inducers: identification of postexponential promoters in Pseudomonas putida with lacZ-tet bicistronic reporters. *Journal of Bacteriology* **175**: 6902-6907, https://doi.org/10.1128/jb.175.21.6902-6907.1993.

Jumper, J., Evans, R., Pritzel, A., Green, T., Figurnov, M., Ronneberger, O., Tunyasuvunakool, K., Bates, R., Žídek, A., Potapenko, A., Bridgland, A., Meyer, C., Kohl, S. A. A., Ballard, A. J., Cowie, A., Romera-Paredes, B., Nikolov, S., Jain, R., Adler, J., Back, T., Petersen, S., Reiman, D., Clancy, E., Zielinski, M., Steinegger, M., Pacholska, M., Berghammer, T., Bodenstein, S., Silver, D., Vinyals, O., Senior, A. W., Kavukcuoglu, K., Kohli, P. and Hassabis, D., (2021) Highly accurate protein structure prediction with AlphaFold. *Nature* **596**: 583-589, https://doi.org/10.1038/s41586-021-03819-2.

Karimova, G., Dautin, N. and Ladant, D., (2005) Interaction network among Escherichia coli membrane proteins involved in cell division as revealed by bacterial two-hybrid analysis. *Journal of Bacteriology* **187**: 2233-2243, https://doi.org/10.1128/JB.187.7.2233-2243.2005.

Karimova, G., Pidoux, J., Ullmann, A. and Ladant, D., (1998) A bacterial two-hybrid system based on a reconstituted signal transduction pathway. *Proceedings of the National Academy of Sciences of the United States of America* **95**: 5752-5756, https://doi.org/10.1073/pnas.95.10.5752.

Karimova, G., Ullmann, A. and Ladant, D., (2001) Protein-protein interaction between Bacillus stearothermophilus tyrosyl-tRNA synthetase subdomains revealed by a bacterial two-hybrid system. *Journal of Molecular Microbiology and Biotechnology* **3**: 73-82.

Lemoine, F., Correia, D., Lefort, V., Doppelt-Azeroual, O., Mareuil, F., Cohen-Boulakia, S. and Gascuel, O., (2019) NGPhylogeny.fr: new generation phylogenetic services for non-specialists. *Nucleic Acids Research* **47**: W260-W265, https://doi.org/10.1093/nar/gkz303.

Mirdita, M., Schütze, K., Moriwaki, Y., Heo, L., Ovchinnikov, S. and Steinegger, M., (2022) ColabFold - Making protein folding accessible to all. *bioRxiv* 10.1101/2021.08.15.456425: 2021.2008.2015.456425, https://doi.org/10.1101/2021.08.15.456425.

Ray, P., Smith, K. J., Parslow, R. A., Dixon, R. and Hyde, E. I., (2002) Secondary structure and DNA binding by the C-terminal domain of the transcriptional activator NifA from Klebsiella pneumoniae. *Nucleic Acids Research* **30**: 3972-3980.

Schäfer, A., Tauch, A., Jäger, W., Kalinowski, J., Thierbach, G. and Pühler, A., (1994) Small mobilizable multi-purpose cloning vectors derived from the Escherichia coli plasmids

pK18 and pK19: Selection of defined deletions in the chromosome of Corynebacterium glutamicum. *Gene* **145**: 69-73, https://doi.org/10.1016/0378-1119(94)90324-7.

Setubal, J. C., dos Santos, P., Goldman, B. S., Ertesvag, H., Espin, G., Rubio, L. M., Valla, S., Almeida, N. F., Balasubramanian, D., Cromes, L., Curatti, L., Du, Z., Godsy, E., Goodner, B., Hellner-Burris, K., Hernandez, J. A., Houmiel, K., Imperial, J., Kennedy, C., Larson, T. J., Latreille, P., Ligon, L. S., Lu, J., Maerk, M., Miller, N. M., Norton, S., O'Carroll, I. P., Paulsen, I., Raulfs, E. C., Roemer, R., Rosser, J., Segura, D., Slater, S., Stricklin, S. L., Studholme, D. J., Sun, J., Viana, C. J., Wallin, E., Wang, B., Wheeler, C., Zhu, H., Dean, D. R., Dixon, R. and Wood, D., (2009) Genome Sequence of Azotobacter vinelandii, an Obligate Aerobe Specialized To Support Diverse Anaerobic Metabolic Processes. *Journal of Bacteriology* **191**: 4534-4545, https://doi.org/10.1128/jb.00504-09.

Söderbäck, E., Reyes-Ramirez, F., Eydmann, T., Austin, S., Hill, S. and Dixon, R., (1998) The redox- and fixed nitrogen-responsive regulatory protein NIFL from Azotobacter vinelandii comprises discrete flavin and nucleotide-binding domains. *Molecular Microbiology* **28**: 179-192, https://doi.org/10.1046/j.1365-2958.1998.00788.x.

Zallot, R., Oberg, N. and Gerlt, J. A., (2019) The EFI Web Resource for Genomic Enzymology Tools: Leveraging Protein, Genome, and Metagenome Databases to Discover Novel Enzymes and Metabolic Pathways. *Biochemistry* **58**: 4169-4182, https://doi.org/10.1021/acs.biochem.9b00735.
